# The Cog‐Aging: a 15‐year cohort study of cognition in Brazilian older adults

**DOI:** 10.1002/alz.71780

**Published:** 2026-09-07

**Authors:** Gabriela Tomé Oliveira Engelmann, Joice Coutinho de Alvarenga, Giovanna Correia Pereira Moro, Ivonne Carolina Bolaños Burgos, Mariana Figueiredo Miranda, Millena Figueiredo Miranda, Aline Siqueira de Souza, Marcelle Ferreira Saldanha, Erika Oliveira Hansen, Natalia Silva Dias, Fabiana Carla Matos da Cunha Cintra, Bruna Fulgêncio Dias, Anna Luiza da Silva Souza, Andréa Teixeira Carvalho, Pamela V. Martino‐Adami, Marco Túlio Gualberto Cintra, Ludimila Labanca, Igor Nunes Lanna, Rafaela Teixeira de Ávila, Jonas Jardim de Paula, Luciana Costa‐Silva, Daniela Valadão Freitas Rosa, Débora Marques de Miranda, Marco Aurélio Romano‐Silva, Luiz Armando Cunha de Marco, Alfredo Ramírez, Bernardo de Mattos Viana, Maria Aparecida Camargos Bicalho

**Affiliations:** ^1^ Cog‐Aging Research Group, Faculty of Medicine Universidade Federal de Minas Gerais Belo Horizonte Minas Gerais Brazil; ^2^ Older Adult Psychiatry and Psychology Extension Program (PROEPSI), Faculty of Medicine Universidade Federal de Minas Gerais Belo Horizonte Minas Gerais Brazil; ^3^ Molecular Medicine Program, Faculty of Medicine Universidade Federal de Minas Gerais Belo Horizonte Minas Gerais Brazil; ^4^ Universidade Federal de Minas Gerais Belo Horizonte Minas Gerais Brazil; ^5^ Adult Health Sciences Applied Program, Faculty of Medicine Universidade Federal de Minas Gerais Belo Horizonte Minas Gerais Brazil; ^6^ Jenny de Andrade Faria Institute– Reference Center for the Elderly, Hospital das Clínicas Universidade Federal de Minas Gerais Belo Horizonte Minas Gerais Brazil; ^7^ University Hospital Universidade Federal de Minas Gerais Belo Horizonte Minas Gerais Brazil; ^8^ René Rachou Institute Oswaldo Cruz Foundation (Fiocruz) Belo Horizonte Minas Gerais Brazil; ^9^ Division of Neurogenetics and Molecular Psychiatry, Department of Psychiatry and Psychotherapy, Faculty of Medicine and University Hospital Cologne University of Cologne Cologne Germany; ^10^ Hermes Pardini (Grupo Fleury) Belo Horizonte Minas Gerais Brazil; ^11^ Neurotec R National Institute of Science and Technology (INCT‐Neurotec R), Faculty of Medicine Universidade Federal de Minas Gerais (UFMG) Belo Horizonte Minas Gerais Brazil

**Keywords:** aging, Alzheimer's disease, Brazil, cognition, cohort study, dementia

## Abstract

**BACKGROUND:**

Prevalence of dementia is higher in low‐ and middle‐income countries (LMICs). However, longitudinal studies characterizing cognitive decline and its clinical and epidemiological predictors remain scarce in Brazil.

**METHODS:**

Participants were enrolled from 2011 to 2025 and followed through May 2026. A total of 887 participants were classified as cognitively unimpaired (CU) (*n* = 274), mild cognitive impairment (MCI) (*n* = 378), or dementia of the Alzheimer's type (DAT) (*n* = 235). CU and MCI participants were followed until dementia conversion, which was analyzed under competing risks using cumulative incidence functions and Fine–Gray models.

**RESULTS:**

Overall, annualized dementia conversion rate in the MCI group was 10.72 per 100 person‐years (95% confidence interval [CI]: 8.54% to 13.29%). In addition, 40.39% of participants progressed to dementia during a mean follow‐up time of 3.75 years (standard deviation: 3.28), with an estimated cumulative incidence of 29.85% at 3 years (95% CI: 22.74% to 36.96%), **a**ccounting for death as a competing event.

**DISCUSSION:**

This study adds valuable information for LMICs. However, estimates should be interpreted considering attrition and competing mortality.

## Highlights

- MCI was associated with a markedly higher cumulative incidence of dementia compared with CU individuals over long‐term follow‐up.
- Age showed a consistent association with dementia in the CU group but not for the MCI group, remaining the main factor associated with progression in competing risk models.
- Higher educational attainment was associated with higher cumulative incidence of dementia in the MCI group, although this finding should be interpreted cautiously given competing mortality and borderline significance.
- Sex‐stratified analyses suggested higher progression rates in men; however, this association was not significant after adjustment for age and education.
- The annualized conversion rate for the MCI group was 10.72 per 100 person‐years (95% CI: 8.54 to 13.29)

## 1 INTRODUCTION

Dementia of the Alzheimer's type (DAT) is the leading cause of dementia worldwide. Currently, 57 million people are living with dementia, and this number is projected to reach 153 million by 2050.^1^ Around two‐thirds of people with dementia live in low‐ and middle‐income countries (LMICs). However, this prevalence has been underestimated, and it is predicted to rise more rapidly in LMICs than in high‐income countries. Aliberti and colleagues (2025) reached a consensus that the average prevalence of dementia in Brazilians aged 60 years or older was 8.5%, corresponding to approximately 2.46 million people living with this condition in 2019. Projections from this consensus also indicate that this number will increase to 8.89 million by 2060.^2^ This growing burden of dementia is driven by a rapidly aging population, a high prevalence of cardiovascular risk factors, limited educational opportunities, and marked social inequalities.

**RESEARCH IN CONTEXT**

1. **Systematic review**: The authors searched traditional databases, including PubMed, for longitudinal cohort studies evaluating cognitive trajectories and conversion to dementia using neuropsychological and clinical assessments. Particular attention was given to studies incorporating time‐to‐event approaches and competing risk methodology. Although several cohorts have investigated progression from MCI to dementia, most have relatively short follow‐up periods, limited representation from LMICs, and heterogeneous methodological approaches.
2. **Interpretation**: In this LMIC setting, our study demonstrated a high long‐term probability of progression from MCI to dementia, with a marked increase in cumulative incidence over extended follow‐up. Age was more consistently associated with dementia conversion among CU participants, while higher educational attainment showed a possible association with conversion in the MCI group. Given the competing mortality and borderline significance, this finding should be considered exploratory and interpreted with caution. Sex differences were observed in crude and stratified analyses but were attenuated after adjustment for demographic factors. The integration of clinical and neuropsychological assessments in a real‐world cohort of older adults with low educational attainment highlights the relevance of this study for understanding dementia trajectories and risk factors in vulnerable populations. These findings underscore the importance of longitudinal cohort studies in LMICs and contribute to the development of context‐specific strategies for dementia prevention and care.
3. **Future directions**: Future research should further explore the complex interactions between cognitive decline, vascular and social determinants, and biological markers across the Alzheimer's disease continuum. Expanding follow‐up duration, incorporating multimodal biomarkers, and improving the assessment of competing events such as mortality will be essential to refine risk stratification. In addition, addressing cognitive and socioeconomic vulnerabilities, evaluating attrition and missing data, and strengthening internal validity will enhance the interpretation and generalizability of findings. Integrating new analytical approaches and fostering longitudinal collaborations across LMICs will be critical to advancing knowledge and improving dementia care globally.

In Brazil, the illiteracy rate among people over 60 years is approximately 16.0%. Moreover, inequality can be even greater across different regions of the country.^3^ Social inequality is known to prevent access to healthcare and early diagnosis of diseases. In addition, insufficient financial investment limits research development in LMICs, as the lack of more sensitive and precise equipment and/or materials affects the scope of investigations, restricting the potential outcomes that can be achieved with the limited resources available. Consequently, research conducted in LMICs struggles to advance, leaving populations in these countries without adequate strategies for disease prevention and treatment.^4^

In Brazil, several notable cohort studies have been conducted to evaluate older adults' health, such as the Longitudinal Study of Adult Health (ELSA‐Brazil),^5^ a multicenter initiative conducted in six research centers and focusing on public employees of five public universities and one public research center; the Longitudinal Study of Brazilian Elderly (ELSI‐Brazil),^6^ which examines adults aged 50 and older across 70 municipalities in five major geographic regions of the country; and Bambuí Project, an epidemiological investigation into geriatric health based in the city of Bambuí,^7^ Minas Gerais. All studies have their scientific relevance and have different characteristics and objectives. Longitudinal studies tracking cognitive decline are scarce in Brazil. While the Cognition and Aging Research Group (Cog‐Aging) cohort collects genetic and fluid biomarkers, establishing the long‐term clinical, functional, and epidemiological foundation of this population is a necessary first step.

The mild cognitive impairment (MCI) outpatient service was established in 2010, and research in this context has been conducted since 2011. The research aimed to determine the conversion rate from MCI to dementia in a cohort of Brazilian older adults, as well as to identify the factors related to dementia conversion. Geriatric, neuropsychological, neuroimaging, and molecular assessments have been the baseline of the research group over the years with translational approaches. Initially called the MCI Outpatient Clinic, Cog‐Aging is based at the Geriatric Outpatient Service of the University Hospital of the Federal University of Minas Gerais (UFMG), which is a public reference center for secondary and tertiary healthcare for older adults of Belo Horizonte, the capital city of Minas Gerais State, and Brazil.

Thus, the scientific research developed by Cog‐Aging plays a crucial role in providing diagnosis and clinical evaluation throughout the Alzheimer's disease continuum, as well as contributing to the community, academic, and scientific spheres within the contexts of the aging global population.

This paper aimed to describe the methodology and results of 15 years of the Cog‐Aging cohort study, which has been approved since 2011 by the UFMG Ethics Committee. This paper was written according to the Strengthening of the Reporting of Observational Studies in Epidemiology (STROBE) checklist.

## 2 METHODS

### 2.1 Participants

Participants of the Cog‐Aging cohort study, an open prospective cohort, are referred from primary healthcare centers of the city of Belo Horizonte, in Brazil, for geriatric assessment at the Geriatric Outpatient Service of the University Hospital of the UFMG. In 2010, the partnership between the UFMG, the State of Minas Gerais, and the Municipality of Belo Horizonte led to creation of the Mais Vida Program, which aims to provide comprehensive geriatric assessment and healthcare for frail older adults. Individuals with or without cognitive complaints who fulfill the inclusion criteria are invited to participate in the Cog‐Aging cohort study (Figure S1).

The outpatient research clinic at the Cog‐Aging covers the city of Belo Horizonte and serves a diverse group of individuals. The research team includes geriatricians, geriatric psychiatrists, neuropsychologists, occupational therapists, speech therapists, nutritionists, and biologists. Together, they engage in research spanning neuropsychology, biology, nutrition, and functionality. Over the years, Cog‐Aging has assessed 1270 participants through June 2025. For the longitudinal analysis, the analytic sample was restricted to participants enrolled on or before June 1, 2025, resulting in the exclusion of nine individuals who had insufficient time to complete at least one follow‐up assessment. Follow‐up data were collected through May 31, 2026, thereby providing all included participants with sufficient time to undergo at least one follow‐up assessment.

The inclusion criteria are individuals aged 60 years or older, with normal cognition or MCI, who agree to participate in the project and sign a Term of Free and Informed Consent (TFIC). Additionally, they must have undergone at least one neuropsychological assessment. Individuals who met the criteria for probable DAT, with or without vascular component, at the first assessment were included in the study and subjected to the same research protocol, but they were not followed over time.

Moreover, older adults without cognitive impairment upon screening cognitive test (Mini‐Mental State Examination), scheduled for elective lower limb orthopedic surgeries under spinal anesthesia were invited to join the study and provide cerebrospinal fluid (CSF) by signing the TFIC. In this case, a brief cognitive screening is performed immediately before the surgery to assess cognitive status. Approximately 1 month after surgery, these individuals are referred to geriatric evaluation for a comprehensive geriatric and cognitive evaluation. At this visit, a detailed clinical assessment is performed to identify any ongoing acute medical condition or postoperative complications, including possible delirium or other factors that could compromise the reliability of cognitive testing. Individuals presenting clinically significant abnormalities at this stage are excluded from the cohort.

Importantly, cognitive classification is not assigned in the presence of transient or uncertain findings. Participants exhibiting cognitive alterations at postoperative evaluations are not allocated to the cognitively unimpaired group. Instead, they undergo further clinical follow‐up and reassessment before being classified as MCI or dementia, when appropriate. After reassessment, in case these individuals do not meet diagnostic criteria for being allocated in one of the study groups, they are excluded from the study. This approach ensures that diagnosis allocation reflects stable cognitive status rather than transient postoperative effects.

The exclusion criteria include individuals under 60 years old; those with cognitive impairment due to major depression; individuals with other severe mental health disorders such as bipolar affective disorder and schizophrenia; those experiencing delirium; individuals with a current cancer diagnosis, unless in complete remission; those with serious terminal illnesses requiring palliative care; individuals with infectious‐parasitic diseases affecting immunity; those with severe auditory, visual, or speech impairments that prevent cognitive testing; participants diagnosed with non‐Alzheimer's disease dementia; and those with MCI secondary to specific clinical or other mental disorders. Refusal to sign the TFIC also constitutes an exclusion criterion.

### 2.2 Follow‐up time

Follow‐up visits were categorized into bins based on the nearest annual time point. For instance, the Year 1 bin encompassed all visits occurring between 0.5 and 1.5 years (6 to 18 months) after baseline. This nearest‐neighbor approach was employed to maximize data inclusion and minimize the number of visits classified as “outside the window” (Table S1). Furthermore, this flexible time frame helped accommodate participants who missed their initial assessment but returned in subsequent years, while also accounting for longitudinal follow‐up disruptions caused by the COVID‐19 pandemic.

All participants that fulfill inclusion criteria to participate in the study and do not meet criteria for DAT at first assessment are followed annually until conversion to dementia occurs, when they are referred to the Geriatric Service to begin specialized care and treatment.

### 2.3 Proceedings

#### 2.3.1 Research protocol

All research participants undergo the same structured research protocol, and researchers are trained by the leading investigators. Reinforcement of training is carried out at the beginning of each semester to strengthen the uniformity of procedures in the research.

Participants who signed the informed consent form for the Cog‐Aging cohort study underwent comprehensive assessments conducted by geriatricians and geriatric psychiatrists, using standardized scales to evaluate clinical, functional, cognitive, and mood domains. After these assessments, individuals underwent additional neuropsychological evaluations, repeated every 6 months for the first 2 years and annually thereafter. Furthermore, participants underwent peripheral blood collection for the analysis of genetic polymorphisms, serum cytokines and DAT biomarkers, and, in some cases, CSF collection for DAT biomarkers and cytokine analysis.

To align with evolving diagnostic standards, the Cog‐Aging study has periodically updated its cohort classifications. Initially defined in 2010, cognitively unimpaired (CU) individuals were characterized by an absence of subjective memory complaints (reported by the participant or informant), preserved performance on objective cognitive assessments, intact Activities of Daily Living (ADLs), and a Clinical Dementia Rating (CDR) score of 0.^8^, ^9^ This definition was updated in 2014 following the publication of standardized diagnostic criteria for subjective cognitive decline (SCD) and CU status.^10^, ^11^ Similarly, diagnoses of MCI followed Petersen's criteria until 2014,^12^ after which the study adopted the *Diagnostic and Statistical Manual of Mental Disorders*, Fifth Edition (DSM‐5) framework.^13^ Dementia diagnoses were established according to DSM‐IV criteria until 2014^14^ and according to DSM‐5 thereafter.^13^ Regarding DAT, the diagnostic protocol transitioned from DSM‐IV^14^ to the McKhann et al. criteria for probable DAT.^15^ Most recently, these clinical guidelines have been supplemented by the revised criteria for diagnosis and staging of Alzheimer's disease,^16^ facilitating the integration of biomarker data when available. All participants in the Cog‐Aging cohort underwent a standardized protocol of clinical, cognitive, and functional assessments. The Cog‐Aging outpatient extension service was established in 2010 and used a standardized clinical and cognitive assessment protocol grounded in established best practices for clinical and neuropsychological evaluation. In 2011, this pre‐existing clinical assessment protocol was subsequently incorporated as the standardized assessment protocol of the formal research cohort. Standardized clinical assessments performed through the outpatient service between 2010 and 2011 were incorporated as pre‐existing baseline data. Their inclusion was conducted in accordance with the ethics‐approved protocol and informed‐consent procedures governing the use of collected clinical information. To ensure diagnostic consistency across recruitment periods, diagnoses of Alzheimer's disease dementia, including those based on assessments conducted before formal approval of the research protocol, were retrospectively harmonized using the criteria published by McKhann et al.^15^ Although international diagnostic criteria evolved over the follow‐up period, the core clinical variables needed for diagnosis were systematically collected throughout all phases. These include subjective cognitive complaints, objective performance on a comprehensive neuropsychological assessment, functional status, and multidisciplinary consensus meetings.

To maintain longitudinal consistency, diagnoses were retrospectively harmonized to align with contemporary frameworks. This process relied entirely on clinical data recorded at the time of the original assessments, avoiding the need to reconstruct diagnoses from incomplete records. The correspondence between the evolving diagnostic frameworks and the variables systematically collected in our cohort is detailed in Table S2.

Finally, although SCD was formally introduced into diagnostic frameworks after the cohort was established, it was not analyzed as a separate diagnostic category in this study. Individuals with SCD were grouped with the CU reference group. As a result, changes in SCD nomenclature did not affect the cohort's analytic structure or the primary outcomes.

Since the baseline evaluation, diagnoses were consistently established via a comprehensive consensus process integrating neuropsychological performance, functional status, and clinical history to contextualize scores for each participant's age, education, and clinical background. While the employed neuropsychological tests are standardized for our demographic, we additionally developed a tailored protocol with cohort‐specific cutoff values to ensure accurate assessment of older adults with low education.^17^ Supplementary material details the classification thresholds for functional and cognitive impairment (Table S3, Appendix 1).

#### 2.3.2 Geriatric assessment

The geriatric assessment protocol consists of the following: (1) a sociodemographic questionnaire; (2) an evaluation of personal and family clinical history; and (3) characterization of comorbidities: arterial hypertension (AH), diabetes mellitus (DM), dyslipidemia, coronary artery disease (CAD), heart failure, stroke, atrial fibrillation (AF), cancer, chronic kidney disease, liver disease, neurodegenerative diseases (Parkinson's disease and dementia), presence of other mental health disorders (major depressive disorder, anxiety disorders, bipolar disorder), medications in use, lifestyle habits (smoking and alcohol consumption), and details of forgetfulness complaints. Data are collected from participants' and study partners' records and medical reports.

Study partners are well characterized as either professionals or family members who spend most of their time and most weekdays with the participants (age, degree of kinship, educational level, time of contact with the patient, cognitive ability).^18^ The data evaluated at this stage of the research included sex, age, education, study partner characterization, lifestyle habits, and clinical comorbidities.

Laboratory tests and neuroimaging (computed tomography and/or magnetic resonance imaging [MRI]) are performed to support the diagnosis and to verify the research exclusion criteria. Besides the neuroimaging signs of DAT, individuals with white matter hyperintensities at T2 MRI sequences and/or other cerebrovascular lesions whose changes are insufficient to explain cognitive decline are included.

A clinical assessment of older adults is conducted by a standardized protocol emphasizing postural hypotension, postural instability, gait, signs of sarcopenia,^19^ and anthropometry^20^, ^21^, ^22^ to prevent underestimating diagnoses.

After the assessments, the cases are discussed with the geriatricians and geriatric psychiatrists’ study coordinators, both of whom are duly certified, trained, and experienced in their fields.

#### 2.3.3 Cognitive assessment

A brief cognitive screening is a component of the geriatric assessment, whereas the most extensive cognitive evaluation is done by neuropsychologists. Both protocols include validated and standardized instruments adapted to the population served. Over the years, through longitudinal assessments of older adults with low levels of education, a specific neuropsychological protocol was developed and corresponding cutoff values established to ensure adequate assessment of this population.^17^ Recently, this protocol was revised; however, to preserve construct stability and longitudinal measurement invariance, all analyses of cognitive trajectories and time‐to‐event outcomes were restricted to instruments and procedures that remained unchanged throughout the study (Figure S2). For these measures, administration protocols, scoring criteria, and test versions were consistent from baseline through follow‐up assessments. Supplementary material details the current protocol (Table S4).

#### 2.3.4 Neuropsychological assessment

The neuropsychological assessment takes place in two sessions lasting 70 min each. The cognitive assessment protocol includes a series of tests that assess various cognitive domains (Table S4).

The Reliable Change Index (RCI) is applied to compare each participant's performance across consecutive cognitive assessments. This approach provides insight into individual changes relative to their own previous results, rather than relying on normative group comparisons.^23^

#### 2.3.5 Ecological‐functional assessment

Functional assessment includes a variety of application methods.^24^, ^25^, ^26^ In the Cog‐Aging study, the first step consists of an interview with the caregiver‐older person dyad. The caregiver must be over 18 years old and be familiar with the participant's daily routine.^16^, ^24^, ^27^

Following the interview, objective or ecological standardized instruments are applied to measure the older adult's current and habitual performance in ADLs.^24^, ^25^, ^26^, ^28^ Among the instruments used, all adapted to Brazilian culture and with consolidated psychometric properties, the following stand out:

- The Canadian Occupational Performance Measure (COPM), which characterizes routine and identifies compromised ADLs, enables the evaluation of performance and satisfaction in the areas of self‐care, productivity, and leisure.^29^, ^30^
- The Functional Independence Measure (FIM), which assesses performance in the motor and cognitive/social domains through observation of the activities performed.^31^
- The Direct Assessment of Functional Status (DAFS) investigates the performance of older adults with MCI or dementia in ADLs that require cognitive skills, such as temporal orientation, communication, money management, shopping, dressing/personal hygiene, and eating.^32^, ^33^, ^34^

The ecological functional assessment is conducted in an adapted office by an occupational therapist with training in gerontology and extensive experience in elderly care. It lasts approximately 60 min.

#### 2.3.6 Nutritional assessment

The nutritional assessment comprises two stages: dietary intake assessment, anthropometric and body composition evaluation, and tests for sarcopenia assessment. In the first stage, a food frequency questionnaire validated for the Brazilian population is administered,^35^ which also includes foods typically consumed in the study region. This questionnaire evaluates the frequency of consumption of 144 foods from various groups, including breads, cereals, and tubers; fruits; vegetables, greens, and legumes; eggs, meats, milk, and dairy products; pasta and other preparations; sweets; and alcoholic and non‐alcoholic beverages. In addition to the questionnaire, we include questions regarding the consumption of ultra‐processed foods and the daily intake of sugar, oil, fat, and salt.

In the second stage, anthropometric measurements include weight, height, waist, hip, and calf circumferences.^36^ Body composition is assessed using bioelectrical impedance analysis (BIA) with a single‐frequency (50 kHz) tetrapolar Biodynamics 310 device and by dual‐energy X‐ray absorptiometry (DXA) using the GE Lunar Prodigy, which is scheduled at the end of the evaluation. Sarcopenia is screened through muscle strength (handgrip strength and the chair stand test) and physical performance assessments (gait speed and the timed up and go test).^37^

The full assessment lasts approximately 120 min and is conducted by a nutritionist specializing in gerontology. Follow‐up evaluation occurs annually, and participants receive tailored dietary guidance aligned with the Brazilian Dietary Guidelines for older adults.^38^

#### 2.3.7 Speech‐language assessment

Participants assessed by the speech‐language pathology team undergo a comprehensive assessment subject to a protocol intended to evaluate hearing, cognition, and balance using validated assessment instruments. The evaluation begins with a detailed medical history, collecting information on medication use, family history, and symptoms such as dizziness, reduced hearing acuity, and tinnitus. The hearing assessment comprises inspection of the external auditory canal followed by tympanometry, pure‐tone audiometry, and speech audiometry. Cognitive aspects related to auditory processing are examined using the long‐latency auditory evoked potential (P300).

Balance assessment includes static post‐urography integrated with dynamic tests, alongside the administration of the Berg Balance Scale and the Falls Efficacy Scale–International (FES‐I). At the end of the evaluation, patients receive individualized feedback and recommendations based on their specific findings. When hearing loss is identified, the patient is informed about the condition and, if indicated, referred to the Hearing Health Program of the Brazilian Unified Health System (SUS).

#### 2.3.8 Consensus meetings

Diagnoses are established through a multidisciplinary consensus process conducted in periodical meetings. Throughout the study, the panel has comprised a geriatrician, a geriatric psychiatrist, and a neuropsychologist, with an occupational therapist joining in later years. From the baseline evaluation onward, diagnoses integrates neuropsychological performance, functional status, and clinical history, contextualizing each participant's scores according to age, education, and clinical background. The principal investigator reviews each participant's diagnosis annually.

To prevent ascertainment bias and align with current clinical‐biological frameworks, the diagnostic process follows a strict two‐step structure. First, the panel determines the clinical syndrome (cognitively unimpaired, MCI, or dementia) based solely on the clinical, cognitive, and functional assessment described earlier. At this stage, laboratory tests and structural neuroimaging are reviewed only to exclude reversible and secondary causes of cognitive impairment, and the panel remains blinded to fluid biomarkers and APOE ε4 (ε4 allele of apolipoprotein 4 gene) genotypes. This syndromic classification constitutes the clinical reference standard used throughout the present study, including for the longitudinal endpoint of conversion to all‐cause dementia. Only in a second step does the panel integrate each participant's available supportive data, when available, which could include neuroimaging, fluid biomarkers, and APOE genotype to establish the underlying etiology (e.g., DAT).

#### 2.3.9 Maintenance, retention, and dropout management

Loss to follow‐up is a common occurrence in longitudinal cohort studies^39^ and may affect both participants and their legal representatives.

**FIGURE 1:**
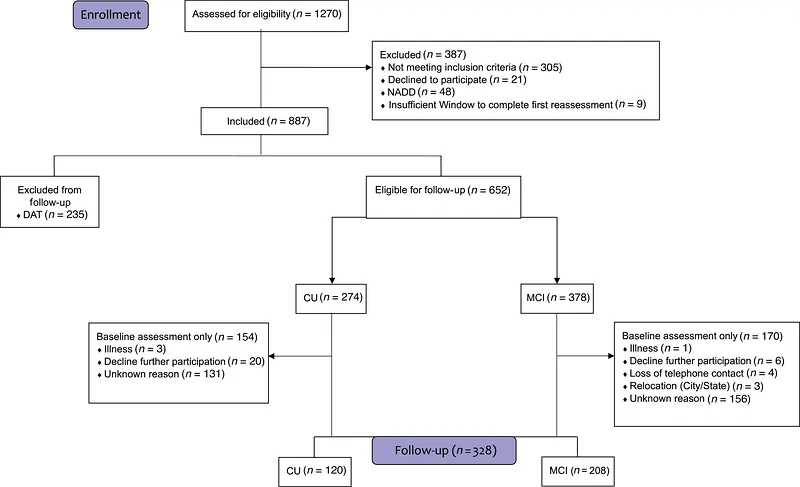
Study sample flow diagram. Cognitively unimpaired (CU); dementia of the Alzheimer's type (DAT); mild cognitive impairment (MCI); non‐Alzheimer's disease dementia (NADD).

In Brazil, current legislation^40^, ^41^ prohibits the use of financial incentives for participation in scientific research; therefore, participants must be volunteers. However, the law ensures that participants do not incur costs due to involvement in research. Moreover, it is necessary to consider that, in LMICs; maintaining a telephone number represents an economic challenge, leading to frequent changes in participant contact information. Furthermore, during the COVID‐19 pandemic, telephone scams increased in Brazil, leading many participants to avoid answering calls from unknown numbers. As a result, difficulties in establishing telephone contact accounted for a substantial proportion of the loss to follow‐up. There's also an important component in LMICs whereby participants are lost due to a lack of accessibility on the way to the center and frequent instances of participants repeatedly confirming their appointments but failing to attend. In addition, during the initial phases of the study, operational and human resource constraints limited our ability to implement active retention and attrition management strategies. The reasons for loss to follow‐up in the Cog‐Aging study are summarized in Figure 1.

In the Cog‐Aging study, we adopted the measures reviewed by Teague et al.^39^ and proposed by the frameworks of the cohort maintenance report from the Irish Longitudinal Study on Aging (TILDA),^42^ focusing on reminders, tracking, maintaining continuity of interviewer‐participant relationship, and community building. Additionally, we introduced a fifth domain, minimizing participant burden, to avoid attrition related to representatives.^39^ Since the COVID‐19 pandemic, we have further strengthened and refined our communication strategies with both participants and their families. Moreover, as the cohort has increased and the research team expanded, we have incorporated more structured approaches to monitoring participant loss and sustaining engagement. These include centralizing study communications through a single, dedicated telephone number and utilizing WhatsApp instant messaging, visually identified with the research group's official logo to establish trust. Supplementary material highlights the primary strategies employed by Cog‐Aging and details the specific actions implemented for each to minimize loss of follow‐up (Table S5).

To support these efforts, research coordinators now maintain close communication with health centers and referral teams, complemented by direct telephone contact with participants or their representatives. These interactions enable us to identify specific reasons for dropouts and to adjust procedures to facilitate continued follow‐up.

#### 2.3.10 Neuroimaging

All participants with cognitive complaints underwent identical clinical and neuropsychological evaluations, as well as laboratory testing. Those who presented cognitive impairment were additionally invited to undergo neuroimaging examinations. At the beginning of this study, computed tomography was the most commonly available neuroimaging method, and some patients were assessed by fluorodeoxyglucose positron emission tomography. However, currently, these participants are submitted to MRI. This has made it possible to categorize atrophies and cerebrovascular alterations according to standard visual scales: Global Cortical Atrophy,^43^ visual scales for the evaluation of medial temporal^44^ and entorhinal atrophy,^45^ scale for evaluation of white matter lesions^46^ and posterior cortical atrophy. The MRI scans are blindly evaluated by two independent physicians using these visual scales. Moreover, in 2025, a partnership was established with the Hermes Pardini‐Fleury Institute, allowing participants who have undergone the complete protocol to have a 3T MRI including the Alzheimer's Disease Neuroimaging Initiative (ADNI) 3.0 protocol.^47^

#### 2.3.11 Biological sample collection and laboratory procedures

Peripheral blood is collected in EDTA tubes during the geriatric assessment, after informed consent is obtained. The samples are then centrifuged at 4°C, 3000 × g for 15 min and stored at −80°C or submitted to DNA extraction from peripheral blood leukocytes using the saline method established by Lahiri and Nurnberger,^48^ modified by Cavalli et al.^49^ and Salazar et al.^50^ Samples are collected and stored annually, ensuring at least one baseline sample per participant. To date, out of the 463 participants who have at least one sample stored, 299 plasma samples have been analyzed.

After clinical assessments, participants with MCI and those diagnosed with dementia, and without contraindications for the procedure, are invited to undergo CSF collection via lumbar puncture. After collection, the samples are promptly sent to the laboratory and centrifuged at 4°C for 10 min at 3000 × g. The biological material is then stored at −80°C. Between 2011 and 2025, a total of 125 CSF samples were collected and 100 samples analyzed.

Genotyping analysis of *APOE* polymorphisms is performed using the real‐time PCR technique^51^ in the laboratory of Molecular Medicine of the Faculty of Medicine of the UFMG. Also, single‐nucleotide polymorphisms (SNPs) were genotyped using the Illumina Infinium Global Screening Array (GSA) version 1.0 with shared custom content and GSA‐24 version 3.0. Genome‐wide association studies are performed in collaboration with Professor Alfredo Ramirez Zuniga at the University of Cologne, Germany.

CSF is analyzed to determine amyloid beta (Aβ) 42, phosphorylated tau (p‐tau), and total tau (t‐tau) levels using the Luminex xMap technique. We performed experiments using the Human Amyloid Beta and Tau Magnetic Bead Panel kit (HNABTMAG‐68K) (Millipore, Germany). Additionally, cytokine levels in CSF and plasma were also determined by the Luminex xMap technique using Bio‐Plex Pro Human Cytokine 27‐plex Assay No. M500KCAF0Y from BIO‐RAD, which allowed for examination of the following cytokines: IL‐1β, IL‐1ra, IL‐2, IL‐4, IL‐5, IL‐6, IL‐7, IL‐8, IL‐9, IL‐10, IL‐12, IL‐13, IL‐15, IL‐17, Eotaxin, FGF, G‐CSF, GM‐CSF, IFNγ, IP‐10, MCP‐1, MIP‐1α, MIP1β, PDGF‐BB, RANTES, TNF‐alpha, VEGF.

Since 2023, the data sample has been stored on the REDCap platform.

Over the course of the study, the assessment protocol evolved to incorporate advances in neuropsychology methodology, biomarker technologies, and neuroimaging acquisition standards, in alignment with emerging scientific evidence and updated international diagnostic frameworks. These updates were implemented to maintain scientific relevance and methodological rigor rather than to modify the underlying cognitive constructions being assessed.

We classified participants not only based on clinical assessment but also following the AT(N) system.^52^ We consider the levels of Aβ42, p‐tau, and t‐tau in the CSF obtained by the Luminex xMap technique. More recently, we performed a data‐driven cutoff point established by Ramirez et al.^53^ A preliminary report on positivity to CSF Aβ, plasma p‐tau217, and APOE ε4 carrier status at baseline considering this cutoff is described in the supplementary material (Table S6).

Initially, CSF biomarkers (Aβ40, Aβ42, p‐tau, t‐tau) were quantified using ELISA. Once the cutoff points were established via ELISA, the analyses were repeated using Luminex technology to ensure the reliability of these CSF markers (including the Aβ40/Aβ42 ratio) and the aforementioned cytokines. The most recent advancement in the incorporation of new technologies has been the assessment of plasma Aβ40 and Aβ42 using the Neurology 4‐Plex E Simoa assay kit (Quanterix, Billerica, MA, USA) and p‐tau217 using the ALZpath V2 Simoa assay kit (Quanterix, Billerica, MA, USA) on a Simoa HD‐X platform at the DZNE, Bonn, Germany.

### 2.4 Statistical analysis

Participant characteristics were described for the overall population and compared between participants classified as CU and MCI at baseline. Continuous variables were summarized as mean and standard deviation and compared using parametric or non‐parametric tests. Categorical variables were presented as counts and percentages and compared using chi‐squared or Fisher's exact tests, as appropriate. Data availability for each variable within each group was also reported as a supplementary analysis.

Analyses were conducted to estimate progression from MCI to dementia and progression from CU to dementia. For each outcome, analyses were performed on separate datasets including only individuals at risk for the specific outcome of interest.

First, annualized conversion rates to dementia were estimated as incidence densities per 100 person‐years. Person‐time at risk was calculated from the baseline clinical evaluation to the date of conversion to dementia, death, last clinical assessment, or end of follow‐up, whichever occurred first. Participants who did not convert during follow‐up contributed person‐years until censoring. Conversion rates were computed overall and stratified by sex, age group (60 to 69, 70 to 79, and ≥80 years), and education level (0, 1 to 4, 5 to 8, and >8 years), based on the distribution of schooling in the cohort and prior literature from LMIC settings.^54^ Exact 95% confidence intervals (CIs) were derived assuming a Poisson distribution of events. These analyses were restricted to individuals with MCI, as the CU group presented a limited number of dementia conversions, which could result in unstable estimates and potential bias, particularly in stratified analyses. To account for the competing risk of death and to complement the annualized conversion rates, cumulative incidence functions (CIFs) were estimated to describe the occurrence of dementia over follow‐up in the presence of competing events. This approach is particularly relevant in the CU group, where the small number of events may lead to unstable incidence estimates when competing risks are not properly considered. Time to event was defined from baseline clinical assessment to the occurrence of dementia, death, or censoring at the last follow‐up visit. CIF was estimated non‐parametrically within a competing‐risk framework, and point estimates with corresponding 95% CIs were obtained at prespecified follow‐up time points. Stratified cumulative incidence analyses were performed only in the MCI group, given the limited number of conversion events in the CU group, which precluded reliable subgroup estimation. CIF was stratified according to sociodemographic variables, including age, sex, and years of education, to explore differences in conversion risk across subgroups.

To further investigate the association between sociodemographic and clinical variables and conversion to dementia while accounting for the competing risk of death, we conducted univariable and multivariable Fine–Gray subdistribution hazard regression analyses. The baseline variables (age, sex, years of education hypertension, diabetes, dyslipidemia, depression, hearing loss, smoking, alcohol abuse, visual impairment, stroke, coronary artery disease, and obesity) were first evaluated in univariable Fine–Gray subdistribution hazard models, with one predictor entered at a time. Age, sex, and education were retained in the multivariable models regardless of their univariable *p* values due to their well‐established association with cognitive decline and dementia risk. Education was categorized using the same groups applied in the annualized conversion rate analyses and in the subgroup assessment of cumulative incidence functions: 0, 1 to 4, 5 to 8, and >8 years of education. In both univariable and multivariable Fine–Gray competing‐risk regression models, the 0‐year education group was used as the reference category, as illiteracy/no formal education has been associated in the literature with the highest risk of cognitive decline and dementia.

As an exploratory analysis, APOE ε4 carrier status was added as a binary covariate to the multivariable Fine–Gray model. Missing APOE ε4 carrier status was addressed using substantive‐model‐compatible fully conditional specification adapted for Fine–Gray competing‐risk regression. The substantive model included age, sex, education, and APOE ε4 carrier status, whereas follow‐up time, dementia event status, and competing death status was incorporated through the Fine–Gray‐compatible imputation procedure. Depression, recruitment year, family history of dementia, dyslipidemia, obesity, arterial hypertension, and DM were included as auxiliary variables. Convergence and imputation diagnostics were assessed; the distribution of imputed APOE ε4 carrier status was examined for plausibility, and estimates from the imputed Fine–Gray models were combined using Rubin's rules. Complete‐case and multiple‐imputation results were reported as supplementary exploratory analyses.

To evaluate the potential impact of attrition due to baseline‐only participation, we performed a sensitivity analysis that incorporated participants who completed the baseline clinical evaluation but did not undergo any subsequent longitudinal assessment. These participants were not included in the primary analysis because they did not contribute observed follow‐up time for dementia conversion. For the sensitivity analysis, they were treated as censored at baseline within the censoring model, and inverse probability of censoring weighting (IPCW) was used to account for differences in the probability of remaining under follow‐up. The IPCW sensitivity analysis was applied to annualized conversion rates, estimated as incidence densities per 100 person‐years; cumulative incidence functions for dementia conversion, with death treated as a competing risk; and univariable and multivariable Fine–Gray subdistribution hazard models.

As an additional sensitivity analysis, we expanded the IPCW censoring model to include depression, hearing loss, and alcohol abuse. These variables were selected because they differed between participants with baseline‐only assessments and those with longitudinal follow‐up, suggesting that they could be associated with the probability of remaining under observation. The expanded IPCW model was therefore used to assess whether further adjustment for these potential predictors of follow‐up participation affected the annualized conversion rates, CIFs, and Fine–Gray model estimates. Because missing data in these covariates reduced the available complete‐case sample, this analysis was considered complementary to the primary IPCW analysis, rather than its replacement. The methodological details of both sensitivity analyses are described in Appendix 2.

Considering the longitudinal nature of the study and the possibility that changes over time may have influenced cohort composition and diagnostic ascertainment, a recruitment‐era sensitivity analysis was conducted in the MCI group to assess potential temporal heterogeneity. Recruitment era was added as a categorical covariate to the multivariable Fine–Gray model including age, sex, and educational category, with death treated as a competing event. Participants were categorized into three recruitment periods: enrollment up to 2014, enrollment from 2015 through 2019, and enrollment from 2020 onward. The 2011 to 2014 era was chosen because it marked the incorporation of the McKhann criteria into the diagnostic framework for DAT, plus DSM‐IV for other dementias and Petersen's Criteria for MCI. For the period from 2015 to 2019, the DSM‐5 was incorporated as the standard for other dementias (major neurocognitive disorder) and MCI (mild neurocognitive disorder). The 2020‐onward period was selected because it coincided with relevant changes: the incorporation of fluid biomarkers for Alzheimer's disease, the introduction of the Informant Questionnaire on Cognitive Decline in the Elderly (IQCODE) (Table S2) instrument into the assessment protocol, and changes due to the COVID‐19 pandemic. Details of this sensitivity analysis are described in the supplementary material.

To assess the robustness of the multivariable Fine–Gray competing‐risk models, we conducted additional diagnostic analyses evaluating model stability, sparse‐data patterns, proportional subdistribution hazard assumptions, and potential influence of individual observations. Methodological details are provided in Appendix 2.

All statistical analyses were conducted using R version 4.6.0 (R Foundation for Statistical Computing, Vienna, Austria) and the RStudio integrated development environment version 2026.04.0 (Posit, Public Benefit Corporation, Boston, MA, USA). The following R packages were used: dplyr, tidyr, stringr, readr, magrittr, forcats, survival, cmprsk, riskRegression, epitools, ggplot2, patchwork, scales, gtsummary, gt, flextable, broom, writexl, openxlsx, purrr, tibble, rlang, and mice.

## 3 RESULTS

Between August 2011 and June 2025, 1270 older adults were eligible for the Cog‐Aging study. After excluding 383 individuals, 887 fulfilled inclusion criteria and were separated into three groups according to the first clinical cognitive diagnosis: CU (274), MCI (378), and DAT (235). We followed up with 120 participants of the CU group and 208 of the MCI group (Figure 1). Mean age at baseline was 75.28 years (SD 7.26), 69.6% were female, and mean education was 5.45 years (SD 4.7). The most prevalent comorbidities were hypertension (71.3%), dyslipidemia (47.1%), and DM (30.8%). When comparing diagnostic groups, age and education differed across CU, MCI, and DAT (*p* < 0.001), with the DAT group being older and having lower educational attainment than the CU and MCI groups. A sex distribution difference was also identified between groups (*p* = 0.032), with women being more prevalent in the CU group. Obesity (*p* = 0.007) was more frequent in CU group, and stroke history (*p* = 0.005) was more frequent in DAT. APOE ε4 carrier status showed a gradient across groups (*p* < 0.001) that was lowest in CU and highest in DAT. In contrast, most comorbidities and lifestyle factors did not significantly differ across groups. A detailed description of the overall population and stratified diagnostic groups is presented in Table 1 and supplementary material (Table S7).

**TABLE 1:** Sociodemographic and clinical characteristics to cog‐aging cohort.

Characteristic	Overall *N* = 887	CU *N* = 274	MCI *N* = 378	DAT *N* = 235	*P* value^*^
**Education**	5.45 (4.7)	7.21 (5.23)	4.69 (4.21)	4.59 (4.25)	<0.001^†^
**Age**	75.28 (7.26)	72.62 (7.17)	75.49 (7.17)	78.03 (6.4)	<0.001^†^

Sex					
Female	617 (69.6%)	207 (75.5%)	255 (67.5%)	155 (66.0%)	0.032^†^
Male	270 (30.4%)	67 (24.5%)	123 (32.5%)	80 (34.0%)	
**Hypertension**	554 (71.3%)	163 (70.3%)	247 (71.4%)	144 (72.4%)	0.890
**Diabetes mellitus**	238 (30.8%)	65 (28.1%)	115 (33.5%)	58 (29.3%)	0.337
**Dyslipidemia**	286 (47.1%)	97 (46.9%)	127 (48.1%)	62 (45.6%)	0.888
**Depression**	326 (45.0%)	79 (38.3%)	159 (48.3%)	88 (46.6%)	0.069
**Hearing loss**	177 (29.2%)	47 (31.5%)	78 (28.5%)	52 (28.4%)	0.770
**Obesity**	116 (22.9%)	53 (28.6%)	51 (22.9%)	12 (12.1%)	0.007^†^
**Smoking**	155 (30.8%)	52 (34.7%)	66 (30.1%)	37 (27.6%)	0.420
**Alcohol abuse**	188 (37.5%)	63 (42.3%)	83 (37.9%)	42 (31.6%)	0.178
**Visual impairment**	219 (36.4%)	59 (40.7%)	98 (35.5%)	62 (34.4%)	0.462
**Stroke**	63 (8.6%)	12 (6.0%)	24 (7.0%)	27 (14.3%)	0.005^†^
**Coronary artery disease**	55 (8.4%)	13 (7.8%)	25 (8.3%)	17 (9.0%)	0.917
**Atrial fibrillation**	19 (4.4%)	7 (5.6%)	9 (4.7%)	3 (2.6%)	0.503
**ApoE ε4 carrier**	179 (36.8%)	31 (19.7%)	86 (40.2%)	62 (53.9%)	<0.001^†^

Abbreviations: CU, cognitively unimpaired; DAT, dementia of the Alzheimer's type; MCI, mild cognitive impairment.

* *P* value considered for chi‐squared, Fisher's exact, Mann‐Whitney, Student's *t*‐test as appropriate for each variable.

^†^ *P* < 0.05.

In the longitudinal CU‐to‐dementia analysis, 120 participants were included for follow‐up analysis. During follow‐up, 99 (82.5%) remained alive and did not convert to dementia, 15 (12.5%) converted to dementia, and six (5%) died. Follow‐up time ranged from 0.5 to 13.6 years, with a mean of 5.02 years (SD: 3.88). In the MCI‐to‐dementia group, 208 participants were included at baseline. During follow‐up, 110 (52.88%) remained alive and did not convert to dementia, 84 (40.39%) progressed to dementia, and 14 (6.73%) died. Follow‐up time ranged from 6 months to 15 years, with a mean of 3.75 years (SD: 3.28). Baseline comparisons between participants retained in longitudinal follow‐up and those with baseline‐only assessments are presented in Table S8. In the CU group, participants with baseline‐only assessments differed from those retained in follow‐up in depression (*p* = 0.010) and hearing loss (*p* = 0.014). In the MCI group, baseline differences were observed for alcohol abuse (*p* = 0.023) and hearing loss (*p* = 0.015). No other baseline characteristics differed significantly between groups.

Annualized conversion rates were calculated only for the MCI group, as the number of conversion events in the CU group was too small to provide stable estimates, particularly in stratified analyses. The overall annualized conversion rate from MCI to dementia was 10.72 per 100 person‐years (95% CI: 8.54 to 13.29), based on 83 events over 774.1 person‐years of follow‐up.

**FIGURE 2:**
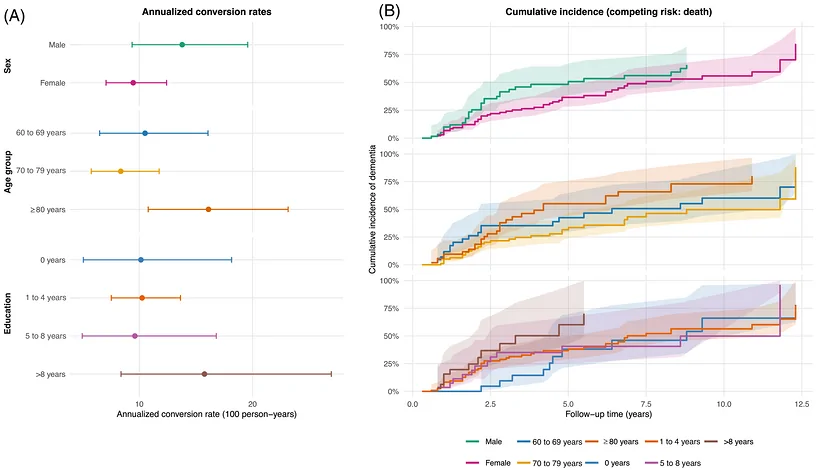
Annual MCI‐to‐dementia conversion rates and cumulative incidence functions, stratified by sex, age group, and education. *Cumulative incidence of dementia and annualized conversion rates according to sex, age group, and education level. The left panels display cumulative incidence functions (CIFs) for conversion to dementia, accounting for the competing risk of death, with follow‐up truncated at 10 years to improve interpretability and reduce instability at later time points. The right panels show annualized conversion rates per 100 person‐years with 95% Poisson confidence intervals. Age groups were defined as 60 to 69, 70 to 79, and ≥80 years, and education categories as 0, 1 to 4, 5 to 8, and >8 years. These complementary measures provide both absolute risks over time and incidence density estimates across clinically relevant subgroups.

When stratified by sex, men showed a higher conversion rate than women (13.79 vs 9.47 per 100 person‐years, respectively). Across age groups, participants aged ≥80 years exhibited the highest rate (16.1 per 100 person‐years), followed by those aged 60 to 69 years (10.5 per 100 person‐years) and 70 to 79 years (8.3 per 100 person‐years). Across education strata, conversion rates were relatively similar among individuals with 0 to 8 years of formal education, ranging from 9.62 to 10.25 per 100 person‐years. However, participants with more than 8 years of education showed a higher annualized rate of 15.76 per 100 person‐years, although confidence intervals were wide (Figure 2, Figure S3, and Table S9).

**FIGURE 3:**
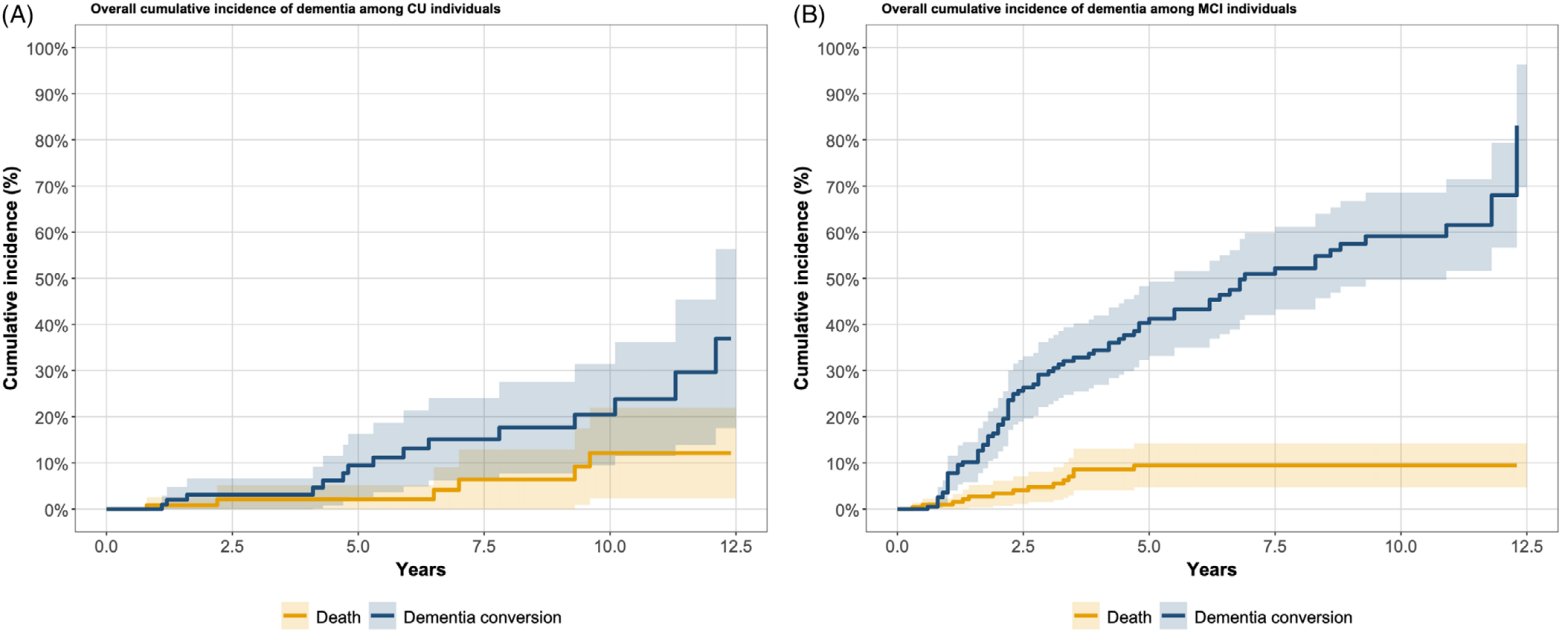
Cumulative incidence between cognitively unimpaired and MCI. *The cumulative incidence of the event of interest was estimated using non‐parametric cumulative incidence functions (CIFs), accounting for death as a competing event. Time to event was measured in years from baseline to the occurrence of the event of interest, competing event (death), or censoring. The CIF was plotted up to 12 years of follow‐up. Separate curves were displayed for the event of interest and for death as a competing event. Pointwise 95% confidence intervals were calculated using the variance estimates provided by the CIF, assuming asymptotic normality. Confidence bands were displayed as shaded areas around the step‐function curves. The cumulative incidence was expressed as percentage risk over time. To enhance interpretability, vertical reference lines were drawn at predefined follow‐up milestones (0, 3, 6, 9, and 12 years), and horizontal reference lines were displayed at 10% increments of cumulative incidence.

The cumulative incidence of conversion from CU to dementia was low during the early years of follow‐up but increased gradually over time. Accounting for death as a competing event, the estimated cumulative incidence of dementia was 0.00% at 1 year (95% CI: 0.00 to 0.00), 3.12% at 3 years (95% CI: 0.00 to 6.60), 9.42% at 5 years (95% CI: 2.62 to 16.23), and 20.44% at 10 years (95% CI: 9.43 to 31.46%). In the MCI group, the estimated cumulative incidence was 7.78% at 1 year (95% CI: 4.00 to 11.57%), 29.85% at 3 years (95% CI: 22.74 to 36.96%), 41.24% at 5 years (95% CI: 33.15 to 49.33%), and 59.13% at 10 years (95% CI: 49.68 to 68.58%) (Figure 3, Table S10). Confidence intervals became wider at later follow‐up time points, particularly at 10 years, indicating greater statistical uncertainty in long‐term cumulative incidence estimates, likely reflecting the smaller number of participants remaining under observation over time.

CIFs stratified by sex, age, and education were estimated only in the MCI group, given the limited number of conversion events in the CU group, which precluded reliable subgroup estimation. Age‐stratified CIFs showed that individuals aged ≥80 years exhibited the highest probability of dementia over follow‐up, reaching 40.55% at 3 years, followed by those aged 60 to 69 years (35.29%) and 70 to 79 years (21.67%). The apparently lower cumulative incidence of dementia in the 70‐ to 79‐year group may reflect the distribution of competing events across age strata. No deaths were observed among participants aged 60 to 69 years, whereas nine deaths occurred among those aged 70 to 79 years. Because death was treated as a competing event, this difference may have reduced the observed cumulative incidence of dementia in the 70‐ to 79‐year group. At 3 years of follow‐up, the estimated probability of dementia was 43.1% in those with >8 years of education, compared with 9.53% in individuals with no formal education and 30.06% in those with 1 to 4 years. Participants with 5 to 8 years of education showed intermediate estimates (35.07%). The higher cumulative incidence of dementia in the >8‐year education group should be interpreted cautiously, as competing mortality differed across educational strata. At 3 years, no deaths occurred in this group, compared with 18.5% among participants with no formal education, 6.9% among those with 1 to 4 years, and 3.3% among those with 5 to 8 years of education. As death was treated as a competing event, differences in mortality may have influenced the subgroup‐specific cumulative incidence estimates. The cumulative incidence of conversion from MCI to dementia was higher in men. At 3 years of follow‐up, the estimated probability of dementia was 41.48% in men compared with 24.13% in women. However, death was also higher among men, reaching 9.52% compared with 5.59% in women (Figure 2, Table S11).

In univariable Fine–Gray competing‐risk models assessing conversion from CU to dementia, older age was significantly associated with a higher subdistribution hazard of dementia conversion (subdistribution hazard ratio [SHR] = of 1.12 (95% CI: 1.03 to 1.22; *p* = 0.011), as was atrial fibrillation (SHR = 18.30; 95% CI: 1.183 to 283.112; *p* = 0.038). Using participants with no formal education as the reference group, none of the other educational categories was significantly associated with dementia conversion. Likewise, sex, hypertension, diabetes, dyslipidemia, depression, hearing loss, smoking, alcohol abuse, visual impairment, stroke, coronary artery disease, and obesity were not significantly associated with dementia conversion (Table S12).

In the MCI group, neither age nor sex was significantly associated with the subdistribution hazard of dementia conversion. Compared with participants with no formal education, no educational category showed a statistically significant association with dementia conversion, except for those with more than 8 years of education, who exhibited a higher subdistribution hazard of dementia conversion (SHR = 2.11; 95% CI: 1.031 to 4.404; *p* = 0.046) (Table S12).

In the CU group, multivariable Fine–Gray competing‐risk models adjusted for age, sex, and education showed that age remained significantly associated with the subdistribution hazard of conversion to dementia (SHR = 1.141; 95% CI: 1.013 to 1.285; *p* < 0.030). Sex and educational attainment were not significantly associated with conversion in the adjusted model (Table S13). However, robustness diagnostics indicated limited stability of the adjusted Fine–Gray model (Appendix 2). Therefore, adjusted Fine–Gray estimates in the CU cohort should be interpreted as exploratory and hypothesis‐generating (Table S13).

In the MCI group, age and sex were not associated with a higher subdistribution hazard in the adjusted model. Only participants with >8 years of education showed a statistically significant higher subdistribution hazard compared with those with no formal education (SHR = 2.279; 95% CI: 1.021 to 5.088; *p* = 0.044; Table 2). However, given the borderline statistical significance and wide CI, this finding should be interpreted cautiously (Table 2). In robustness diagnostics, no sparse‐data warnings were observed across sex, education categories, or age quartiles, and no model terms showed numerical instability. Tests based on interactions with log‐transformed follow‐up time did not indicate violation of the proportional subdistribution hazard assumption. Although leave‐one‐out analyses identified influence‐related warnings for all model terms, all refitted models converged, and median coefficient changes were small, suggesting that the overall model interpretation was stable (Appendix 2).

**TABLE 2:** Fine–Gray multivariable model for MCI group considering sex, age, and education, with IPCW‐weighted sensitivity analysis.

Predictor				
*MCI*	Dementia events	Competing deaths	*P* value	SHR (95% CI)
Age	83	14	0.081	1.034 (0.996 to 1.074)
Sex	83	14	0.301	0.785 (0.496 to 1.243)
Education (1 to 4 years)	83	14	0.434	1.254 (0.71 to 2.215)
Education (5 to 8 years)	83	14	0.432	1.365 (0.628 to 2.968)
Education (>8 years)	83	14	0.044^*^	2.279 (1.021 to 5.088)

Abbreviations: CI, confidence interval; IPCW, inverse probability of censoring weighting; SHR, subdistribution hazard ratio.

* *P *< 0.05.

In the exploratory Fine‐Gray APOE analysis, ε4 carrier status was available for 172 of 208 participants. No statistically significant differences were observed between participants with and without APOE ε4 allele for sex, education, hypertension, DM, dyslipidemia, hearing loss, obesity, smoking, alcohol abuse, visual impairment, stroke, coronary artery disease, or atrial fibrillation. Depression was more frequent among participants without APOE genotyping than among those with available APOE data (75.76% vs 38.61%; *p* < 0.01) (Table S14). Moreover, there were no significant differences for clinical characteristics between APOE ε4 allele carriers and non‐carriers (Table S15). In the complete‐case Fine–Gray model, APOE ε4 carrier status was not significantly associated with the subdistribution hazard of dementia (SHR = 1.30; 95% CI: 0.84 to 2.01; *p* = 0.236) (Table S16). After multiple imputation, the pooled estimate remained similar in direction and magnitude (SHR = 1.35; 95% CI: 0.83 to 2.18; *p* = 0.222), with overlapping confidence intervals (Tables S17–S19). The mean imputed APOE ε4 allele carriers' prevalence among participants with missing APOE data was 44.6% across the 50 completed datasets, and the coefficient trajectories showed no marked systematic drift over the 30 interactions. These findings indicate that APOE ε4 allele missingness was associated with depression, but the APOE‐adjusted complete‐case and multiple‐imputation analyses yielded similar estimates. Further details on the imputation procedure, diagnostic assessment, and pooled analyses are provided in Appendix 2 and the Supplementary Material (Figure S4 and Tables S16–S20).

The first sensitivity analysis evaluated whether including participants with baseline‐only assessments through IPCW changed the direction or magnitude of the findings in the MCI group. The primary IPCW model yielded a lower overall annualized conversion rate than the original analysis (8.91 vs 10.72 events per 100 person‐years) while preserving the general patterns across sex and age strata. The expanded IPCW model, additionally adjusted for depression, hearing loss, and alcohol abuse, produced an overall rate of 9.91 events per 100 person‐years, with similar sex‐ and age‐related patterns but greater variability across educational categories (Table S9, Figure S5). For cumulative incidence, the primary IPCW estimates were slightly lower than the original estimates at the evaluated time points, while the expanded IPCW estimates remained generally close to those from the corresponding unweighted complete‐case analysis (Tables S10 and S11). In the Fine–Gray models, the primary IPCW analysis produced estimates similar to those of the original analysis. In contrast, the association observed for more than 8 years of education was attenuated and not reproduced in the expanded complete‐case analysis (Tables S12 and S13). Because the expanded analysis differed in both censoring‐model specification and analytic sample composition, this finding may reflect reduced precision and complete‐case selection in addition to the inclusion of depression, hearing loss, and alcohol abuse. Education‐specific estimates were therefore interpreted cautiously. Further methodological details and complete results are provided in Appendix 2.

In the recruitment‐era sensitivity analysis, participants enrolled from 2015 through 2019 had a higher subdistribution hazard of dementia conversion than those recruited up to 2014 (SHR = 1.72; 95% CI: 1.02 to 2.91; *p* = 0.043), with a stronger association among those recruited from 2020 onward (SHR = 3.13; 95% CI: 1.59 to 6.14; *p* < 0.001). Adjustment for recruitment era produced minimal changes in the estimates for age and sex. In contrast, the association for more than 8 years of education was attenuated from SHR = 2.28 (95% CI: 1.02 to 5.10; *p* = 0.044) to SHR = 1.99 (95% CI: 0.86 to 4.58; *p* = 0.106), while remaining in the same direction. Given this attenuation, the loss of statistical significance, and the variation in the proportion of highly educated participants across recruitment periods, estimates for the >8‐year education category should be interpreted cautiously. Further details are provided in Tables S21 and S22 and Appendix 2.

## 4 DISCUSSION

This study describes the methodology and key findings of the Brazilian Cog‐Aging cohort, a longitudinal study with 14 years of follow‐up, including 1270 participants accessed for eligibility. This cohort provides an opportunity to investigate dementia incidence and progression in a population characterized by low educational attainment, a high burden of cardiovascular comorbidities, and recruitment from the public healthcare system. These characteristics increase the relevance of our findings to LMICs, where sociodemographic and clinical characteristics often differ from those observed in high‐income settings. To our knowledge, this is the first prospective Brazilian cohort examining long‐term dementia outcomes in such a vulnerable population, providing critical insights into dementia risk, progression, and associated factors.

The overall annualized dementia conversion rate in MCI group was 10.72 per 100 person‐years (95% CI: 8.54 to 13.29). Overall, 40.39% of participants progressed to dementia during a mean follow‐up of 3.75 years (SD 3.28). Considering an estimated cumulative incidence of 29.85% at 3 years (95% CI: 22.74 to 36.96%), these results are similar to those reported in other LMIC studies, where MCI‐to‐dementia conversion rates ranged from 3 to 17 per 100 person‐years, corresponding to cumulative conversion rates between 6.0% and 44.8%, with a mean follow‐up of 3.7 years.^55^

Most participants in the Cog‐Aging cohort at baseline were women, consistent with demographic aging patterns observed in Brazil and worldwide, where women have a longer life expectancy and constitute a larger proportion of the oldest‐old population.^56^ Consistent with this survival advantage, dementia prevalence is generally higher among women. However, it remains unclear whether this difference is primarily attributable to longer life expectancy or to biological and pathophysiological factors that may differentially influence dementia risk and progression.^57^, ^58^ This question is particularly relevant in LMICs, where evidence remains limited.^59^ Similarly, the ELSI‐Brazil study reported a higher prevalence of dementia among women (6.8%) than among men (4.6%).^60^

In our analysis, sex‐stratified annualized incidence rates showed a higher conversion from MCI to dementia among men compared with women, corresponding to 13.79 versus 9.47 cases per 100 person‐years, respectively. To further investigate these differences while accounting for the competing risk of death, we performed sex‐stratified cumulative incidence analyses for the MCI group. Men exhibited a higher cumulative probability of dementia throughout follow‐up. At 1 year, the estimated probability of conversion was 9.99% in men and 6.79% in women; this difference increased over time, reaching 41.48% versus 24.13% at 3 years and 50.7% versus 36.51% at 5 years, respectively. Although fewer deaths occurred among men in absolute numbers, mortality was proportionally higher in this group. Because death precludes the subsequent diagnosis of dementia, competing mortality may have influenced the estimated cumulative incidence of dementia among men. However, in Fine–Gray models that include sex and adjusted for age and years of education, male sex was not significantly associated with progression to dementia in the MCI group. Limited statistical precision, particularly at later follow‐up times, should be considered when interpreting these findings.

In the MCI group, individuals aged 80 years and older had the highest annualized incidence rate of dementia, reaching 16.1 per 100 person‐years, whereas lower rates were observed in younger age groups, consistent with findings in the literature.^55^ A progressive age‐related increase in dementia risk would be expected, with older individuals showing a higher probability of conversion. However, cumulative incidence analyses revealed a different pattern, with participants aged 70 to 79 years exhibiting the lowest cumulative incidence of dementia.

Because death was treated as a competing event, the higher frequency of deaths observed in the 70‐ to 79‐year group may have reduced the cumulative incidence of dementia in that stratum, as participants who died were no longer at risk for conversion. However, when formally tested in multivariable Fine–Gray models adjusted for sex, age, and years of education, age was not significantly associated with progression to dementia in the MCI group, suggesting that the pattern observed in the crude cumulative incidence estimates was more likely attributable to differential competing mortality than to a true age effect on conversion risk.

In the CU group, age was the only variable independently associated with a higher cumulative incidence of dementia in the multivariable Fine‐Gray model. However, the small number of conversion events limited the precision of the adjusted estimates, and these findings should therefore be interpreted cautiously as exploratory and hypothesis‐generating.

We initially expected higher educational attainment to be associated with lower risk of conversion to dementia, as shown in previous studies.^54^, ^61^ However, competing mortality may partly explain the observed findings, as the association of mortality and dementia was previously demonstrated by Korhonen et al.^62^ Participants with no formal education had a higher proportion of deaths as competing events (18.5%), whereas no deaths occurred among those with more than 8 years of education. Consequently, a greater proportion of participants with no formal education died before they could develop dementia, potentially reducing the observed CIFs in this group. In contrast, the absence of competing deaths among participants with more than 8 years of education allowed more individuals to remain under observation and at risk for dementia conversion throughout follow‐up, which may have contributed to the higher cumulative incidence observed in this subgroup. This association remained statistically significant in the multivariable Fine–Gray model after adjustment for age and sex. Nevertheless, the finding should be interpreted with caution given the small size of the subgroup with more than 8 years of education, the wide confidence intervals, and the borderline statistical significance of the estimate. Therefore, this result should be considered exploratory and hypothesis‐generating rather than confirmatory.

To contextualize the recruitment‐era effects observed in our analyses, it is important to note that these eras reflect updates in clinical and diagnostic frameworks aligned with evolving research criteria, while maintaining the core DAT criteria throughout the study. Participants recruited more recently differed from those enrolled earlier, particularly in educational attainment, and they may also have differed in referral pathways, clinical presentation, and severity of cognitive impairment at assessment. The higher conversion rate among participants recruited from 2020 onward may partly reflect the COVID‐19 pandemic.

The stability of the age and sex estimates after adjusting for recruitment era suggests that these factors were unaffected by temporal changes in assessment procedures. In contrast, the attenuation of the association between higher education (>8 years) and dementia conversion highlights significant temporal heterogeneity within the cohort. Educational attainment among older adults in Brazil has increased across successive generations,^63^ and the increase in educational attainment of enrolled participants in recent years may reflect this broader demographic shift. Consequently, the initially observed association with higher education may partly reflect these compositional differences. Participants referred to the cohort during more recent phases likely differ from earlier enrollees not only in educational background but also in healthcare access, referral pathways, clinical presentation, and the stage of cognitive impairment at the time of specialist evaluation.

Furthermore, the particularly high SHR observed among participants recruited from 2020 onward may strongly reflect the impacts of the COVID‐19 pandemic. Social isolation, disruptions to routine healthcare, and delayed access to cognitive assessments likely hindered the early recognition of progressive cognitive decline by both families and professionals. As a result, some participants may have been referred to at a more advanced stage of MCI, plausibly contributing to the higher subsequent rate of dementia conversion observed in this period. Concurrently, the incorporation of the IQCODE instrument into the protocol may have improved the clinical characterization of cognitive and functional impairment in this most recent era,^64^ as well as the availability of fluid biomarkers.

Because the recruitment era serves as a composite marker, the current analysis cannot isolate the individual contributions of changing educational profiles, referral patterns, pandemic‐related delays, or evolving diagnostic frameworks. Therefore, the recruitment‐era association should not be interpreted causally. Differences in cohort composition and updates in clinical and diagnostic frameworks may have influenced the observed conversion patterns and must be carefully considered when interpreting the cohort outcomes.

Longitudinal research presents inherent challenges, particularly in LMICs. First, selection bias may be present, as participants were recruited from a tertiary geriatric referral center, which may limit the generalizability of the findings. Second, attrition and missing data may have influenced longitudinal estimates. Although participant retention was maintained over the 14‐year follow‐up period, losses were proportionally higher in the CU group, and the reasons for discontinuation were frequently unavailable. Consequently, some uncertainty remains regarding the estimation of dementia conversion and mortality rates. To mitigate these limitations, cumulative incidence functions and Fine‐Gray competing‐risk models were used to account for the competing risk of death, providing more appropriate estimates of dementia incidence than conventional survival methods that treat death as non‐informative censoring. In addition, sensitivity analyses using IPCW yielded results that were broadly consistent with the primary analyses, although residual bias due to unmeasured determinants of attrition cannot be excluded. This framework allowed for a more appropriate handling of death and reduced potential bias compared with traditional survival approaches.^65^

Attrition was likely non‐random. Retained and lost participants differed at baseline in depression, alcohol abuse, and hearing loss, which are factors that may plausibly reduce engagement with longitudinal assessments through reduced motivation, unstable routines, and communication barriers, respectively. IPCW analysis suggested these differences did not materially affect the main estimates. However, IPCW can only account for measured predictors of censoring; it cannot correct for unmeasured barriers such as changes in contact information, pandemic‐related restrictions, or transportation challenges. Residual attrition bias therefore cannot be excluded, and conversion rates should be interpreted with caution.

Even though we aimed to mitigate protocol evolution by restricting our analyses to clinical instruments that remained consistent over time, we acknowledge the potential residual effects of broader paradigm shifts. Throughout the 14‐year follow‐up, the diagnostic landscape inevitably evolved, highlighted by the transition toward biological criteria for DAT and rapid advancements in biomarker platforms. While these developments represent crucial progress in clinical diagnosis and care, they may also introduce unmeasured variance into long‐term cohort data. Therefore, some degree of residual measurement variability cannot be completely ruled out. Future analyses must carefully account for these methodological transitions when interpreting longitudinal cognitive and biological trajectories.

The Cog‐Aging study has continuously integrated validated methodologies and instruments, reflecting its commitment to evidence‐based research and the incorporation of contemporary concepts, technologies, and communication strategies (Figure S6). A major strength of this cohort is its long‐term, translational design, combining repeated and comprehensive clinical and cognitive assessment with biomarker characterization, including APOE genotyping, fluid biomarkers, and neuroimaging, available for subsets of participants. Evaluations were conducted within a multidisciplinary framework, and syndromic diagnoses were established through consensus meetings integrating based on clinical, cognitive, and functional data, with neuroimaging, fluid biomarkers, and APOE genotype subsequently integrated for etiological classification, as detailed in the “Methods” section. Standardized protocols and continuous training of researchers further enhanced data consistency and reliability.

In conclusion, we presented the structure and objectives of the Cog‐Aging study. This longitudinal cohort study has been adding valuable scientific contributions about CU, MCI, and DAT of Brazilian older adults,^17^, ^27^, ^66^, ^67^, ^68^, ^69^, ^70^ as well as fostering the training of new health professionals. The resulting biobank of clinical, cognitive, genetic, and biomarker data positions Cog‐Aging as a valuable resource for future translational research on dementia in an underrepresented, admixed Latin American population.

## AUTHOR CONTRIBUTIONS

**Gabriela Tomé Oliveira Engelmann**: Conceptualization, data curation, formal analysis, investigation, writing – original draft. **Joice Coutinho de Alvarenga**: Investigation, methodology, formal analysis, writing – original draft. **Giovanna Correia Pereira Moro**: Investigation, methodology, writing – original draft. **Ivonne Carolina Bolaños Burgos**: Investigation, methodology. **Mariana Figueiredo Miranda**: Investigation, methodology. **Millena Figueiredo Miranda**: Investigation, methodology. **Aline Siqueira‐Souza**: Investigation, methodology. **Marcelle Ferreira Saldanha**: Investigation, methodology. **Erika Oliveira Hansen**: Investigation, methodology. **Natalia Silva Dias**: Investigation, methodology. **Fabiana Carla Matos da Cunha Cintra**: Investigation, methodology. **Bruna Fulgêncio Dias**: Investigation, methodology. **Anna Luiza da Silva Souza**: Investigation, methodology. **Andréa Teixeira Carvalho**: Investigation, methodology. **Pamela V. Martino‐Adami**: Methodology, data analysis. **Marco Túlio Gualberto Cintra**: Investigation, methodology. **Ludimila Labanca**: Investigation, methodology. **Igor Nunes Lanna**: Investigation. **Rafaela Teixeira de Ávila**: Investigation, supervision, methodology. **Jonas Jardim de Paula**: Investigation, supervision, methodology. **Luciana Costa‐Silva**: Investigation. **Daniela Valadão**: Investigation, methodology. **Débora Miranda**: Supervision, resources, founding acquisition. **Marco Aurélio Romano‐Silva**: Supervision, resources, founding acquisition. **Luiz Armando Cunha de Marco**: Supervision, resources, founding acquisition. **Alfredo Ramírez**: Supervision, resources, founding acquisition, data analysis. **Bernardo de Mattos Viana**: Conceptualization, methodology, data curation, supervision, validation, visualization, writing – original draft. **Maria Aparecida Camargos Bicalho**: Project administration, conceptualization, methodology, supervision, validation, visualization, writing – original draft, resources and funding grants.

## FUNDING INFORMATION

This work was supported by grants from the following Brazilian funding agencies: Conselho Nacional de Desenvolvimento Científico e Tecnológico (CNPq) (grant numbers 47208/2013‐3, 436735/2018‐0, 309953/2018‐9, 315133/2021‐0, 406016/2025‐9) and through the National Institute of Science and Technology in Responsible Neurotechnology [INCT NeuroTec‐R] (grant no. 406935/2022‐0); Coordenação de Aperfeiçoamento de Pessoal de Nível Superior (CAPES) (grant number 88887.569376/2020‐00) and the Fundação de Amparo à Pesquisa do Estado de Minas Gerais (FAPEMIG) (grant number APQ‐02662‐14, APQ‐02465‐23, APQ‐04706‐10).

## CONFLICT OF INTEREST STATEMENT

The authors declare no conflicts of interest. Author disclosures are available in the Supporting Information.

## CONSENT STATEMENT

All participants provided a signed informed consent to join the cohort study.

## VERIFICATION

This work was approved by all authors. The manuscript was not previously published, is not under simultaneous consideration by any other journal, and will not be published elsewhere in the same form, in English or in any other language. Additionally, no ghostwriters were involved.

## Supporting information



Supporting Information

Supporting Information

Supporting Information

Supporting Information

Supporting Information
