# Supplementary material for: The Cog‐Aging: a 15‐year cohort study of cognition in Brazilian older adults
Source: Alzheimers Dement. 2026 Sep 7;22(9):e71780. doi: 10.1002/alz.71780 (PMC13550517; doi:10.1002/alz.71780)
Supplement: Supplementary file 2 [file ALZ-22-e71780-s003.docx]

**Appendix 2: Detailed Statistics**

**Supplementary Statistical Analysis**

To comprehensively evaluate the risk of conversion to dementia in this longitudinal cohort, we adopted a stepwise analytical strategy to ensure comparability with previous studies, account for competing risks, and reduce potential bias related to sample size and model overfitting.

First, incidence rates of conversion to dementia were calculated using a person-years approach. Annualized conversion rates per 100 person-years, along with 95% confidence intervals based on the Poisson distribution, were estimated. This approach allows comparison with other epidemiological and clinical cohorts.

Second, cumulative incidence functions (CIF) were estimated to evaluate the risk of conversion to dementia over time while accounting for death as a competing event. Traditional survival methods that censor competing events may overestimate dementia risk by failing to account for the fact that individuals who die are no longer at risk. Therefore, competing risk analyses were used to estimate the cumulative probability of dementia.

Third, both cumulative incidence and annualized person-year conversion rate analyses were stratified by sex, age, and educational attainment within the mild cognitive impairment (MCI) group. These complementary approaches were used to explore potential heterogeneity in dementia risk across sociodemographic subgroups while maintaining consistency between absolute risk (CIF) and incidence density measures. Stratified analyses were restricted to the MCI group because the number of dementia conversions in the cognitively unimpaired (CU) group was low.

Fourth, univariable Fine–Gray competing risk regression models were conducted to examine the association between baseline sociodemographic and clinical characteristics and conversion to dementia. The covariates were evaluated individually, and subdistribution hazard ratios were estimated to directly assess their impact on cumulative dementia incidence while retaining individuals who experienced the competing event in the risk set. This step allowed the systematic identification of relevant variables while ensuring alignment with the time-to-event structure of the data and the competing risk framework adopted in the primary analyses.

Fifth, multivariable Fine–Gray models were constructed adjusting for age, sex, and education. The relatively limited number of events, particularly in the CU group, raised concerns regarding model stability and the risk of overfitting if multiple covariates were included. Therefore, a parsimonious adjustment strategy was adopted, focusing on core sociodemographic factors known to influence dementia risk. This approach is consistent with current recommendations for competing risk regression in cohorts with a modest number of outcome events and supports the statistical adequacy and interpretability of the results. An exploratory model including sex, age, education, and APOE ε4 carriers was performed, given the well-established importance of APOE as a risk factor for Alzheimer’s dementia.

Finally, given the sample size and number of events, additional diagnostic analyses were conducted to assess the adequacy and robustness of the multivariable Fine–Gray models. These included evaluation of model assumptions, assessment of potential time-varying effects, and influence diagnostics to identify observations with disproportionate impact on model estimates.

### **Sensitivity Analysis for Early Attrition Using Inverse Probability of Censoring Weighting**

A sensitivity analysis was performed to evaluate the potential impact of early attrition on the estimates of progression to dementia. In the primary competing-risk analyses, participants who had only a baseline assessment and did not contribute longitudinal follow-up information were excluded from the analysis. We performed an inverse probability of censoring weighting (IPCW) analysis to assess whether exclusion of these participants could have introduced meaningful attrition bias.

Participants with early attrition were defined as those who had only a baseline assessment and no subsequent longitudinal follow-up visit. For the primary analysis, these participants were excluded from the dataset. For the IPCW sensitivity analysis, they were reincorporated into the dataset and treated as censored observations at baseline, while dementia conversion was treated as the event of interest and death as the competing event. Participants with only a baseline assessment were evaluated as censored observations at time zero for the IPCW sensitivity analysis. To avoid computational issues associated with zero follow-up time in time-to-event models, participants censored at baseline were assigned a minimal positive follow-up time.

The probability of remaining under follow-up was estimated using a logistic regression model. The dependent variable was early attrition, defined as having only a baseline assessment. The IPCW model included baseline age, sex, years of education, and cardiovascular comorbidity burden as predictors of follow-up retention. Cardiovascular comorbidity burden was defined as the presence of three or more cardiovascular comorbidities among hypertension, diabetes mellitus, coronary artery disease, stroke, atrial fibrillation, dyslipidemia, and obesity.

The censoring model estimated the conditional probability of early loss to follow-up given baseline covariates. Stabilized IPCW weights were then calculated as the marginal probability of remaining under follow-up divided by the predicted conditional probability of remaining under follow-up. These weights were used to reweight the analytic sample so that participants who remained under observation represented both themselves and participants with similar baseline covariate profiles who were lost after baseline.

The distribution of the stabilized IPCW weights was examined before fitting the weighted models. Weight diagnostics included the mean, median, minimum, maximum, 1st, 5th, 95th, and 99th percentiles, as well as the proportion of weights greater than 10 and greater than 20. Histograms and boxplots were also generated to visually assess the presence of influential or extreme weights. Because the estimated weights were well behaved, with means close to 1 and no extreme weights, the non-truncated stabilized IPCW weights were used in the main sensitivity analyses. Truncated weights were also generated for diagnostic purposes, but they were virtually identical to the non-truncated weights.

The distribution of stabilized IPCW weights was examined separately in the CU and MCI cohorts to evaluate weight stability and potential positivity violations. In the CU cohort, weights were centered close to 1, with a mean of 1.03 and a median of 1.04. The weights ranged from 0.70 to 1.63, with the 1st and 99th percentiles equal to 0.70 and 1.41, respectively. No weights exceeded 10 or 20. Visual inspection of the histogram and boxplot showed a narrow distribution around 1, with only one mild upper outlier and no evidence of influential extreme weights.

In the MCI cohort, IPCW weights were similarly well behaved. The mean and median weights were 1.04 and 1.11, respectively, with values ranging from 0.69 to 1.29. The 1st and 99th percentiles were 0.71 and 1.27, respectively, and no weights exceeded 10 or 20. The histogram showed a modestly bimodal distribution, suggesting some variation in the predicted probability of remaining under follow-up across covariate profiles. The boxplot showed no relevant outliers.

Overall, the IPCW diagnostics indicated stable weights in both cohorts, with no evidence of extreme weights or practical positivity violations. Therefore, the weighted sensitivity analyses were unlikely to be driven by a small number of highly influential observations. Because truncated and non-truncated weights were virtually identical, the primary IPCW analyses used the stabilized non-truncated weights.

Cumulative incidence functions (CIFs) for dementia were estimated in the presence of death as a competing event. For the primary analysis, CIFs were estimated among participants with longitudinal follow-up only. For the IPCW sensitivity analysis, participants with only baseline assessment were reincorporated as censored observations and weighted using the stabilized IPCW weights. Dementia was treated as the event of interest and death as the competing event.

For annualized conversion rates, the original analysis used only participants who contributed longitudinal follow-up time. In the IPCW sensitivity analysis, participants with only a baseline assessment were reincorporated into the analytic dataset as censored at baseline. IPCW-weighted person-years were calculated as the sum of observed follow-up time multiplied by the stabilized IPCW weight for each participant. Participants with baseline-only assessments were censored at baseline and assigned a minimal positive follow-up time to permit computational implementation; no additional follow-up time was imputed for these participants. Consequently, their direct contribution to the weighted person-time denominator was minimal. The increase in weighted person-years resulted from applying the IPCW weights to the observed follow-up time of participants who remained in the study. Weighted person-years should therefore be interpreted as person-time in the IPCW pseudo-population, rather than as additional observed follow-up time.

For cumulative incidence analyses, the original CIF was estimated among participants with longitudinal follow-up, treating death as a competing event. The IPCW-weighted CIF was then estimated after reincorporating baseline-only participants as censored observations and applying stabilized IPCW weights. The original and weighted annualized conversion rates and CIF estimates were compared to evaluate whether attrition correction meaningfully changed the estimated incidence of dementia progression.

Associations between baseline covariates and progression to dementia were evaluated using Fine–Gray subdistribution hazard models. The original univariable models were fitted in the population restricted to participants with longitudinal follow-up. To assess whether early attrition influenced these associations, the same univariable models were repeated in the IPCW-weighted analytic population, in which participants with only baseline assessments were reincorporated as censored observations at baseline and weighted using stabilized inverse probability of censoring weights. Original and IPCW-weighted univariable estimates were compared according to the direction of association, magnitude of the subdistribution hazard ratios, and 95% confidence intervals.

The multivariable model included age, sex and years of education. The primary model was fitted after excluding participants with only a baseline assessment. The IPCW-weighted sensitivity model was fitted after reincorporating these participants as censored observations and applying the stabilized IPCW weights. For each model, subdistribution hazard ratios (SHRs), 95% confidence intervals, and p-values were estimated. Model results were summarized together with the analytic sample size, the number of dementia events, and the number of competing death events.

Both univariable and multivariable IPCW-weighted Fine–Gray models were fitted using the Cox-equivalent data augmentation approach implemented through survival::finegray(), because the standard cmprsk::crr() implementation does not directly accommodate external IPCW weights. For the univariable analyses, each predictor was entered separately into the augmented weighted Cox model. For the multivariable analyses, age, sex and years of education were included simultaneously. In all IPCW-weighted models, the Fine–Gray case weights generated during data augmentation were multiplied by the stabilized IPCW weights. Standard errors clustered at the participant level were used to account for the expansion of individual participants into multiple rows.

The IPCW sensitivity analysis was designed to address early attrition among participants who completed the baseline assessment but had no subsequent longitudinal evaluation. It was not a time-dependent IPCW analysis of censoring occurring throughout follow-up. Participants who entered longitudinal follow-up contributed observed person-time until dementia conversion, death, their last available clinical assessment, or the end of follow-up, whichever occurred first. In the cumulative incidence analyses, dementia was treated as the event of interest, death as the competing event, and loss of observation after follow-up entry as right censoring at the last documented assessment. Accordingly, no person-time was assigned beyond the last observed visit. These analyses therefore account for differences in observed follow-up duration but are based on the standard independent-censoring assumption for losses occurring after longitudinal follow-up entry.

### **Expanded IPCW sensitivity analysis**

An additional inverse probability of censoring weighting sensitivity analysis was conducted in the MCI group, including both participants with longitudinal follow-up and those with only a baseline assessment, to evaluate whether further adjustment for variables associated with longitudinal follow-up altered the study findings. Depression, hearing loss, and alcohol abuse were added to the censoring model because these characteristics differed between participants with baseline-only assessments and those who contributed longitudinal follow-up, as shown in Supplementary Table S8. Their inclusion was intended to improve adjustment for the observed attrition mechanism.

The probability of remaining under longitudinal follow-up was estimated using a multivariable logistic regression model including the covariates from the primary IPCW model—age, sex, educational category, and cardiovascular comorbidity burden—together with depression, hearing loss, and alcohol abuse. Stabilized inverse probability weights were subsequently calculated from the predicted probabilities of follow-up participation and applied to the study outcomes. The resulting weights were descriptively assessed for their distribution, range, presence of extreme values, and effective sample size to evaluate potential weight instability and practical violations of the positivity assumption.

In the complete-case implementation of the expanded censoring model, which included participants with longitudinal follow-up and those with only a baseline assessment, participants were required to have observed data for all included covariates. Consequently, the expanded model was estimated in 179 participants. Because the restriction to complete cases substantially reduced the analytic sample, the expanded model was considered a complementary sensitivity analysis rather than a replacement for the primary IPCW specification.

The expanded weights were applied to the estimation of annualized dementia conversion rates, expressed as events per 100 person-years; cumulative incidence functions for dementia, with death treated as a competing event; and univariable and multivariable Fine–Gray subdistribution hazard models. The multivariable Fine–Gray models included age, sex, and educational category. Weighted conversion events and competing deaths were reported as the sums of the corresponding IPCW contributions.

The expanded model yielded a more variable weight distribution than the primary IPCW model, consistent with the smaller complete-case sample. However, no extreme weights were observed, and the effective sample size remained close to the number of complete cases, indicating no evidence of severe weight instability or a major practical violation of positivity.

Results from the expanded analysis were compared with those from both the unweighted analysis and the primary IPCW analysis. Conversion estimates and the patterns across sex and age strata were similar. Greater variability was observed in educational categories, particularly among participants with more than eight years of education. In the Fine–Gray multivariable analysis, the association originally observed for this category was attenuated in the complete-case sample. The expanded IPCW analysis differed from the original IPCW results because the censoring model additionally included depression, hearing loss, and alcohol abuse, and because the complete-case implementation restricted the analytic sample to participants with observed data for all covariates included in this expanded model. Despite the reduced sample size resulting from the complete-case implementation, the direction and magnitude of the estimates were consistent with the original analysis. The attenuation observed among participants with more than eight years of education may reflect the lower precision expected in a smaller complete-case sample and should therefore be interpreted cautiously. The education-specific estimates should be interpreted in the context of this sensitivity method rather than as a direct replacement for the primary results. The expanded analysis yielded findings generally consistent with the main results and should be interpreted as an additional sensitivity assessment incorporating depression, hearing loss, and alcohol abuse into the censoring model.

In the complete-case sample, the expanded IPCW model (including depression, hearing loss, and alcohol abuse) produced a wider and more right-skewed weight distribution than the original IPCW model. Mean stabilized weights remained close to 1 in both specifications (1.019 for the expanded model and 1.006 for the original model), although variability was greater in the expanded model (SD 0.157 vs. 0.086). Stabilized weights ranged from 0.870 to 1.886 in the expanded model and from 0.901 to 1.435 in the original model, with corresponding 99th percentiles of 1.645 and 1.335. No weights exceeded 10 or 20 in either model. The effective sample size remained close to the observed sample size for both specifications, although it was slightly lower for the expanded model (174.9 vs. 177.7). The inclusion of depression, hearing loss, and alcohol abuse modestly increased weight dispersion but did not result in extreme weights, substantial loss of effective sample size, evidence of severe weight instability, or a major practical violation of positivity.

Visual inspection of the weight distributions showed greater dispersion and right-skewness in the expanded model than in the original model. Nevertheless, most weights remained concentrated around 1, and no extreme values were observed.

**Assessment of model adequacy and robustness for the multivariable Fine–Gray competing risks model**

Given the longitudinal design of the study, the presence of death as a competing event, and the relatively limited number of dementia conversion events in some analytical strata, we performed additional diagnostic and robustness analyses to evaluate the adequacy, stability, and interpretability of the final multivariable Fine–Gray subdistribution hazards models. These analyses were conducted separately for the cognitively unimpaired (CU) and mild cognitive impairment (MCI) cohorts.

The primary adjusted Fine–Gray models included age, sex, and education as covariates. Age was modeled as a continuous variable, and sex was included as a categorical covariate. Education was modeled categorically using the same predefined groups applied in the descriptive and stratified analyses: 0 years, 1–4 years, 5–8 years, and >8 years. This specification was chosen to ensure consistency across the descriptive, stratified, cumulative incidence, and multivariable regression analyses. It also avoided imposing a strictly linear relationship between years of education and dementia conversion, which may not adequately capture the observed pattern across educational strata. Stratified analyses suggested that the association between education and conversion was not necessarily monotonic, with the >8-year group showing the highest estimated incidence rate but also wide confidence intervals. Therefore, modeling education categorically allowed the analysis to reflect potential non-linear or non-monotonic patterns while preserving clinical interpretability in a cohort characterized by low and heterogeneous educational attainment.

We evaluated the adequacy of the number of outcome events relative to model complexity by calculating the number of dementia conversion events per model parameter. This was used as an indicator of model parsimony and potential instability due to overfitting. The number of estimated parameters was derived from the final model design matrix after coding of categorical variables. This step was particularly important for the CU cohort, in which the number of dementia conversion events was substantially smaller than in the MCI cohort.

We assessed sparse-data patterns and potential quasi-complete separation. For categorical covariates, dementia events, deaths, and censored observations were tabulated across levels of sex and education. Categories with no dementia conversion events were considered suggestive of possible complete or quasi-complete separation, whereas categories with fewer than five dementia events were flagged as sparse and interpreted cautiously. For age, which was modeled continuously, we examined the distribution of dementia events across age quartiles. This descriptive assessment was used to determine whether events were concentrated in a restricted portion of the age distribution, which could contribute to instability in the estimated age effect.

In addition, we assessed numerical stability of the final multivariable Fine–Gray model. Model estimates were inspected for extreme subdistribution hazard ratios, very wide confidence intervals, missing or infinite estimates, and model-fitting warnings. Terms showing extreme estimates or unstable confidence intervals were flagged as potentially unreliable. These diagnostics were not used to automatically exclude covariates from the final model, but rather to guide interpretation of the magnitude and precision of the estimated associations.

The proportional subdistribution hazards assumption was evaluated by testing time-varying effects. Specifically, interaction terms between each model term and log-transformed follow-up time were fitted one at a time. This approach was chosen to limit model complexity, given the sample size and number of events, while still allowing assessment of whether the association between each covariate and the subdistribution hazard of dementia appeared to vary over follow-up. Statistically significant interactions were interpreted as evidence suggestive of departure from strict proportionality, rather than as definitive proof of time-varying effects, particularly in the CU cohort where the number of events was limited.

Finally, we evaluated sensitivity to influential observations using an approximate leave-one-out approach. The final multivariable Fine–Gray model was refitted sequentially after excluding each participant, and the resulting changes in regression coefficients were summarized. For each model term, we calculated the maximum and median absolute change in the regression coefficient, as well as the maximum and median percent change relative to the full model estimate. This analysis was used to identify whether the model estimates were disproportionately influenced by individual observations. Observations identified as influential were not excluded from the primary analysis unless there was a clear data-quality reason; instead, the results were used to inform the degree of caution required when interpreting model estimates.

These diagnostic procedures were designed to complement the primary Fine–Gray analyses by evaluating model adequacy, sparse-data structure, proportionality of subdistribution hazards, numerical stability, influence of individual observations, and stability of IPCW weights. The categorical specification of education improved consistency across analyses and reduced reliance on a potentially restrictive linearity assumption.

In the CU cohort, the final complete-case dataset included 15 participants with dementia conversion events and 6 deaths as competing events, corresponding to 12.6% and 5.0% of the analytical sample, respectively. The final multivariable Fine–Gray model included five estimated parameters: age, sex, and three variables for education categories, with 0 years of education used as the reference category. The events-per-parameter ratio was 3.0, indicating limited support for stable multivariable estimation and suggesting that adjusted estimates in this cohort should be interpreted cautiously. Sparse-data diagnostics did not identify warnings across sex categories, with dementia conversion events observed in both groups. However, sparse-data warnings were observed for two education strata: the 0-year education group had 3 dementia events among 15 participants, and the >8-year group had 1 event among 38 participants. Age-quartile diagnostics also suggested sparse outcome distribution, with fewer than five dementia events in the first age quartile, corresponding to ages 60–68 years, and in the third age quartile, corresponding to ages 73–78 years. Assessment of the proportional subdistribution hazards assumption showed one statistically significant time interaction, observed for age (p = .022), suggesting possible departure from strict proportionality for this covariate; no statistically significant time-varying effects were observed for sex or education categories. The approximate leave-one-out analysis showed successful refitting, with no failed refits. Influence warnings were observed for all five model terms; however, median absolute coefficient changes were small, indicating that most individual observations had limited influence on the estimates. Larger maximum coefficient changes were observed for selected terms, particularly the >8-year education category, reflecting the sparse number of events in this stratum. The CU robustness diagnostics indicated limited stability of the adjusted Fine–Gray model because of the small number of dementia conversion events, sparse events in selected education and age strata, one numerical stability warning, one significant time-interaction test, and influence-related sensitivity. Accordingly, adjusted Fine–Gray estimates in the CU cohort should be interpreted cautiously and regarded as exploratory and hypothesis-generating.

In the MCI cohort robustness diagnostics analysis, events-per-parameter ratio was 16.6, indicating an acceptable ratio between the number of dementia conversion events and model complexity. Sparse-data diagnostics did not identify any sparse-event warnings for categorical predictors. Dementia events were observed in both sex categories, with 31 events among males and 52 events among females. Dementia events were also present across all education categories: 11 events in the 0-year group, 47 in the 1–4-year group, 12 in the 5–8-year group, and 13 in the >8-year group. Similarly, age-quartile diagnostics did not show sparse-data warnings, with dementia events distributed across all age quartiles: 21 events in the first quartile, 16 in the second quartile, 17 in the third quartile, and 29 in the fourth quartile. The final multivariable Fine–Gray model did not show numerical stability warnings for any model term. The proportional subdistribution hazards assumption was evaluated through interactions between each model term and log-transformed follow-up time. No statistically significant time-varying effects were observed for age, sex, or any education category, with all interaction p-values > .05. The approximate leave-one-out influence analysis showed successful refitting of all participants, with no failed refits. Although influence warnings were identified for all five model terms based on relative percent-change thresholds, median absolute coefficient changes were small across terms, indicating that most individual observations had limited influence on the model estimates. The largest absolute coefficient changes were observed for education categories, particularly the >8-year education category, but these changes did not indicate model-fitting failure or numerical instability. Overall, the robustness diagnostics support the adequacy and stability of the adjusted Fine–Gray model in the MCI cohort, with no evidence of sparse-data problems, numerical instability, or violation of the proportional subdistribution hazards assumption. The influence analysis suggests some sensitivity of selected coefficients to individual observations, but the overall model remained stable.

**Recruitment-era sensitivity analysis**

A recruitment-era sensitivity analysis was conducted among MCI participants with longitudinal follow-up to assess whether the estimated associations with dementia conversion were influenced by temporal heterogeneity in cohort recruitment and assessment procedures. Recruitment era was defined according to the date of the baseline assessment and categorized into three mutually exclusive periods: recruitment up to 2014, recruitment from 2015 through 2019, and recruitment from 2020 onward. The earliest period was used as the reference category.

The analysis was restricted to participants with complete information on follow-up time, competing-risk outcome, age, sex, educational category, and recruitment era. Therefore, the analytic sample could differ slightly from that of the original Fine–Gray model because complete information on recruitment era was additionally required for this sensitivity analysis. Death was treated as a competing event rather than as non-informative censoring.

The distribution of participants and outcomes was summarized across recruitment eras. For each era, we calculated the number of participants, dementia conversions, deaths, and censored observations (Supplementary table 22).

The primary recruitment-era sensitivity analysis used Fine–Gray proportional subdistribution hazards regression. Two multivariable models were fitted in the same complete-case analytic sample. The original model included age, sex, and educational category. The recruitment-era-adjusted model included the same covariates with the addition of recruitment era as a categorical variable. The reference category for recruitment era was enrollment up to 2014.

For each covariate, subdistribution hazard ratios (SHRs), 95% confidence intervals, and two-sided p values were estimated. This comparison was used to evaluate whether adjustment for recruitment era altered the magnitude or direction of the associations estimated in the original multivariable model.

In the full-follow-up analysis, the complete-case sample included 205 participants with MCI, including 83 dementia conversions and 14 competing deaths. Recruitment from 2015 through 2019 was associated with a higher subdistribution hazard of dementia conversion compared with recruitment up to 2014 (SHR = 1.72; 95% CI: 1.02–2.91; p = 0.043). Recruitment from 2020 onward was associated with a larger increase in the subdistribution hazard (SHR = 3.13; 95% CI: 1.59–6.14; p < 0.001).

Adjustment for recruitment era produced little change in the age estimate, with the SHR changing from 1.034 in the original model to 1.037 in the recruitment-era-adjusted model. The estimate for sex also remained statistically non-significant. In contrast, the estimate for more than 8 years of education was attenuated from an SHR of 2.28 (95% CI: 1.02–5.10; p = 0.044) in the original model to an SHR of 1.99 (95% CI: 0.86–4.58; p = 0.106) after adjustment for recruitment era. Although the direction of the association was maintained, the confidence interval included the null after recruitment-era adjustment.

The recruitment-era coefficients were interpreted as measures of temporal heterogeneity in the subdistribution hazard of dementia conversion, conditional on the covariates included in the model. They were not interpreted as causal effects of calendar period, because recruitment era represents a composite indicator that may capture simultaneous changes in cohort composition, referral patterns, diagnostic ascertainment, assessment procedures, documentation, and availability of complementary diagnostic information.

### **Multiple imputation of missing APOE genotype data**

Multiple imputation was performed as an exploratory sensitivity analysis to evaluate whether incomplete APOE genotype information affected the APOE-adjusted multivariable Fine–Gray regression estimates. The analysis was restricted to participants with mild cognitive impairment who had longitudinal follow-up data available.

APOE was available for 172 participants and missing in 36 participants, corresponding to 17.3% of the analytic sample. Among participants with observed APOE data, 37.8% were APOE ε4 carriers.

APOE missingness was not assumed to be completely at random. The imputation procedure therefore incorporated variables potentially associated with APOE status, the availability of genotyping, baseline clinical characteristics, cognitive performance, and dementia progression. Because this analysis was intended to assess the robustness of the APOE-adjusted findings rather than replace the primary analysis, it was treated as an exploratory sensitivity analysis.

Missing data were addressed using substantive-model-compatible fully conditional specification adapted for a Fine–Gray competing-risks model, implemented with the smcfcs.finegray() function in R. This approach was selected to preserve compatibility between the conditional imputation models and the Fine–Gray model used for the outcome analysis.

The substantive Fine–Gray model included age, sex, education, and APOE ε4 carriers status. It was coded as carrier versus non-carrier. Education was represented using the same categories applied in the primary competing-risks analysis.

Follow-up time, dementia conversion status, and competing death status were incorporated directly through the Fine–Gray-compatible imputation procedure. Dementia was specified as the event of interest, death as the competing event, and participants who experienced neither outcome were treated as censored.

The following auxiliary variables were included in the imputation system: family history of dementia, dyslipidemia, obesity, depression, hypertension, diabetes mellitus, and year of recruitment.

Depression was included because the descriptive comparison showed a higher prevalence of depression among participants without APOE genotyping. Recruitment year was included to account for temporal variation in the availability and use of APOE genotyping during the study period.

The proportions of observations with missing data were 0.96% for education, 11.1% for family history of dementia, 3.9% for dyslipidemia, 17.8% for obesity, 7.2% for depression, 1.9% for hypertension, and 2.9% for diabetes mellitus.

Among the 36 participants with missing APOE data, age, sex, and recruitment year were available for all participants. Education was available for 94.4%, depression and hypertension for 97.2%, diabetes mellitus for 94.4%, dyslipidemia for 91.7%, obesity for 77.8%, and family history of dementia for 50.0%.

The descriptive findings indicate that APOE missingness should not be regarded as completely random. Multiple imputation was therefore conducted under a missing-at-random assumption conditional on the observed variables included in the imputation model.

Convergence was assessed by examining the evolution of the substantive-model coefficients across successive iterations and by comparing their mean values during the initial and final cycles. No substantial systematic drift was observed. Standardized differences between the initial and final cycles ranged from approximately 0.033 to 0.106, indicating that the substantive-model coefficient trajectories were stable over the 30 iterations.

The plausibility of the imputed APOE distribution was assessed across all 50 completed datasets. Among participants with observed APOE data, the prevalence of APOE ε4 carriers was 37.8%. Among participants whose APOE status was originally missing, the mean imputed carrier prevalence was 44.6%.

Across the 50 imputations, the carrier prevalence among participants with missing APOE data had a standard deviation of 5.8 percentage points. The median imputed prevalence was 44.4%, with an interquartile range of 41.7%–47.2% and an overall range of 33.3%–61.1%. Thus, the imputation procedure generated meaningful between-imputation variability and did not deterministically assign all participants with missing APOE data to a single carrier category.

Observed and imputed distributions of the auxiliary variables were also examined and the imputed distributions were considered compatible with the observed data. The multiple-imputation analysis was interpreted in conjunction with the substantive-model coefficient trajectories, the plausibility of the imputed APOE distribution, and the consistency between the complete-case and multiply imputed Fine–Gray estimates.

The multivariable Fine–Gray model was fitted separately in each of the 50 completed datasets. Regression coefficients and variance estimates were subsequently combined using Rubin’s rules, incorporating both within-imputation and between-imputation variability.

For the APOE carrier coefficient, the estimated fraction of missing information was approximately 12.8%. The relative efficiency associated with the use of 50 imputations was 99.7%, indicating that the number of imputations was adequate for the estimated degree of missing information.

The complete-case analysis included 172 participants, 78 dementia events, and 14 competing deaths. APOE ε4 carriers status was not statistically significantly associated with the subdistribution hazard of dementia. The estimated subdistribution hazard ratio was 1.30 (95% confidence interval 0.84–2.01; p=0.236).

After multiple imputation, all 208 participants were included, with 84 dementia events and 14 competing deaths. The pooled subdistribution hazard ratio for APOE carrier status was 1.35 (95% confidence interval 0.83–2.18; p=0.222).

The complete-case and multiply imputed APOE estimates were similar in direction and magnitude, with substantially overlapping confidence intervals. Under the assumptions of the imputation model, accounting for incomplete APOE data did not materially alter the APOE point estimate. This agreement should not be interpreted as proof that APOE missingness introduced no bias, but it indicates that the estimated association was consistent across the two missing-data approaches.
