# Supplementary material for: The Cog‐Aging: a 15‐year cohort study of cognition in Brazilian older adults
Source: Alzheimers Dement. 2026 Sep 7;22(9):e71780. doi: 10.1002/alz.71780 (PMC13550517; doi:10.1002/alz.71780)
Supplement: Supplementary file 3 [file ALZ-22-e71780-s004.docx]

**Appendix 1:** Normative from cognitive tools.

1. ALMEIDA OP. (1998). Mini exame dos estado mental e o diagnóstico de demência no Brasil. Arquivos de Neuro-Psiquiatria, doi.org/10.1590/S0004-282X1998000400014.
2. American Psychiatric Association. The Diagnostic and Statistical Manual of Mental Disorders. 5th ed. ed: American Psychiatric Association Publishing; 2013.
3. Assis L.d.O., Paula J.J.d., Assis M.G., Moraes E.N.d., Malloy-Diniz L.F. (2014). Psychometric properties of the Brazilian version of Pfeffer's Functional Activities Questionnaire. Frontiers in Aging Neuroscience, doi: 10.3389/fnagi.2014.00255.
4. Beato R.G., Nitrini R., Formigoni A.P., Caramelli P. (2007). Brazilian version of the Frontal Assessment Battery (FAB): Preliminary data on administration to healthy elderly. Dementia & Neuropsychologia, doi.org/10.1590/S1980-57642008DN10100010.
5. Bertolucci P.H.F., Okamoto I.H., Brucki S.M.D., Siviero M.O., Toniolo Neto J., Ramos L.R. Applicability of the CERAD neuropsychological battery to Brazilian elderly. (2001). Arquivos de Neuro-Psiquiatria, doi.org/10.1590/S0004-282X2001000400009.
6. Brucki S.M.D., Malheiros S.M.F., Okamoto I.H., Bertolucci P.H.F. (1997). Dados normativos para o teste de fluência verbal categoria animais em nosso meio. Arquivos de Neuro-Psiquiatria, <http://doi.org/10.1590/S0004-282X1997000100009>.
7. Cunha Cintra F., Cintra M., Nicolato R., Bertola L., Ávila R., Malloy-Diniz L., et al. (2017). Functional decline in the elderly with MCI: Cultural adaptation of the ADCS-ADL scale. Revista da Associação Médica Brasileira, doi: 10.1590/1806-9282.63.07.590.
8. Morris J.C., Heyman A., Mohs R.C., Hughes J.P., Belle G. v., Fillenbaum G., et al. (1989). The Consortium to Establish a Registry for Alzheimer's Disease (CERAD). Part I. Clinical and neuropsychological assessment of Alzheimer's disease, doi: 10.1212/wnl.39.9.1159.
9. Nitrini R., Lefèvre H.B., Mathias S.C., Caramelli P., Carrilho P.E.M., Sauaia N., et al. (1994). Testes neuropsicológicos de aplicação simples para o diagnóstico de demência. Arquivos de Neuro-Psiquiatria, doi.org/10.1590/S1980-57642008DN10100010.
