# Supplementary material for: The Cog‐Aging: a 15‐year cohort study of cognition in Brazilian older adults
Source: Alzheimers Dement. 2026 Sep 7;22(9):e71780. doi: 10.1002/alz.71780 (PMC13550517; doi:10.1002/alz.71780)
Supplement: Supplementary file 4 [file ALZ-22-e71780-s001.zip › Sup_Table 1_jul21.docx]

**Supplementary Table 1***. Number of follow-up visits.*

| **Follow up** | **Number of Visits** |
| --- | --- |
| Baseline | 328 |
| Year 1 (±6m) | 263 |
| Year 2 (±6m) | 208 |
| Year 3 (±6m) | 134 |
| Year 4 (±6m) | 85 |
| Year 5 (±6m) | 81 |
| Year 6 (±6m) | 61 |
| Year 7 (±6m) | 56 |
| Year 8 (±6m) | 55 |
| Year 9 (±6m) | 37 |
| Year 10 (±6m) | 32 |
| Year 11 (±6m) | 25 |
| Year 12 (±6m) | 21 |
| Year 13 (±6m) | 6 |
| Year 14 (±6m) | 2 |
| Year 15 (±6m) | 1 |
