# Supplementary material for: The Cog‐Aging: a 15‐year cohort study of cognition in Brazilian older adults
Source: Alzheimers Dement. 2026 Sep 7;22(9):e71780. doi: 10.1002/alz.71780 (PMC13550517; doi:10.1002/alz.71780)
Supplement: Supplementary file 4 [file ALZ-22-e71780-s001.zip › Sup_table 2_jul21.docx]

**Supplementary Table 2**. Evolution of diagnostic frameworks and corresponding clinical variables systematically collected in the Cog-Aging cohort.

| **Mild Cognitive Impairment** | |  | | | |  | |  |
| --- | --- | --- | --- | --- | --- | --- | --- | --- |
| **Construct** | | **2011-2014** | | | | **2015-2025** | |  |
| Cognitive complaint | | Yes | | | | Yes | |  |
| Objective Cognitive testing | | Yes | | | | Yes | |  |
| Functional assessment | | Yes | | | | Yes | |  |
| Consensus diagnosis | | Yes | | | | Yes | |  |
| Neuropsychological assessment | | Yes | | | | Yes | |  |
| **Major Neurocognitive Disorder** | | |  | | | | | |
| **Diagnostic Domain** | **DSM-IV Criteria**  **(2011-2014)** | | | **DSM-5**  **(2015-2025)** | **McKhann**  **/ NIA-AA (2011)** | | **Variable systematically collected in Cog-Aging** | |
| **Objective cognitive impairment** | Required (Memory + at least 1 other domain) | | | Required (Significant decline in 1 or more domains) | Required (At least 2 domains) | | Comprehensive neuropsychological assessment | |
| **Functional independence** | Impaired (Significant interference with work/social activities) | | | Impaired (Interferes with independence in everyday activities) | Impaired (Interfere with the ability to function at work or at usual activities) | | Functional assessment | |
| **History of cognitive decline** | Required (Gradual onset and continuing cognitive decline) | | | Required (Concern of significant decline by individual, informant, or clinician) | Required (Decline from previous levels of performance) | | Structured clinical interview and informant report | |
| **Exclusion of other conditions** | Required (Not due to other CNS or systemic conditions) | | | Required (Deficits do not occur exclusively in the context of a delirium) | Required (symptoms not explained by delirium or major psychiatric disorder) | | Multidisciplinary consensus clinical evaluation | |
| **Etiological specifications** | Primarily clinical (NINCDS-ADRDA/DSM-IV) | | | - | Clinical + Biomarkers | | Consensus diagnosis + integration of biomarker data (when available) | |
| Abbreviations: ADRDA: *Alzheimer's Disease and Related Disorders Association;* CNS= Central Nervous System; NIA-AA**:** *National Institute on Aging;* NINCDS: *National Institute of Neurological and Communicative Disorders and Stroke;* DSM: *Diagnostic and Statistical Manual of Mental Disorders.* | | | | | | | | |
