# Supplementary material for: The Cog‐Aging: a 15‐year cohort study of cognition in Brazilian older adults
Source: Alzheimers Dement. 2026 Sep 7;22(9):e71780. doi: 10.1002/alz.71780 (PMC13550517; doi:10.1002/alz.71780)
Supplement: Supplementary file 4 [file ALZ-22-e71780-s001.zip › Sup_table 3_jul21.docx]

**Supplementary Table 3**. Operationalized criteria and cut-off scores for functional and cognitive assessments.

| **Domain** | **Assessment Tool/ Scale** | **Target Classification** | **Cutoff Criterion** |
| --- | --- | --- | --- |
| **Cognitive** | Brief Cognitive Screening Battery | Naming | 9/10 |
|  | Figure memory tests |  |  |
|  |  | Incidental memory | 4/5 |
|  |  |  |  |
|  |  | Immediate memory | 6/7 |
|  |  |  |  |
|  |  | Learning | 6/7 |
|  |  |  |  |
|  |  | Delay recall | 5/6 |
|  |  |  |  |
|  |  | Recognition | 7/8 |
|  |  |  |  |
|  | CERAD Word List | Sum of three attempts | 13/14 |
|  |  | Delayed Recall | 3/4 |
|  |  |  |  |
|  | DSM-5 Criteria | Mild Neurocognitive Disorder (MCI) | -1.5 to -2.0 SD |
|  |  |  |  |
|  |  | Major Neurocognitive Disorder (Dementia) | <-2.0 SD |
|  |  |  |  |
|  | Fluency Animals/ Fruits | Low education | 8/9 |
|  |  |  |  |
|  |  | High education | 12/13 |
|  |  |  |  |
|  | Frontal Assessment Battery |  | 11/12 |
|  |  |  |  |
|  | Mini-Mental State Examination | Illiterate | 19/20 |
|  |  |  |  |
|  |  | Literate | 23/24 |
|  |  |  |  |
| **Functional** | Pfeffer Functional Activities Questionnaire | Functional Impairment | 4/5 points |
|  |  |  |  |
|  | Alzheimer’s Disease Cooperative Study (ADCS) | Functional Decline (MCI) | 71/72 points |
|  |  |  |  |
|  |  | Functional Decline (Dementia) | 64/65 points |
|  |  |  |  |
|  |  | Advanced Activities of Daily Living – Functional Impairment (MCI) | 17/18 points |
|  |  |  |  |
| Abbreviations: Consortium to Establish a Registry for Alzheimer’s Disease (CERAD); Diagnostic and Statistical Manual of Mental Disorders (DSM-5); Mild Cognitive Impairment (MCI); Standard Deviation (SD). | | | |
