# Supplementary material for: The Cog‐Aging: a 15‐year cohort study of cognition in Brazilian older adults
Source: Alzheimers Dement. 2026 Sep 7;22(9):e71780. doi: 10.1002/alz.71780 (PMC13550517; doi:10.1002/alz.71780)
Supplement: Supplementary file 4 [file ALZ-22-e71780-s001.zip › Sup_table 4_jul21.docx]

|  | **Supplementary Table 4.** Geriatric Cognitive Assessment and Neuropsychological Evaluation Protocol of Cog-Aging. | |  |  |  |
| --- | --- | --- | --- | --- | --- |
|  | **Brief cognitive assessment** | **Neuropsychological assessment** |  |  |  |
|  | Mini Mental State Examination (MMSE);  Word List of the Consortium to Establish a Registry for Alzheimer’s Disease (CERAD);  Frontal Assessment Battery (FAB);  Semantic Verbal Fluency (fruits and animals);  Alternating Verbal Fluency (animals X fruits);  Clock Drawing Test (Shulman);  Pfeffer Functional Activities Questionnaire (FAQ);  Informant Questionnaire on Cognitive Decline in the Elderly (IQCODE);  Clinical Dementia Scale (CDR) - Sum of Boxes (CDR-SOB) | **Intellectual performance:**  Wechsler Abbreviated Scale of Intelligence (WASI) - Vocabulary + Matrix Reasoning |  |  |  |
|  |  | **Global Cognition:** Mini-Mental State Examination (MMSE); Mattis Dementia Rating Scale (MDRS); |  |  |  |
|  |  | **Language:** Naming Test from the Laboratory of Neuropsychological Investigations (TN-LIN); Phonological Verbal Fluency (FAS); Vocabulary (WASI); |  |  |  |
|  |  | **Memory:** Memory (DRS); Rey Verbal Auditory Learning Test (RAVLT); Simplified Taylor Complex Figure Test (sTCFT) - immediate and delayed recall; |  |  |  |
|  |  | **Executive Functions:** Initiation/Perseveration (MDRS); Conceptualization (MDRS); Frontal Assessment Battery (FAB); Five Digits Test; Tower of London Test; Digit Span; |  |  |  |
|  |  | **Perceptomotor:** Construction (MDRS); sTCFT (copy) The Stick Design test; |  |  |  |
|  |  | **Attention/ Processing Speed:** Attention (MDRS); Five Digit Test; Digit Span; |  |  |  |
|  |  | **Neuropsychiatric symptoms:** Neuropsychiatric Inventory (NPI) |  |  |  |
|  |  | **Functional abilities in daily living:** Pfeffer Functional Activities Questionnaire (FAQ); Activities of Daily Living Questionnaire for Elderly with Mild Cognitive Impairment (ADCS-ADL) |  |  |  |
|  |  |  |  |  |  |
|  | Abbreviations: Activities of Daily Living Questionnaire for Elderly with Mild Cognitive Impairment (ADCS-ADL); Consortium to Establish a Registry for Alzheimer’s Disease (CERAD); Clinical Dementia Scale (CDR) - Sum of Boxes (CDR-SOB); Frontal Assessment Battery (FAB); Informant Questionnaire on Cognitive Decline in the Elderly (IQCODE); Mattis Dementia Rating Scale (MDRS); Mini-Mental State Examination (MMSE); Naming Test from the Laboratory of Neuropsychological Investigations (TN-LIN); Neuropsychiatric Inventory (NPI); Pfeffer Functional Activities Questionnaire (FAQ); Phonological Verbal Fluency (FAS); Rey Verbal Auditory Learning Test (RAVLT); Simplified Taylor Complex Figure Test (sTCFT); Wechsler Abbreviated Scale of Intelligence (WASI). | |  |  |  |
