# Supplementary material for: The Cog‐Aging: a 15‐year cohort study of cognition in Brazilian older adults
Source: Alzheimers Dement. 2026 Sep 7;22(9):e71780. doi: 10.1002/alz.71780 (PMC13550517; doi:10.1002/alz.71780)
Supplement: Supplementary file 4 [file ALZ-22-e71780-s001.zip › Sup_table 5_jul21.docx]

**Supplementary Table 5.** Strategies for Retention and Maintenance of participants in Cog-Aging Study.

| **Main Strategy Domain** | **Activities Employed** |
| --- | --- |
| **Community building strategies** | Logo creation with color schemes |
| **Reminder Strategies** | - Telephone and instant messaging (WhatsApp);  - Messaging profile displayed the study logo;  - Researchers identified themselves by name and Affiliation in all communications;  - Messages included the facility address and local landmarks to assist with navigation;  - Participants were explicitly reminded to bring essential items for testing, including corrective lenses and hearing aids; current medications; previous clinical examination results. |
| **Tracking Strategies** | Contact information (address, phone number and email addresses) was also recorded for a designated informant or collateral source for each participant to facilitate scheduling. |
| **Maintain continuity of interviewer-participant relationship** | -To maintain continuity and rapport, follow-up assessments were conducted by the same researcher whenever possible, unless a change was requested by the participants.  - The principal investigators (PIs) met personally with participants to introduce themselves and the project, and to explain the scientific relevance of the study and the importance of their participation. |
| **Minimize Participant Burden** | -Refreshments were provided for both participants and informants, during the comprehensive health assessment.  -Transportation support (bus tickets) was provided to informants upon request. Participants did not require this assistance, as they are legally entitled to free public transportation; |
