# Supplementary material for: The Cog‐Aging: a 15‐year cohort study of cognition in Brazilian older adults
Source: Alzheimers Dement. 2026 Sep 7;22(9):e71780. doi: 10.1002/alz.71780 (PMC13550517; doi:10.1002/alz.71780)
Supplement: Supplementary file 4 [file ALZ-22-e71780-s001.zip › Sup_Table 6_jul21.docx]

**Supplementary Table 6.** Biomarkers distributions among participants at baseline.

| **Biomarker** | **CSF Aβ+** | **Plasma pTau217+** | **APOE ε4 carriers** |
| --- | --- | --- | --- |
| **Total cohort** |  |  |  |
| Total n available | 97 | 299 | 502 |
| % of positive | 53.61% | 34.78% | 37.1% |
| **Cognitive Unimpaired** |  |  |  |
| Total n available | 19 | 92 | 157 |
| % of positive | 15.79% | 11.96% | 20.4% |
| **Mild Cognitive Impairment** |  |  |  |
| Total n available | 31 | 134 | 217 |
| % of positive | 38.71% | 26.86% | 41.0% |
| **Dementia** |  |  |  |
| Total n available | 46 | 73 | 128 |
| % of positive | 86.96% | 78.1% | 50.8% |

Abbreviations: Amyloid Beta (Aβ); APOE ε4 carriers = ε4 allele of the apolipoprotein 4 gene; Cerebrospinal Fluid (CSF); Phosphorylated tau (p-Tau).
