# Supplementary material for: The Cog‐Aging: a 15‐year cohort study of cognition in Brazilian older adults
Source: Alzheimers Dement. 2026 Sep 7;22(9):e71780. doi: 10.1002/alz.71780 (PMC13550517; doi:10.1002/alz.71780)
Supplement: Supplementary file 4 [file ALZ-22-e71780-s001.zip › Sup_Table 7_jul21.docx]

**Supplementary Table 7.** Follow-up sociodemographic and clinical characteristics.

| **Characteristic** | **CU** **N = 120** | **Missing CU data** | **MCI** **N = 208** | **Missing MCI data** |
| --- | --- | --- | --- | --- |
| **Education** | 6.7 (5.3) | 1 (0.8%) | 4.7 (4.0) | 2 (1.0%) |
| **Age** | 73.2 (6.9) | 1 (0.8%) | 75.0 (6.8) | 0 (0.0%) |
| **Sex** |  |  |  |  |
| Female | 88 (73.3%) | 0 (0.0%) | 144 (69.2%) | 0 (0.0%) |
| **Follow-up time (years)** | 5.0 (3.9) | 0 (0.0%) | 3.7 (3.3) | 0 (0.0%) |
| **Hypertension** | 86 (71.7%) | 0 (0.0%) | 151 (74.0%) | 4 (1.9%) |
| **Diabetes Mellitus** | 28 (23.5%) | 1 (0.8%) | 63 (31.2%) | 6 (2.9%) |
| **Dyslipidemia** | 55 (46.2%) | 1 (0.8%) | 99 (49.5%) | 8 (3.8%) |
| **Depression** | 52 (46.8%) | 9 (7.5%) | 87 (45.1%) | 15 (7.2%) |
| **Hearing Loss** | 39 (38.2%) | 18 (15.0%) | 57 (34.1%) | 41 (19.7%) |
| **Obesity** | 29 (26.6%) | 11 (9.2%) | 40 (23.3%) | 36 (17.3%) |
| **Smoking** | 34 (31.5%) | 12 (10.0%) | 55 (30.6%) | 28 (13.5%) |
| **Alcohol Abuse** | 48 (45.3%) | 14 (11.7%) | 75 (41.7%) | 28 (13.5%) |
| **Visual Impairment** | 45 (45.0%) | 20 (16.7%) | 62 (37.1%) | 41 (19.7%) |
| **Stroke** | 7 (6.1%) | 5 (4.2%) | 14 (6.9%) | 4 (1.9%) |
| **Coronary artery disease** | 10 (9.3%) | 13 (10.8%) | 17 (9.1%) | 21 (10.1%) |
| **Atrial fibrillation** | 6 (5.9%) | 19 (15.8%) | 9 (5.5%) | 43 (20.7%) |
| **APOE ε4 carriers** | 19 (18.6%) | 18 (15.0%) | 65 (37.8%) | 36 (17.3%) |

Abbreviations: APOE ε4 carriers = ε4 allele of the apolipoprotein 4 gene; CU: Cognitively Unimpaired; MCI: Mild Cognitive Impairment; Mean (Standard Deviation).
