# Supplementary material for: The Cog‐Aging: a 15‐year cohort study of cognition in Brazilian older adults
Source: Alzheimers Dement. 2026 Sep 7;22(9):e71780. doi: 10.1002/alz.71780 (PMC13550517; doi:10.1002/alz.71780)
Supplement: Supplementary file 4 [file ALZ-22-e71780-s001.zip › Sup_table 8_jul21.docx]

| **Table S8.** Baseline Sociodemographic and clinical Characteristics comparisons between retained and lost participants | | | |
| --- | --- | --- | --- |
| **Variable** | **Not-Followed** | **Followed** | **P-value**^*^ |
| ***OVERALL*** |  |  |  |
| **Education** | 6.06 (5) | 5.45 (4.64) | 0.155 |
| **Age** | 74.22 (7.72) | 74.37 (6.88) | 0.676 |
| **Sex** |  |  |  |
| Female | 230 (71%) | 232 (70.7%) | 1.000 |
| Male | 94 (29%) | 96 (29.3%) | 1.000 |
| **Hypertension** | 173 (68.1%) | 237 (73.1%) | 0.218 |
| **Diabetes Mellitus** | 89 (35.2%) | 91 (28.3%) | 0.097 |
| **Dyslipidemia** | 70 (46.1%) | 154 (48.3%) | 0.724 |
| **Depression** | 99 (42.9%) | 139 (45.7%) | 0.567 |
| **Hearing Loss** | 29 (18.8%) | 96 (35.8%) | <0.001^†^ |
| **Smoking** | 29 (35.8%) | 89 (30.9%) | 0.484 |
| **Alcohol Abuse** | 23 (28%) | 123 (43%) | 0.021^†^ |
| **Visual Impairment** | 50 (32.5%) | 107 (40.1%) | 0.147 |
| **Stroke** | 15 (6.7%) | 21 (6.6%) | 0.999 |
| **Coronary artery disease** | 11 (6.4%) | 27 (9.2%) | 0.366 |
| **Atrial fibrillation** | 1 (1.9%) | 15 (5.6%) | 0.485 |
| **Obesity** | 35 (27.3%) | 69 (24.6%) | 0.917 |
| **APOE ε4 carriers** | 33 (34%) | 84 (30.7%) | 0.627 |
| ***CU*** |  |  |  |
| **Education** | 7.63 (5.12) | 6.69 (5.3) | 0.129 |
| **Age** | 72.18 (7.4) | 73.25 (6.84) | 0.135 |
| **Sex** |  |  |  |
| Female | 119 (77.3%) | 90 (73.8%) | 0.594 |
| Male | 35 (22.7%) | 32 (26.2%) | 0.594 |
| **Hypertension** | 77 (68.8%) | 86 (70.5%) | 0.883 |
| **Diabetes Mellitus** | 37 (33%) | 28 (23.1%) | 0.124 |
| **Dyslipidemia** | 42 (47.7%) | 56 (46.3%) | 0.947 |
| **Depression** | 27 (28.4%) | 53 (46.9%) | 0.010^†^ |
| **Hearing Loss** | 8 (17%) | 40 (38.8%) | 0.014^†^ |
| **Smoking** | 18 (42.9%) | 35 (31.8%) | 0.277 |
| **Alcohol Abuse** | 15 (34.9%) | 49 (45.4%) | 0.320 |
| **Visual Impairment** | 14 (31.1%) | 46 (45.1%) | 0.159 |
| **Stroke** | 5 (5.8%) | 7 (6%) | 0.999 |
| **Coronary artery disease** | 3 (5.1%) | 10 (9.2%) | 0.546 |
| **Atrial fibrillation** | 1 (4.2%) | 6 (5.9%) | 0.999 |
| **Obesity** | 24 (31.6%) | 30 (27%) | 0.812 |
| **APOE ε4 carriers** | 12 (21.8%) | 19 (18.3%) | 0.744 |
| ***MCI*** |  |  |  |
| **Education** | 4.62 (4.43) | 4.72 (4.03) | 0.680 |
| **Age** | 76.05 (7.57) | 75.02 (6.84) | 0.175 |
| **Sex** |  |  |  |
| Female | 111 (65.3%) | 142 (68.9%) | 0.524 |
| Male | 59 (34.7%) | 64 (31.1%) | 0.524 |
| **Hypertension** | 96 (67.6%) | 151 (74.8%) | 0.184 |
| **Diabetes Mellitus** | 52 (36.9%) | 63 (31.5%) | 0.358 |
| **Dyslipidemia** | 28 (43.8%) | 98 (49.5%) | 0.512 |
| **Depression** | 72 (52.9%) | 86 (45%) | 0.194 |
| **Hearing Loss** | 21 (19.6%) | 56 (33.9%) | 0.015^†^ |
| **Smoking** | 11 (28.2%) | 54 (30.3%) | 0.944 |
| **Alcohol Abuse** | 8 (20.5%) | 74 (41.6%) | 0.023^†^ |
| **Visual Impairment** | 36 (33%) | 61 (37%) | 0.590 |
| **Stroke** | 10 (7.2%) | 14 (6.9%) | 1.000 |
| **Coronary artery disease** | 8 (7%) | 17 (9.2%) | 0.657 |
| **Atrial fibrillation** | 0 (0%) | 9 (5.5%) | 0.362 |
| **Obesity** | 11 (21.2%) | 39 (22.8%) | 0.961 |
| **APOE ε4 carriers** | 21 (50%) | 65 (38.2%) | 0.224 |

Abbreviations: APOE ε4 carriers = ε4 allele of the apolipoprotein 4 gene. Footnotes:^*^p-value considered for Chi-square, Fisher Exact, Mann-Whitney, Student-t test as appropriate for each variable; ^†^p<0.05.
