# Supplementary material for: The Cog‐Aging: a 15‐year cohort study of cognition in Brazilian older adults
Source: Alzheimers Dement. 2026 Sep 7;22(9):e71780. doi: 10.1002/alz.71780 (PMC13550517; doi:10.1002/alz.71780)
Supplement: Supplementary file 4 [file ALZ-22-e71780-s001.zip › Sup_table 9_jul21.docx]

**Supplementary Table 9.** MCI group overall and Stratified Annualized Dementia Conversion Rates by sex, age and education

| **Characteristic** | **N** | **Dementia events** | **Person-years** | **Rate per 100 person-years**  **(95% CI)** |
| --- | --- | --- | --- | --- |
| *Original analysis (excluding baseline-only participants)* | | | | |
| Overall | 206 | 83.0 | 774.1 | 10.72 (8.54–13.29) |
| **Sex** |  |  |  |  |
| Male | 63 | 31.0 | 224.8 | 13.79 (9.37–19.57) |
| Female | 143 | 52.0 | 549.3 | 9.47 (7.07–12.41) |
| **Age group** |  |  |  |  |
| 60–69 years | 47 | 21.0 | 199.8 | 10.51 (6.51–16.07) |
| 70–79 years | 105 | 33.0 | 394.3 | 8.37 (5.76–11.75) |
| ≥80 years | 54 | 29.0 | 180.0 | 16.11 (10.79–23.14) |
| **Education** |  |  |  |  |
| 0 years | 27 | 11.0 | 108.4 | 10.15 (5.06–18.15) |
| 1–4 years | 116 | 47.0 | 458.4 | 10.25 (7.53–13.63) |
| 5–8 years | 30 | 12.0 | 124.8 | 9.62 (4.97–16.80) |
| >8 years | 33 | 13.0 | 82.5 | 15.76 (8.39–26.95) |
| *Sensitivity analysis (baseline-only included, IPCW-weighted)* | | | | |
| Overall | 370 | 84.0 | 942.9 | 8.91 (7.11–11.03) |
| **Sex** |  |  |  |  |
| Male | 120 | 32.3 | 296.4 | 10.88 (7.46–15.34) |
| Female | 250 | 51.8 | 646.6 | 8.01 (5.98–10.51) |
| **Age group** |  |  |  |  |
| 60–69 years | 84 | 20.4 | 227.9 | 8.95 (5.50–13.77) |
| 70–79 years | 169 | 32.4 | 452.2 | 7.17 (4.92–10.11) |
| ≥80 years | 117 | 31.2 | 262.9 | 11.87 (8.07–16.83) |
| **Education** |  |  |  |  |
| 0 years | 53 | 10.8 | 139.6 | 7.74 (3.84–13.93) |
| 1–4 years | 210 | 47.7 | 553.1 | 8.63 (6.36–11.45) |
| 5–8 years | 51 | 12.3 | 146.3 | 8.43 (4.40–14.62) |
| >8 years | 56 | 13.2 | 104.0 | 12.65 (6.77–21.57) |
| *Sensitivity analysis (baseline-only included, IPCW-weighted; expanded model adjusted for age, sex, education, ≥3 cardiovascular comorbidities, depression, hearing loss, and alcoholism)* | | | | |
| Overall | 179 | 63.2 | 637.4 | 9.91 (7.62–12.68) |
| **Sex** |  |  |  |  |
| Male | 58 | 23.6 | 180.4 | 13.08 (8.35–19.53) |
| Female | 121 | 39.6 | 457.0 | 8.66 (6.17–11.81) |
| **Age group** |  |  |  |  |
| 60–69 years | 41 | 17.1 | 172.0 | 9.96 (5.82–15.92) |
| 70–79 years | 87 | 22.5 | 327.5 | 6.87 (4.33–10.36) |
| ≥80 years | 51 | 23.5 | 137.9 | 17.06 (10.88–25.49) |
| **Education** |  |  |  |  |
| 0 years | 20 | 6.4 | 70.4 | 9.15 (3.51–19.41) |
| 1–4 years | 101 | 36.5 | 373.1 | 9.77 (6.86–13.50) |
| 5–8 years | 26 | 12.0 | 110.1 | 10.88 (5.62–19.02) |
| >8 years | 32 | 8.3 | 83.8 | 9.89 (4.35–19.26) |

*Abbreviations: CI = confidence interval; IPCW = inverse probability of censoring weighting. IPCW weights yield non-integer event counts and person-years. Expanded model additionally adjusts for depression, hearing loss, and alcoholism.*
