# Supplementary material for: The Cog‐Aging: a 15‐year cohort study of cognition in Brazilian older adults
Source: Alzheimers Dement. 2026 Sep 7;22(9):e71780. doi: 10.1002/alz.71780 (PMC13550517; doi:10.1002/alz.71780)
Supplement: Supplementary file 4 [file ALZ-22-e71780-s001.zip › Sup_table 10_jul21.docx]

**Supplementary Table 10.** Overall cumulative incidence of dementia among CU and MCI individuals, with IPCW-weighted and expanded-model IPCW-weighted sensitivity analyses

| **Category** | **Time**  **(years)** | **Longitudinal participants only** | | | | **All participants, IPCW-weighted** | | | | **Expanded model, IPCW-weighted** | | | |
| --- | --- | --- | --- | --- | --- | --- | --- | --- | --- | --- | --- | --- | --- |
|  |  | **N**  **total** | **Dementia**  **conversions**  **(n)** | **Deaths**  **(n)** | **Cumulative incidence**  **% (95% CI)** | **N**  **total** | **Weighted dementia**  **conversions (n)** | **Weighted**  **deaths (n)** | **Cumulative incidence**  **% (95% CI)** | **N**  **total** | **Weighted dementia**  **conversions (n)** | **Weighted**  **deaths (n)** | **Cumulative incidence**  **% (95% CI)** |
| **CU** | 1 | 119 | 0 | 1 | 0.00% (0.00–0.00%) | 269 | 0.00 | 0.71 | 0.00% (0.00–0.00%) |  |  |  |  |
|  | 3 | 119 | 3 | 2 | 3.12% (0.00–6.60%) | 269 | 2.62 | 1.74 | 2.79% (0.00–5.97%) |  |  |  |  |
|  | 5 | 119 | 7 | 2 | 9.42% (2.62–16.23%) | 269 | 6.67 | 1.74 | 9.40% (2.46–16.35%) |  |  |  |  |
|  | 10 | 119 | 12 | 6 | 20.44% (9.43–31.46%) | 269 | 11.08 | 5.65 | 19.34% (8.70–29.98%) |  |  |  |  |
| **MCI** | 1 | 206 | 15 | 2 | 7.78% (4.00–11.57%) | 370 | 16.58 | 2.40 | 4.47% (2.25–6.69%) | 179 | 9.09 | 1.99 | 6.22% (2.25–10.19%) |
|  | 3 | 206 | 49 | 8 | 29.85% (22.74–36.96%) | 370 | 50.57 | 8.28 | 27.64% (20.50–34.77%) | 179 | 39.05 | 8.20 | 31.01% (22.80–39.21%) |
|  | 5 | 206 | 63 | 14 | 41.24% (33.15–49.33%) | 370 | 64.31 | 14.47 | 39.45% (31.12–47.78%) | 179 | 49.21 | 12.81 | 41.24% (32.16–50.33%) |
|  | 10 | 206 | 78 | 14 | 59.13% (49.68–68.58%) | 370 | 78.83 | 14.47 | 57.98% (48.07–67.90%) | 179 | 58.58 | 12.81 | 55.14% (44.86–65.42%) |

*Abbreviations: CU = cognitively unimpaired; MCI = mild cognitive impairment; CI = confidence interval; IPCW = inverse probability of censoring weighting; NA = not available. The expanded model adjusts for age, sex, education, ≥3 cardiovascular comorbidities, depression, hearing loss, and alcoholism and was estimated only for the MCI group (N = 179); it was not available for the CU group.*
