# Supplementary material for: The Cog‐Aging: a 15‐year cohort study of cognition in Brazilian older adults
Source: Alzheimers Dement. 2026 Sep 7;22(9):e71780. doi: 10.1002/alz.71780 (PMC13550517; doi:10.1002/alz.71780)
Supplement: Supplementary file 4 [file ALZ-22-e71780-s001.zip › Sup_table 11_jul21.docx]

**Supplementary Table 11.** MCI group stratified Cumulative incidence functions by sex, age and education, with IPCW-weighted and expanded-model IPCW-weighted sensitivity analyses

| **Category** | **Time**  **(years)** | **Original analysis: excluding baseline-only participants** | | | | **Sensitivity analysis: baseline-only included and IPCW-weighted** | | | | **Expanded model, IPCW-weighted** | | | |
| --- | --- | --- | --- | --- | --- | --- | --- | --- | --- | --- | --- | --- | --- |
|  |  | **N**  **total** | **Dementia**  **conversions**  **(n)** | **Deaths**  **(n)** | **Cumulative incidence,**  **% (95% CI)** | **N**  **total** | **Weighted dementia**  **conversions (n)** | **Weighted**  **deaths (n)** | **Cumulative incidence**  **% (95% CI)** | **N**  **total** | **Weighted dementia**  **conversions (n)** | **Weighted**  **deaths (n)** | **Cumulative incidence**  **% (95% CI)** |
| **Male** | 1 | 63 | 31 | 6 | 9.99% (4.68–21.35%) | 120 | 32.25 | 7.26 | 5.1% (2.32–11.22%) | 58 | 23.59 | 5.69 | 8.10% (0.48–15.73%) |
|  | 3 | 63 | 31 | 6 | 41.48% (30.03–57.3%) | 120 | 32.25 | 7.26 | 39.78% (28.04–56.45%) | 58 | 23.59 | 5.69 | 42.87% (28.16–57.58%) |
|  | 5 | 63 | 31 | 6 | 50.7% (38.47–66.81%) | 120 | 32.25 | 7.26 | 49.08% (36.41–66.16%) | 58 | 23.59 | 5.69 | 48.26% (33.24–63.29%) |
|  | 10 | 63 | 31 | 6 | 65.62% (52.6–81.87%) | 120 | 32.25 | 7.26 | 63.34% (49.66–80.78%) | 58 | 23.59 | 5.69 | 62.00% (45.97–78.03%) |
| **Female** | 1 | 143 | 52 | 8 | 6.79% (3.62–12.77%) | 250 | 51.79 | 7.21 | 4.14% (2.18–7.84%) | 121 | 39.57 | 7.12 | 5.35% (0.75–9.94%) |
|  | 3 | 143 | 52 | 8 | 24.13% (17.27–33.71%) | 250 | 51.79 | 7.21 | 21.33% (14.87–30.59%) | 121 | 39.57 | 7.12 | 25.03% (15.40–34.65%) |
|  | 5 | 143 | 52 | 8 | 36.51% (27.95–47.69%) | 250 | 51.79 | 7.21 | 34.35% (25.77–45.78%) | 121 | 39.57 | 7.12 | 37.79% (26.46–49.11%) |
|  | 10 | 143 | 52 | 8 | 55.73% (44.78–69.36%) | 250 | 51.79 | 7.21 | 55.08% (43.6–69.58%) | 121 | 39.57 | 7.12 | 51.63% (38.43–64.83%) |
| **60–69 years** | 1 | 47 | 21 | 0 | 11.8% (5.18–26.89%) | 84 | 20.40 | 0 | 6.49% (2.77–15.25%) | 41 | 17.13 | 0 | 8.17% (0.00–17.03%) |
|  | 3 | 47 | 21 | 0 | 35.29% (22.61–55.07%) | 84 | 20.40 | 0 | 33.19% (20.41–53.99%) | 41 | 17.13 | 0 | 34.67% (17.88–51.46%) |
|  | 5 | 47 | 21 | 0 | 42.48% (28.58–63.13%) | 84 | 20.40 | 0 | 40.24% (26.16–61.9%) | 41 | 17.13 | 0 | 43.86% (25.47–62.25%) |
|  | 10 | 47 | 21 | 0 | 60.16% (44.11–82.05%) | 84 | 20.40 | 0 | 56.56% (40.01–79.95%) | 41 | 17.13 | 0 | 57.96% (38.31–77.60%) |
| **70–79 years** | 1 | 105 | 33 | 9 | 5.14% (2.19–12.08%) | 169 | 32.44 | 9.36 | 3.35% (1.42–7.93%) | 87 | 22.51 | 8.97 | 2.67% (0.00–6.33%) |
|  | 3 | 105 | 33 | 9 | 21.67% (14.33–32.77%) | 169 | 32.44 | 9.36 | 19.53% (12.56–30.36%) | 87 | 22.51 | 8.97 | 20.44% (10.40–30.49%) |
|  | 5 | 105 | 33 | 9 | 33.6% (24.09–46.85%) | 169 | 32.44 | 9.36 | 32.09% (22.48–45.81%) | 87 | 22.51 | 8.97 | 28.83% (16.89–40.77%) |
|  | 10 | 105 | 33 | 9 | 49.78% (37.48–66.12%) | 169 | 32.44 | 9.36 | 48.38% (35.92–65.16%) | 87 | 22.51 | 8.97 | 40.91% (26.31–55.51%) |
| **≥80 years** | 1 | 54 | 29 | 5 | 9.55% (4.15–21.99%) | 117 | 31.20 | 5.11 | 4.72% (2.01–11.1%) | 51 | 23.53 | 3.84 | 11.07% (0.72–21.42%) |
|  | 3 | 54 | 29 | 5 | 40.55% (28.21–58.3%) | 117 | 31.20 | 5.11 | 37.48% (25.13–55.91%) | 51 | 23.53 | 3.84 | 47.51% (29.87–65.16%) |
|  | 5 | 54 | 29 | 5 | 55.05% (41.43–73.15%) | 117 | 31.20 | 5.11 | 52.23% (38.22–71.38%) | 51 | 23.53 | 3.84 | 62.85% (45.49–80.22%) |
|  | 10 | 54 | 29 | 5 | 72.98% (59.45–89.59%) | 117 | 31.20 | 5.11 | 72.68% (58.64–90.07%) | 51 | 23.53 | 3.84 | 75.15% (59.20–91.09%) |
| **0 years** | 1 | 27 | 11 | 5 | 0% (0–0%) | 53 | 10.81 | 5.13 | 0% (0–0%) | 20 | 6.44 | 4.84 | 0.00% (0.00–0.00%) |
|  | 3 | 27 | 11 | 5 | 9.53% (2.55–35.61%) | 53 | 10.81 | 5.13 | 9.75% (2.53–37.55%) | 20 | 6.44 | 4.84 | 13.58% (0.00–31.07%) |
|  | 5 | 27 | 11 | 5 | 38.09% (21.14–68.63%) | 53 | 10.81 | 5.13 | 38.78% (21.43–70.16%) | 20 | 6.44 | 4.84 | 38.70% (11.83–65.57%) |
|  | 10 | 27 | 11 | 5 | 66.11% (45.63–95.78%) | 53 | 10.81 | 5.13 | 65.3% (43.39–98.27%) | 20 | 6.44 | 4.84 | 53.38% (23.35–83.41%) |
| **1–4 years** | 1 | 116 | 47 | 8 | 8.32% (4.45–15.56%) | 210 | 47.74 | 8.60 | 4.74% (2.49–9%) | 101 | 36.46 | 6.72 | 8.91% (2.56–15.27%) |
|  | 3 | 116 | 47 | 8 | 30.06% (21.96–41.16%) | 210 | 47.74 | 8.60 | 27.26% (19.31–38.48%) | 101 | 36.46 | 6.72 | 32.06% (21.14–42.97%) |
|  | 5 | 116 | 47 | 8 | 38.19% (29.12–50.07%) | 210 | 47.74 | 8.60 | 35.65% (26.51–47.95%) | 101 | 36.46 | 6.72 | 39.00% (27.37–50.64%) |
|  | 10 | 116 | 47 | 8 | 56.42% (45.62–69.78%) | 210 | 47.74 | 8.60 | 55.98% (44.67–70.16%) | 101 | 36.46 | 6.72 | 54.28% (41.21–67.35%) |
| **5–8 years** | 1 | 30 | 12 | 1 | 3.57% (0.52–24.47%) | 51 | 12.34 | 0.73 | 2.45% (0.36–16.94%) | 26 | 11.98 | 1.25 | 4.66% (0.00–13.57%) |
|  | 3 | 30 | 12 | 1 | 35.07% (20.67–59.52%) | 51 | 12.34 | 0.73 | 35.1% (20.47–60.19%) | 26 | 11.98 | 1.25 | 39.97% (19.54–60.40%) |
|  | 5 | 30 | 12 | 1 | 40.62% (24.89–66.29%) | 51 | 12.34 | 0.73 | 41.14% (25.04–67.58%) | 26 | 11.98 | 1.25 | 47.05% (24.86–69.24%) |
|  | 10 | 30 | 12 | 1 | 49.88% (31.08–80.05%) | 51 | 12.34 | 0.73 | 48.28% (30.08–77.51%) | 26 | 11.98 | 1.25 | 54.67% (31.06–78.28%) |
| **>8 years** | 1 | 33 | 13 | 0 | 15.62% (6.98–34.95%) | 56 | 13.16 | 0 | 9.65% (4.2–22.18%) | 32 | 8.29 | 0 | 3.76% (0.00–11.00%) |
|  | 3 | 33 | 13 | 0 | 43.1% (25.87–71.82%) | 56 | 13.16 | 0 | 41.07% (23.31–72.37%) | 32 | 8.29 | 0 | 31.98% (7.24–56.73%) |
|  | 5 | 33 | 13 | 0 | 60.17% (39.38–91.94%) | 56 | 13.16 | 0 | 58.07% (36.93–91.34%) | 32 | 8.29 | 0 | 54.71% (24.22–85.21%) |
|  | 10 | 33 | 13 | 0 | 70.13% (48.73–100%) | 56 | 13.16 | 0 | 66.48% (44.34–99.68%) | 32 | 8.29 | 0 | 64.80% (34.56–95.04%) |

*Abbreviations: CI = confidence interval; IPCW = inverse probability of censoring weighting. The expanded model adjusts for age, sex, education, ≥3 cardiovascular comorbidities, depression, hearing loss, and alcoholism; strata totals reflect the reduced analytic sample with complete data on these covariates (overall MCI N = 179).*
