# Supplementary material for: The Cog‐Aging: a 15‐year cohort study of cognition in Brazilian older adults
Source: Alzheimers Dement. 2026 Sep 7;22(9):e71780. doi: 10.1002/alz.71780 (PMC13550517; doi:10.1002/alz.71780)
Supplement: Supplementary file 4 [file ALZ-22-e71780-s001.zip › Sup_table 12_jul21.docx]

**Supplementary Table 12.** Univariable Fine–Gray competing-risks regression for incident dementia in CU and MCI groups, with IPCW-weighted and expanded-model IPCW-weighted sensitivity analyses.

| ***Cognitive Unimpaired (CU)*** | | | | | | | | | | | | |
| --- | --- | --- | --- | --- | --- | --- | --- | --- | --- | --- | --- | --- |
| **Variable** | **Original analysis: excluding baseline-only participants** | | | | **IPCW-weighted sensitivity analysis** | | | |  | | | |
|  | **Dementia**  **events** | **Competing**  **deaths** | **SHR (95% CI)** | **P value** | **Dementia**  **events** | **Competing**  **deaths** | **SHR (95% CI)** | **P value** |  |  |  |  |
| Age | 15 | 6 | 1.123 (1.027-1.228) | 0.011* | 13.96 | 5.65 | 1.123 (1.027-1.23) | 0.011* |  |  |  |  |
| Female Sex | 15 | 6 | 0.942 (0.318-2.794) | 0.915 | 13.96 | 5.65 | 1.028 (0.341-3.097) | 0.961 |  |  |  |  |
| Hypertension | 15 | 6 | 0.929 (0.302-2.86) | 0.898 | 13.96 | 5.65 | 0.883 (0.29-2.684) | 0.826 |  |  |  |  |
| Diabetes Mellitus | 15 | 6 | 1.26 (0.372-4.268) | 0.711 | 13.96 | 5.65 | 1.28 (0.373-4.387) | 0.695 |  |  |  |  |
| Dyslipidemia | 15 | 6 | 1.015 (0.379-2.716) | 0.977 | 13.96 | 5.65 | 0.975 (0.358-2.651) | 0.960 |  |  |  |  |
| Depression | 13 | 5 | 2.844 (0.934-8.661) | 0.066 | 11.84 | 4.62 | 3.289 (1.078-10.033) | 0.036* |  |  |  |  |
| Hearing Loss | 13 | 5 | 2.373 (0.801-7.026) | 0.119 | 11.96 | 4.62 | 2.332 (0.775-7.02) | 0.132 |  |  |  |  |
| Smoking | 14 | 6 | 2.096 (0.722-6.083) | 0.173 | 12.92 | 5.65 | 2.006 (0.675-5.964) | 0.210 |  |  |  |  |
| Alcohol Abuse | 14 | 6 | 2.478 (0.758-8.105) | 0.133 | 12.92 | 5.65 | 2.384 (0.725-7.842) | 0.153 |  |  |  |  |
| Visual Impairment | 12 | 4 | 0.796 (0.263-2.411) | 0.686 | 10.8 | 3.91 | 0.928 (0.3-2.873) | 0.897 |  |  |  |  |
| CAD | 14 | 6 | 3.282 (0.964-11.179) | 0.057 | 12.92 | 5.65 | 3.176 (0.913-11.05) | 0.069 |  |  |  |  |
| Atrial fibrillation | 13 | 6 | 18.303 (1.183-283.112) | 0.038* | 12.02 | 5.65 | 16.495 (0.99-274.73) | 0.051 |  |  |  |  |
| Obesity | 2 | 0 |  |  | 1.94 | 0 |  |  |  |  |  |  |
| Between 1 and 4 years of education | 15 | 6 | 0.71 (0.172-2.939) | 0.637 | 13.96 | 5.65 | 0.674 (0.164-2.764) | 0.583 |  |  |  |  |
| Between 5 and 8 years of education | 15 | 6 | 1.047 (0.228-4.813) | 0.953 | 13.96 | 5.65 | 0.92 (0.197-4.305) | 0.916 |  |  |  |  |
| Over 8 years of education | 15 | 6 | 0.211 (0.022-1.974) | 0.172 | 13.96 | 5.65 | 0.214 (0.023-1.961) | 0.172 |  |  |  |  |
| ***Mild Cognitive Impairment (MCI)*** | | | | | | | | | | | | |
| **Variable** | **Original analysis: excluding baseline-only participants** | | | | **IPCW-weighted sensitivity analysis** | | | | **Expanded model, IPCW-weighted** | | | |
|  | **Dementia**  **events** | **Competing**  **deaths** | **SHR (95% CI)** | **P value** | **Dementia**  **events** | **Competing**  **deaths** | **SHR (95% CI)** | **P value** | **Dementia**  **events** | **Competing**  **deaths** | **SHR (95% CI)** | **P value** |
| Age | 83 | 14 | 1.026 (0.99-1.064) | 0.162 | 84.04 | 14.47 | 1.026 (0.99-1.065) | 0.162 | 63.17 | 12.81 | 1.034 (0.990-1.079) | 0.132 |
| Female Sex | 83 | 14 | 0.724 (0.462-1.133) | 0.157 | 84.04 | 14.47 | 0.751 (0.476-1.186) | 0.219 | 23.6 | 5.69 | — | — |
| Hypertension | 81 | 14 | 1.132 (0.685-1.87) | 0.628 | 81.81 | 14.47 | 1.206 (0.734-1.982) | 0.459 | 63.17 | 12.81 | 1.302 (0.718-2.362) | 0.385 |
| Diabetes Mellitus | 80 | 14 | 0.975 (0.627-1.515) | 0.909 | 80.64 | 14.47 | 0.948 (0.606-1.483) | 0.816 | 63.17 | 12.81 | 0.851 (0.512-1.413) | 0.533 |
| Dyslipidemia | 81 | 14 | 1.108 (0.724-1.695) | 0.638 | 81.81 | 14.47 | 1.136 (0.743-1.738) | 0.556 | 63.17 | 12.81 | 1.225 (0.751-1.999) | 0.416 |
| Depression | 74 | 14 | 1.055 (0.679-1.639) | 0.810 | 74.97 | 14.47 | 1.055 (0.675-1.65) | 0.814 | 63.17 | 12.81 | 1.266 (0.789-2.031) | 0.329 |
| Hearing Loss | 71 | 13 | 0.937 (0.596-1.473) | 0.778 | 71.08 | 13.32 | 1.034 (0.653-1.638) | 0.886 | 63.17 | 12.81 | 1.039 (0.646-1.671) | 0.875 |
| Smoking | 77 | 14 | 1.073 (0.66-1.743) | 0.777 | 77.37 | 14.47 | 1.09 (0.669-1.776) | 0.730 | 63.17 | 12.81 | 1.063 (0.611-1.848) | 0.830 |
| Alcohol Abuse | 77 | 14 | 1.102 (0.713-1.703) | 0.662 | 77.37 | 14.47 | 1.103 (0.709-1.715) | 0.664 | 63.17 | 12.81 | 1.042 (0.639-1.698) | 0.870 |
| Visual Impairment | 69 | 14 | 1.019 (0.635-1.634) | 0.939 | 68.75 | 14.47 | 1.024 (0.634-1.653) | 0.924 | 63.17 | 12.81 | 1.154 (0.700-1.902) | 0.574 |
| Stroke | 82 | 14 | 1.2 (0.526-2.737) | 0.664 | 82.9 | 14.47 | 1.063 (0.443-2.547) | 0.892 |  |  |  |  |
| CAD | 81 | 14 | 0.734 (0.328-1.645) | 0.453 | 81.72 | 14.47 | 0.821 (0.35-1.926) | 0.651 |  |  |  |  |
| Atrial Fibrillation | 73 | 12 | 0.2 (0.025-1.613) | 0.131 | 73.02 | 12.59 | 0.211 (0.026-1.7) | 0.144 |  |  |  |  |
| Obesity | 68 | 12 | 0.85 (0.463-1.56) | 0.599 | 67.88 | 12.5 | 0.751 (0.392-1.441) | 0.389 | 50.7 | 11.93 | 1.156 (0.589-2.270) | 0.673 |
| Between 1 and 4 years of education | 83 | 14 | 1.228 (0.713-2.115) | 0.458 | 84.04 | 14.47 | 1.31 (0.748-2.293) | 0.345 | 63.17 | 12.81 | 1.542 (0.763-3.115) | 0.228 |
| Between 5 and 8 years of education | 83 | 14 | 1.219 (0.587-2.53) | 0.596 | 84.04 | 14.47 | 1.336 (0.63-2.835) | 0.450 | 63.17 | 12.81 | 1.730 (0.741-4.040) | 0.205 |
| Over 8 years of education | 83 | 14 | 2.113 (1.013-4.404) | 0.046* | 84.04 | 14.47 | 2.308 (1.073-4.964) | 0.032* | 63.17 | 12.81 | 1.840 (0.718-4.713) | 0.204 |

*Footnotes: *p-value<0.05. SHR = subdistribution hazard ratio; CI = confidence interval; IPCW = inverse probability of censoring weighting; CAD = coronary artery disease; NA = not available; — = model not estimable (predictor had no variation in the expanded-model complete-case sample). The expanded model adjusts for age, sex, education, ≥3 cardiovascular comorbidities, depression, hearing loss, and alcoholism, and was estimated only for the MCI group; it was not available for the CU group. Stroke, CAD, and Atrial Fibrillation were not modeled individually in the expanded model, as they are captured jointly by the ≥3 cardiovascular comorbidities model.*
