# Supplementary material for: The Cog‐Aging: a 15‐year cohort study of cognition in Brazilian older adults
Source: Alzheimers Dement. 2026 Sep 7;22(9):e71780. doi: 10.1002/alz.71780 (PMC13550517; doi:10.1002/alz.71780)
Supplement: Supplementary file 4 [file ALZ-22-e71780-s001.zip › Sup_table 13_jul21.docx]

**Supplementary table 13.** Fine Gray Multivariable model for CU and MCI groups considering sex, age and education, with IPCW-weighted and expanded-model IPCW-weighted sensitivity analyses.

|  | **Excluding IPCW** | | |  | **Including IPCW** | | |  | **Expanded model, IPCW-weighted** | | | |
| --- | --- | --- | --- | --- | --- | --- | --- | --- | --- | --- | --- | --- |
| **Predictor** | **Dementia events** | **Competing deaths** | **p value** | **SHR (95% CI)** | **Dementia events** | **Competing deaths** | **p value** | **SHR (95% CI)** | **Dementia events** | **Competing deaths** | **P value** | **SHR**  **(95% CI)** |
| ***CU*** |  |  |  |  |  |  |  |  |  |  |  |  |
| Age | 15 | 6 | 0.030^*^ | 1.141 (1.013-1.285) | 13.96 | 5.65 | 0.032^*^ | 1.136 (1.011-1.276) |  |  |  |  |
| Sex | 15 | 6 | 0.609 | 0.694 (0.171-2.814) | 13.96 | 5.65 | 0.672 | 0.746 (0.192-2.897) |  |  |  |  |
| Education  (1-4 yrs.) | 15 | 6 | 0.609 | 0.608 (0.091-4.086) | 13.96 | 5.65 | 0.631 | 0.642 (0.106-3.904) |  |  |  |  |
| Education  (5-8 yrs.) | 15 | 6 | 0.483 | 1.729 (0.375-7.983) | 13.96 | 5.65 | 0.571 | 1.575 (0.327-7.587) |  |  |  |  |
| Education  (>8 yrs.) | 15 | 6 | 0.319 | 0.276 (0.022-3.466) | 13.96 | 5.65 | 0.345 | 0.297 (0.024-3.689) |  |  |  |  |
| ***MCI*** |  |  |  |  |  |  |  |  |  |  |  |  |
| Age | 83 | 14 | 0.081 | 1.034 (0.996-1.074) | 84.04 | 14.47 | 0.084 | 1.034 (0.995-1.075) | 64 | 13 | 0.381 | 1.019 (0.976 - 1.064) |
| Sex | 83 | 14 | 0.301 | 0.785 (0.496-1.243) | 84.04 | 14.47 | 0.424 | 0.826 (0.518-1.318) | 64 | 13 | 0.118 | 0.667 (0.402 - 1.108) |
| Education  (1-4 yrs.) | 83 | 14 | 0.434 | 1.254 (0.71-2.215) | 84.04 | 14.47 | 0.335 | 1.329 (0.745-2.373) | 64 | 13 | 0.617 | 1.199 (0.589 - 2.439) |
| Education  (5-8 yrs.) | 83 | 14 | 0.432 | 1.365 (0.628-2.968) | 84.04 | 14.47 | 0.314 | 1.504 (0.68-3.328) | 64 | 13 | 0.324 | 1.554 (0.647 - 3.732) |
| Education  (>8 yrs.) | 83 | 14 | 0.044^*^ | 2.279 (1.021-5.088) | 84.04 | 14.47 | 0.026^*^ | 2.515 (1.114-5.678) | 64 | 13 | 0.881 | 1.076 (0.410 - 2.828) |

Abbreviations: CU = cognitively unimpaired; MCI = mild cognitive impairment; SHR = subdistribution hazard ratio; CI = confidence interval; IPCW = inverse probability of censoring weighting; yrs.: years. NA = not available; The expanded model adjusts for age, sex, education, ≥3 cardiovascular comorbidities, depression, hearing loss, and alcoholism, and was estimated only for the MCI group; it was not available for the CU group. *p<0.05.
