# Supplementary material for: The Cog‐Aging: a 15‐year cohort study of cognition in Brazilian older adults
Source: Alzheimers Dement. 2026 Sep 7;22(9):e71780. doi: 10.1002/alz.71780 (PMC13550517; doi:10.1002/alz.71780)
Supplement: Supplementary file 4 [file ALZ-22-e71780-s001.zip › Sup_table 14_jul21.docx]

| **Supplementary Table 14.** Comparison of baseline characteristics between participants with and without available APOE genotyping | | | | | | |  |
| --- | --- | --- | --- | --- | --- | --- | --- |
| **Variable** | **With APOE, n (%)** | | **Without APOE, n (%)** | | | **p-value^*^** | |
| **Sex** |  | |  | | | 0.669 | |
| Male | 54 (31.40) | | 10 (27.78) | | |  | |
| Female | 118 (68.60) | | 26 (72.22) | | |  | |
| **Education** |  | |  | | | 0.796 | |
| 0 years | 24 (13.95) | | 3 (8.82) | | |  | |
| 1-4 years | 94 (54.65) | | 22 (64.71) | | |  | |
| 5-8 years | 26 (15.12) | | 4 (11.76) | | |  | |
| >8 years | 28 (16.28) | | 5 (14.71) | | |  | |
| **Hypertension** | 126 (74.56) | | 25 (71.43) | | | 0.701 | |
| **Diabetes mellitus** | 51 (30.36) | | 12 (35.29) | | | 0.571 | |
| **Dyslipidemia** | 84 (50.30) | | 15 (45.45) | | | 0.611 | |
| **Depression** | 61 (38.61) | | 26 (74.29) | | | <0.01^†^ | |
| **Hearing loss** | 52 (34.90) | | 5 (29.41) | | | 0.856 | |
| **Obesity** | 32 (22.22) | | 8 (28.57) | | | 0.479 | |
| **Smoking** | 50 (30.49) | | 4 (26.67) | | | 0.999 | |
| **Alcohol abuse** | 71 (43.29) | | 3 (20.00) | | | 0.139 | |
| **Visual impairment** | 53 (35.81) | | 8 (47.06) | | | 0.519 | |
| **Stroke** | 12 (7.10) | | 2 (5.71) | | | 0.999 | |
| **Coronary artery disease** | 15 (8.88) | | 2 (11.11) | | | 0.670 | |
| **Atrial fibrillation** | 9 (6.04) | | 0 (0.00) | | | 0.999 | |

Footnotes: ^*^ p-value considered for Chi-square or Fisher Exact as appropriate for each variable; ^†^ p-value<0.05.
