# Supplementary material for: The Cog‐Aging: a 15‐year cohort study of cognition in Brazilian older adults
Source: Alzheimers Dement. 2026 Sep 7;22(9):e71780. doi: 10.1002/alz.71780 (PMC13550517; doi:10.1002/alz.71780)
Supplement: Supplementary file 4 [file ALZ-22-e71780-s001.zip › Sup_table 15_jul21.docx]

**Supplementary Table 15.** Comparison of demographic and clinical characteristics between APOE ε4 non-carriers and carriers.

| **Variable** | **APOE** ε4 non-carriers  **n (%)** | **APOE** ε4 carriers  **n (%)** | **p-value^*^** |
| --- | --- | --- | --- |
| **Sex** |  |  | 0.975 |
| Male | 33 (30.84) | 21 (32.31) |  |
| Female | 74 (69.16) | 44 (67.69) |  |
|  |  |  |  |
| **Education** |  |  |  |
| 0 years | 17 (15.89) | 7 (10.77) | 0.480 |
| 1-4 years | 59 (55.14) | 35 (53.85) |  |
| 5-8 years | 13 (12.15) | 13 (20.00) |  |
| >8 years | 18 (16.82) | 10 (15.38) |  |
| **Hypertension** | 81 (76.42) | 45 (71.43) | 0.591 |
| **Diabetes mellitus** | 33 (31.13) | 18 (29.03) | 0.911 |
| **Dyslipidemia** | 54 (50.94) | 30 (49.18) | 0.953 |
| **Depression** | 41 (41.41) | 20 (33.90) | 0.442 |
| **Hearing loss** | 34 (35.79) | 18 (33.33) | 0.902 |
| **Obesity** | 22 (24.72) | 10 (18.18) | 0.442 |
| **Smoking** | 31 (29.81) | 19 (31.67) | 0.942 |
| **Alcohol Abuse** | 42 (40.38) | 29 (48.33) | 0.409 |
| **Visual impairment** | 32 (34.04) | 21 (38.89) | 0.679 |
| **Stroke** | 8 (7.55) | 4 (6.35) | 0.999 |
| **Coronary artery disease** | 8 (7.55) | 7 (11.11) | 0.611 |
| **Atrial fibrillation** | 7 (7.29) | 2 (3.77) | 0.492 |

Abbreviations: APOE ε4 carriers = ε4 allele of the apolipoprotein 4 gene; Footnotes: * p-value for Chi-square test.
