# Supplementary material for: The Cog‐Aging: a 15‐year cohort study of cognition in Brazilian older adults
Source: Alzheimers Dement. 2026 Sep 7;22(9):e71780. doi: 10.1002/alz.71780 (PMC13550517; doi:10.1002/alz.71780)
Supplement: Supplementary file 4 [file ALZ-22-e71780-s001.zip › Sup_table 16_jul21.docx]

| **Supplementary table 16.** Fine Gray Multivariable model including ApoE ε4 | | | | | | | | | |
| --- | --- | --- | --- | --- | --- | --- | --- | --- | --- |
| **Variable** | | | | **N** | **Dementia Conversions** | **Death** | **SHR** | **95% CI** | **p-value** |
| Age | | | | 172 | 78 | 14 | 1.03 | 0.99 – 1.07 | 0.109 |
| Sex (female vs male) | | | | 172 | 78 | 14 | 0.74 | 0.46 – 1.19 | 0.210 |
| Education: 1-4 years (vs 0 years) | | | | 172 | 78 | 14 | 1.15 | 0.65 – 2.04 | 0.629 |
| Education: 5-8 years (vs 0 years) | | | | 172 | 78 | 14 | 1.34 | 0.62 – 2.92 | 0.458 |
| Education: >8 years (vs 0 years) | | | | 172 | 78 | 14 | 2.27 | 1.00 – 5.13 | 0.049^*^ |
| APOE ε4 carrier (yes vs no) | | | | 172 | 78 | 14 | 1.30 | 0.84 – 2.01 | 0.236 |

*Abbreviations:* APOE ε4 carriers = ε4 allele of the apolipoprotein 4 gene. SHR = subdistribution hazard ratio; CI = confidence interval. Footnote: ^*^p<0.05
