# Supplementary material for: The Cog‐Aging: a 15‐year cohort study of cognition in Brazilian older adults
Source: Alzheimers Dement. 2026 Sep 7;22(9):e71780. doi: 10.1002/alz.71780 (PMC13550517; doi:10.1002/alz.71780)
Supplement: Supplementary file 4 [file ALZ-22-e71780-s001.zip › Sup_table 17_jul21.docx]

| **Supplementary Table 17.** APOE imputation model and extent of missing data | | | | | | |
| --- | --- | --- | --- | --- | --- | --- |
| ***Imputation model specification*** |  |  |  |  |  | |
| **Component** | **Specification** |  |  |  |  | |
| Substantive model | Fine–Gray model including age, sex, education, and APOE ε4 carriers status | | |  |  | |
| Outcome structure | Follow-up time, dementia event indicator, and competing death status incorporated through smcfcs.finegray() | | | | | |
| Auxiliary variables | Family history of dementia, dyslipidemia, obesity, depression, hypertension, diabetes mellitus, and recruitment year | | | | | |
| Number of imputations | 50 |  |  |  |  | |
| Iterations per chain | 30 |  |  |  |  | |
| Binary imputation method | Bias-reduced logistic regression | |  |  |  | |
| Education imputation method | Multinomial logistic regression |  |  |  |  | |
|  |  |  |  |  |  | |
| ***Extent and pattern of missing data*** |  |  |  |  |  | |
| **Variable** | **Missing, n** | **Missing, %** | **Available among participants with missing APOE, n/36** | **Missing if APOE observed, %** | **Missing if APOE missing, %** | |
| Education | 2 | 0.96 | 34/36 | 0.00 | 5.56 | |
| APOE carrier status | 36 | 17.31 | — | — | — | |
| Family history of dementia | 23 | 11.06 | 18/36 | 2.91 | 50.00 | |
| Dyslipidemia | 8 | 3.85 | 33/36 | 2.91 | 8.33 | |
| Obesity | 37 | 17.79 | 28/36 | 16.86 | 22.22 | |
| Depression | 15 | 7.21 | 35/36 | 8.14 | 2.78 | |
| Hypertension | 4 | 1.92 | 35/36 | 1.74 | 2.78 | |
| Diabetes mellitus | 6 | 2.88 | 34/36 | 2.33 | 5.56 | |
| Age | 0 | 0.00 | 36/36 | 0.00 | 0.00 | |
| Sex | 0 | 0.00 | 36/36 | 0.00 | 0.00 | |
| Recruitment year | 0 | 0.00 | 36/36 | 0.00 | 0.00 | |
| Footnotes: The analysis included 208 participants: 110 censored observations, 84 dementia events, and 14 competing deaths. APOE was observed for 172 participants and missing for 36. Recruitment year was complete and ranged from 2010 to 2025. The final parsimonious imputation model excluded highly incomplete or sparse auxiliary variables that produced numerical instability. | | | | | |  |
