# Supplementary material for: The Cog‐Aging: a 15‐year cohort study of cognition in Brazilian older adults
Source: Alzheimers Dement. 2026 Sep 7;22(9):e71780. doi: 10.1002/alz.71780 (PMC13550517; doi:10.1002/alz.71780)
Supplement: Supplementary file 4 [file ALZ-22-e71780-s001.zip › Sup_table 18_jul21.docx]

| **Supplementary Table 18.** Convergence and imputation diagnostics | | |
| --- | --- | --- |
|  |  |  |
| **Diagnostic** | **Estimate** | **Interpretation** |
| Number of imputations | 50 | Adequate for the estimated missing information. |
| Iterations per chain | 30 | Used for assessment of coefficient stability. |
| Standardized drift across substantive-model coefficients | 0.033–0.106 | No marked systematic drift across the 30 iterations. |
| Observed prevalence of APOE ε4 carriers | 37.8% | Among 172 participants with observed APOE genotype. |
| Mean imputed carrier prevalence | 44.6% | Among the 36 participants with missing APOE genotype. |
| Imputed carrier prevalence, median (IQR) | 44.4% (41.7–47.2) | Shows between-imputation variability. |
| Imputed carrier prevalence, overall range | 33.3–61.1% | No deterministic assignment to a single category. |
| SD of imputed carrier prevalence | 5.8 percentage points | Variation across the 50 completed datasets. |
| **Fraction of missing information for APOE coefficient** | **12.8%** | **Estimated using Rubin’s rules.** |
| Relative efficiency with 50 imputations | 99.7% | Indicates sufficient number of imputations. |
| Internal numerical warnings | Present in some binary conditional models | Consistent with sparse categories; analysis retained as exploratory. |
| *Abbreviations:* APOE ε4 carriers = ε4 allele of the apolipoprotein 4 gene. Visual inspection of the full trace plots showed that the substantive-model coefficient trajectories fluctuated around stable levels without marked systematic drift. Greater variability was observed for some education categories, consistent with their smaller sample sizes and wider uncertainty. The complete trace plots should be presented as a Supplementary Figure. | | |
|  | | |
