# Supplementary material for: The Cog‐Aging: a 15‐year cohort study of cognition in Brazilian older adults
Source: Alzheimers Dement. 2026 Sep 7;22(9):e71780. doi: 10.1002/alz.71780 (PMC13550517; doi:10.1002/alz.71780)
Supplement: Supplementary file 4 [file ALZ-22-e71780-s001.zip › Sup_table 19_jul21.docx]

| **Supplementary Table 19.** Complete-case and multiply imputed Fine–Gray models | | | | | | |  |  |  |  |  |  |  |  |  |  |
| --- | --- | --- | --- | --- | --- | --- | --- | --- | --- | --- | --- | --- | --- | --- | --- | --- |
| **Covariate** | **Complete-case SHR** | **95% CI** | **p-value** | **Multiple-imputation SHR** | **95% CI** | **p-value** |  |  |  |  |  |  |  |  |  |  |
| Age, per year | 1.03 | 0.99–1.07 | 0.109 | 1.04 | 1.00–1.08 | 0.035 |  |  |  |  |  |  |  |  |  |  |
| Male sex | 1.35 | 0.84–2.17 | 0.210 | 1.32 | 0.83–2.11 | 0.240 |  |  |  |  |  |  |  |  |  |  |
| Education: 1–4 years vs 0 | 1.15 | 0.65–2.05 | 0.629 | 1.17 | 0.59–2.31 | 0.656 |  |  |  |  |  |  |  |  |  |  |
| Education: 5–8 years vs 0 | 1.34 | 0.62–2.92 | 0.458 | 1.24 | 0.53–2.92 | 0.623 |  |  |  |  |  |  |  |  |  |  |
| Education: >8 years vs 0 | 2.27 | 1.00–5.12 | 0.049 | 2.24 | 0.95–5.29 | 0.067 |  |  |  |  |  |  |  |  |  |  |
| APOE ε4 carriers | 1.30 | 0.84–2.01 | 0.236 | 1.35 | 0.83–2.18 | 0.222 |  |  |  |  |  |  |  |  |  |  |

*Abbreviations:* APOE ε4 carriers = ε4 allele of the apolipoprotein 4 gene. *Complete-case analysis: N=172, dementia events=78, competing deaths=14. Multiple-imputation analysis: N=208, dementia events=84, competing deaths=14. SHR denotes subdistribution hazard ratio. Estimates from the 50 completed datasets were combined using Rubin’s rules.*
