# Supplementary material for: The Cog‐Aging: a 15‐year cohort study of cognition in Brazilian older adults
Source: Alzheimers Dement. 2026 Sep 7;22(9):e71780. doi: 10.1002/alz.71780 (PMC13550517; doi:10.1002/alz.71780)
Supplement: Supplementary file 4 [file ALZ-22-e71780-s001.zip › Sup_table 20_jul21.docx]

| **Supplementary Table 20.** Rubin’s-rule diagnostics for the multiply imputed Fine–Gray model | | | |
| --- | --- | --- | --- |
| **Covariate** | **Relative increase in variance** | **Fraction of missing information** | **Relative efficiency** |
| Age | 0.0246 | 0.0240 | 0.9995 |
| Male sex | 0.0394 | 0.0379 | 0.9992 |
| Education: 1–4 years vs 0 | 0.0360 | 0.0347 | 0.9993 |
| Education: 5–8 years vs 0 | 0.0282 | 0.0274 | 0.9995 |
| Education: >8 years vs 0 | 0.0265 | 0.0258 | 0.9995 |
| APOE ε4 carriers | 0.1467 | 0.1280 | 0.9974 |
| *Abbreviations:* APOE ε4 carriers = ε4 allele of the apolipoprotein 4 gene. The APOE carrier coefficient had the largest fraction of missing information, as expected because APOE was the principal incompletely observed covariate. Relative efficiency remained above 99.7% for all coefficients. | | | |
|  | | | |
