# Supplementary material for: The Cog‐Aging: a 15‐year cohort study of cognition in Brazilian older adults
Source: Alzheimers Dement. 2026 Sep 7;22(9):e71780. doi: 10.1002/alz.71780 (PMC13550517; doi:10.1002/alz.71780)
Supplement: Supplementary file 4 [file ALZ-22-e71780-s001.zip › Sup_table 21_jul21.docx]

**Supplementary Table 21.** Recruitment-era sensitivity analysis: Fine–Gray competing-risks regression for incident dementia in the MCI group, by follow-up horizon and model

| **Follow-up horizon** | **Model** | **N** | **Dementia events** | **Deaths** | **Variable** | **SHR (95% CI)** | **P value** |
| --- | --- | --- | --- | --- | --- | --- | --- |
| **Full follow-up** | Recruitment era adjusted model | 205 | 83 | 14 | Age | 1.037 (0.999-1.077) | 0.055 |
|  |  |  |  |  | Sex | 0.888 (0.557-1.414) | 0.617 |
|  |  |  |  |  | Education Category 1-4 years | 1.319 (0.730-2.383) | 0.358 |
|  |  |  |  |  | Education Category 5-8 years | 1.509 (0.686-3.321) | 0.307 |
|  |  |  |  |  | Education Category >8 years | 1.990 (0.864-4.580) | 0.106 |
|  |  |  |  |  | Recruitment Era 2015-2019 | 1.720 (1.017-2.908) | **0.043*** |
|  |  |  |  |  | Recruitment Era 2020 onward | 3.129 (1.593-6.144) | **<0.001*** |

*Abbreviations:* SHR = subdistribution hazard ratio; CI = confidence interval.*p<0.05
