# Supplementary material for: The Cog‐Aging: a 15‐year cohort study of cognition in Brazilian older adults
Source: Alzheimers Dement. 2026 Sep 7;22(9):e71780. doi: 10.1002/alz.71780 (PMC13550517; doi:10.1002/alz.71780)
Supplement: Supplementary file 4 [file ALZ-22-e71780-s001.zip › Sup_table 22_jul21.docx]

| **Supplementary Table 22.** Recruitment-era sensitivity analysis: Sociodemographic characteristics of each era. | | | | | | |  |
| --- | --- | --- | --- | --- | --- | --- | --- |
| **Variable** | **Up to 2014** | | **2015-2019** | | | **2020 onward** | |
| **Age** | 75.38 (7.03) | | 75.6 (6.57) | | | 73.43 (6.51) | |
| **Sex** |  | |  | | |  | |
| Male | 33 (25.78%) | | 12 (34.29%) | | | 18 (42.86%) | |
| Female | 95 (74.22%) | | 23 (65.71%) | | | 24 (57.14%) | |
| **Education** |  | |  | | |  | |
| 0 years | 17 (13.28%) | | 8 (22.86%) | | | 2 (4.76%) | |
| 1-4 years | 77 (60.16%) | | 20 (57.14%) | | | 18 (42.86%) | |
| 5-8 years | 23 (17.97%) | | 2 (5.71%) | | | 5 (11.90%) | |
| >8 years | 11 (8.59%) | | 5 (14.29%) | | | 17 (40.48%) | |

*Footnotes:* Mean (Standard Deviation).
