# Supplementary material for: The Cog‐Aging: a 15‐year cohort study of cognition in Brazilian older adults
Source: Alzheimers Dement. 2026 Sep 7;22(9):e71780. doi: 10.1002/alz.71780 (PMC13550517; doi:10.1002/alz.71780)
Supplement: Supplementary file 5 [file ALZ-22-e71780-s002.zip › Sup_fig1_jul21.pdf]

Supplementary Figure 1. Flowchart of referral to the Cog-Aging.

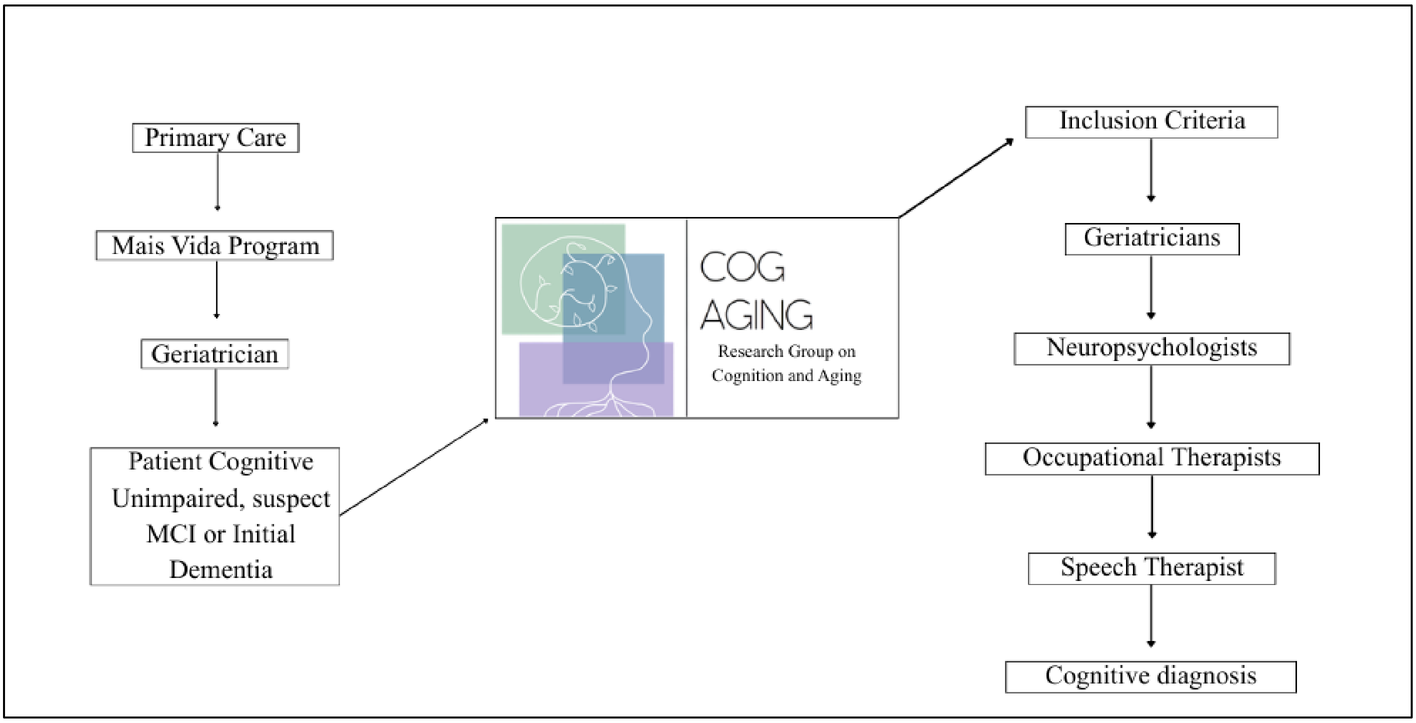

Abbreviations: Mild Cognitive Impairment (MCI); Research Group on Cognition and Aging (Cog-Aging).
