# Supplementary material for: The Cog‐Aging: a 15‐year cohort study of cognition in Brazilian older adults
Source: Alzheimers Dement. 2026 Sep 7;22(9):e71780. doi: 10.1002/alz.71780 (PMC13550517; doi:10.1002/alz.71780)
Supplement: Supplementary file 5 [file ALZ-22-e71780-s002.zip › Sup_fig2_jul21.pdf]

Supplementary Figure 2. Evolution of the Cog-Aging protocol.

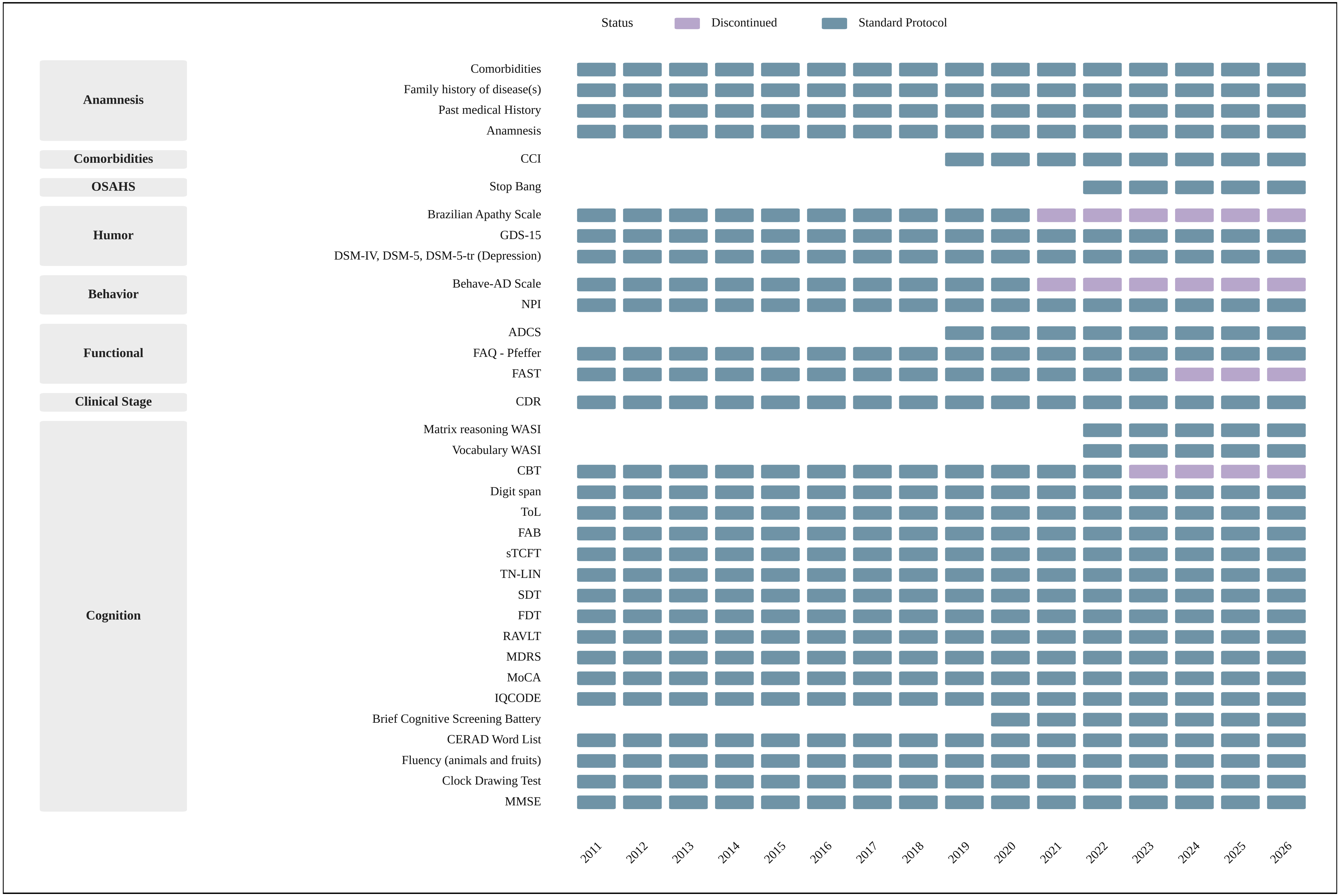

Abbreviations: ADCS, Alzheimer's Disease Cooperative Study–ADL; CBT, Corsi Block-Tapping Test; CCI, Charlson Comorbidity Index; CDR, Clinical Dementia Rating; DSM, Diagnostic and Statistical Manual of Mental Disorders; FAB, Frontal Assessment Battery; FAST, Functional Assessment Staging Tool; FDT, Five Digit Test; FAQ, Functional Activities Questionnaire (Pfeffer); GDS-15, Geriatric Depression Scale; IQCODE, Informant Questionnaire on Cognitive Decline; MDRS, Mattis Dementia Rating Scale; MMSE, Mini-Mental State Examination; MoCA, Montreal Cognitive Assessment; NPI, Neuropsychiatric Inventory; OSAHS, Obstructive Sleep Apnea–Hypopnea Syndrome (STOP-Bang); RAVLT, Rey Auditory Verbal Learning Test; SDT, Stick Design Test; sTCFT, Simplified Taylor Complex Figure Test; TN-LIN, Neuropsychological Investigations Laboratory Naming Test; ToL, Tower of London; WASI, Wechsler Abbreviated Scale of Intelligence.
