# Supplementary material for: The Cog‐Aging: a 15‐year cohort study of cognition in Brazilian older adults
Source: Alzheimers Dement. 2026 Sep 7;22(9):e71780. doi: 10.1002/alz.71780 (PMC13550517; doi:10.1002/alz.71780)
Supplement: Supplementary file 5 [file ALZ-22-e71780-s002.zip › Sup_fig3_jul21.pdf]

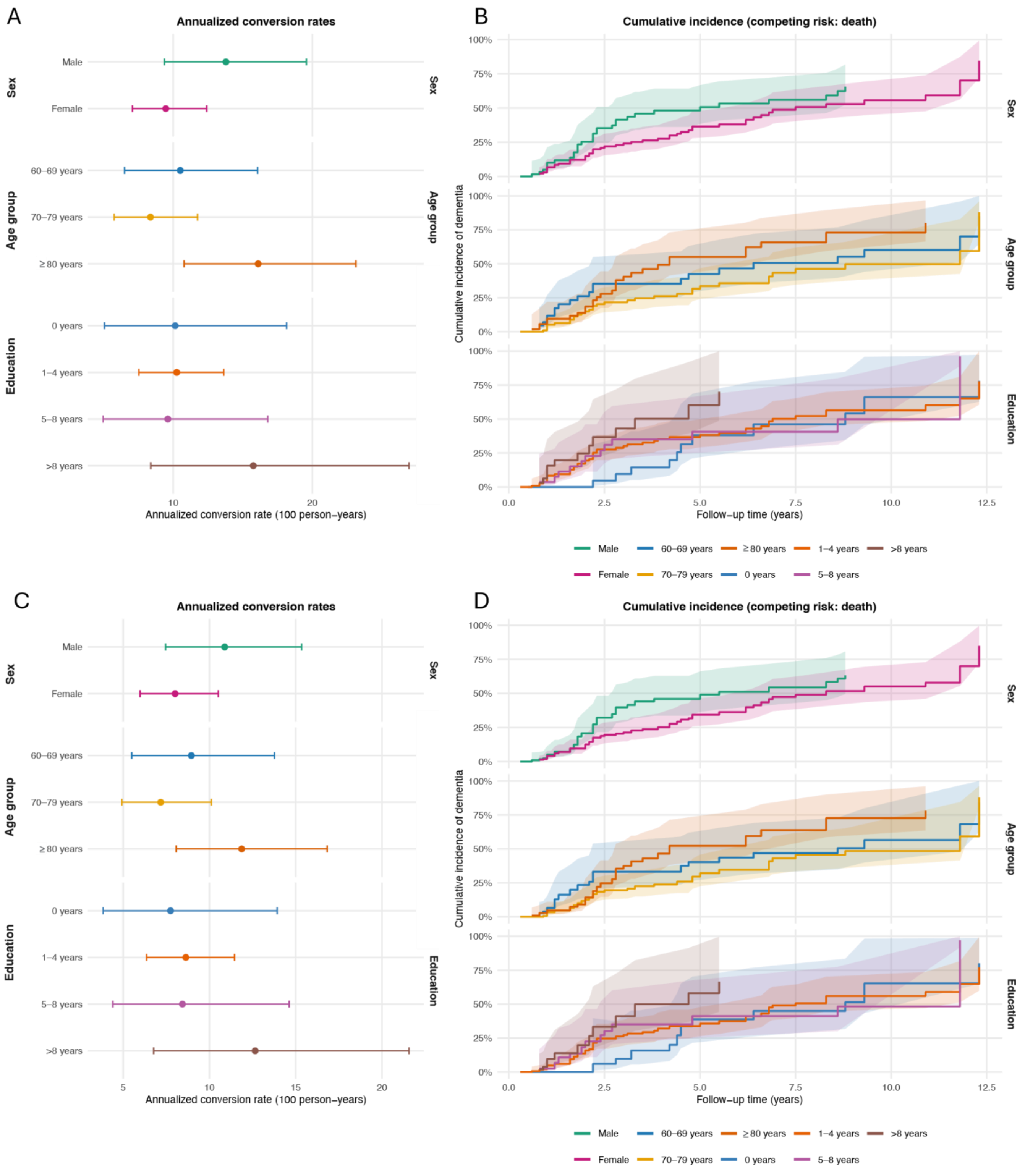
A
Annualized conversion rates
Sex
Male
Female
Age group
60–69 years
70–79 years
≥80 years
Education
0 years
1–4 years
5–8 years
>8 years
10
20
Annualized conversion rate (100 person–years)
B
Cumulative incidence (competing risk: death)
Cumulative incidence of dementia
0%
25%
50%
75%
100%
0.0
2.5
5.0
7.5
10.0
12.5
Follow-up time (years)
Male
Female
60–69 years
70–79 years
≥80 years
0 years
1–4 years
5–8 years
>8 years
C
Annualized conversion rates
Sex
Male
Female
Age group
60–69 years
70–79 years
≥80 years
Education
0 years
1–4 years
5–8 years
>8 years
5
10
15
20
Annualized conversion rate (100 person–years)
D
Cumulative incidence (competing risk: death)
Cumulative incidence of dementia
0%
25%
50%
75%
100%
0.0
2.5
5.0
7.5
10.0
12.5
Follow-up time (years)
Male
Female
60–69 years
70–79 years
≥80 years
0 years
1–4 years
5–8 years
>8 years

Supplementary Figure 3. Unweighted and inverse probability-of-censoring weighting (IPCW) adjusted annualized MCI-to-dementia conversion rates and cumulative incidence functions, stratified by sex, age group, and education. Panels A and C show annualized conversion rates per 100 person-years with 95% confidence intervals, whereas Panels B and D show cumulative incidence functions for conversion to dementia, accounting for death as a competing event. Panels A and B present conventional unweighted estimates, while Panels C and D present estimates adjusted using inverse probability-of-censoring weights to account for potentially informative loss to follow-up. Age groups were defined as 60–69, 70–79, and ≥80 years, and education categories as 0, 1–4, 5–8, and >8 years.
