# Supplementary material for: The Cog‐Aging: a 15‐year cohort study of cognition in Brazilian older adults
Source: Alzheimers Dement. 2026 Sep 7;22(9):e71780. doi: 10.1002/alz.71780 (PMC13550517; doi:10.1002/alz.71780)
Supplement: Supplementary file 5 [file ALZ-22-e71780-s002.zip › Sup_fig4_jul21.pdf]

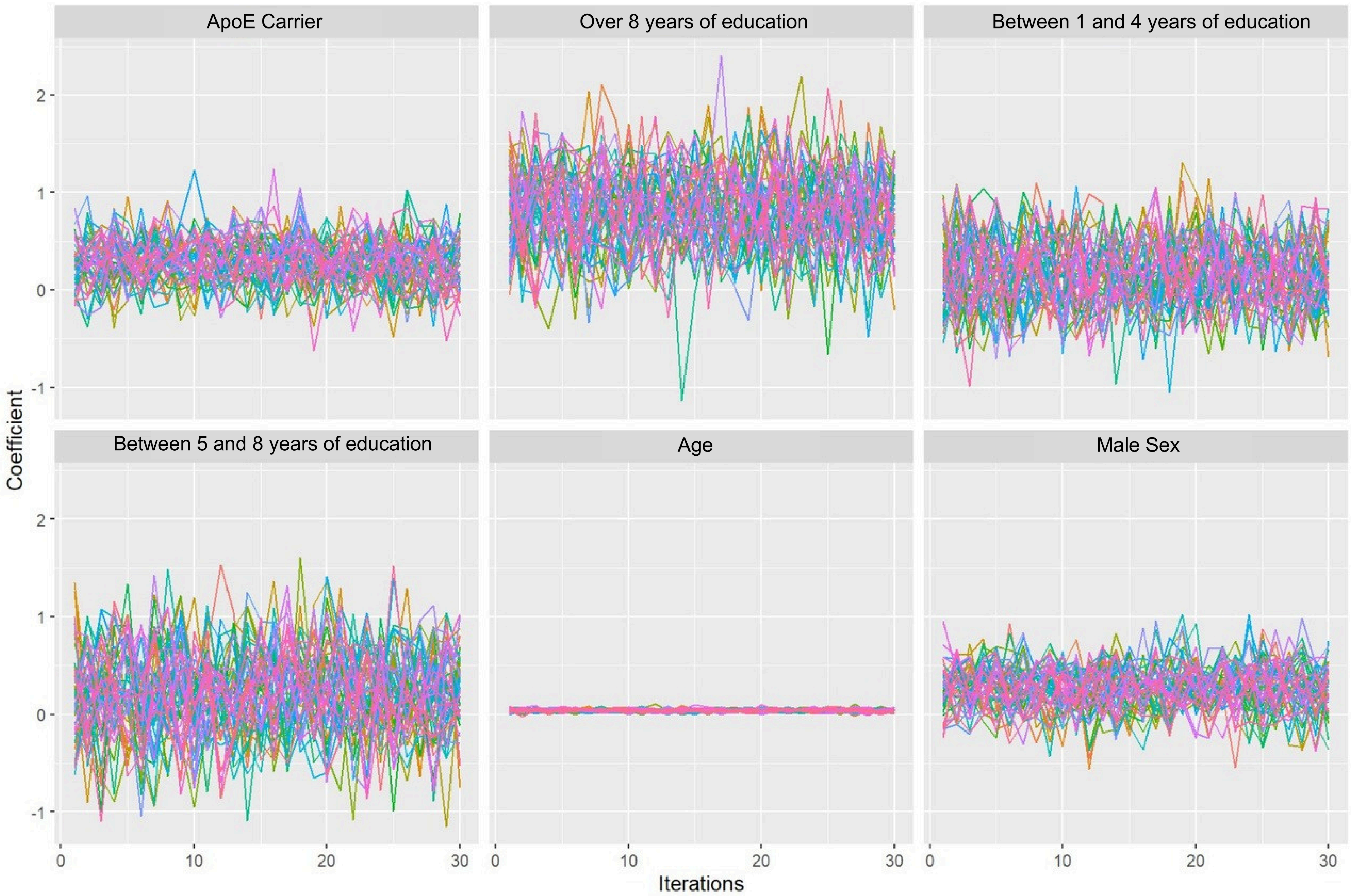

Supplementary Figure 4. Convergence plot for the ApoE imputation model. Each panel shows the standard deviation of the imputed values plotted against the iteration number for the imputed variables: ApoE carrier status, education (>8 years, 5–8 years, and 1–4 years), age, and male sex. Separate lines represent the *m* imputation chains. Convergence is indicated by the chains being freely intermingled, without displaying systematic trend or separation across iteration.
