# Supplementary material for: The Cog‐Aging: a 15‐year cohort study of cognition in Brazilian older adults
Source: Alzheimers Dement. 2026 Sep 7;22(9):e71780. doi: 10.1002/alz.71780 (PMC13550517; doi:10.1002/alz.71780)
Supplement: Supplementary file 5 [file ALZ-22-e71780-s002.zip › Sup_fig5_jul21.pdf]

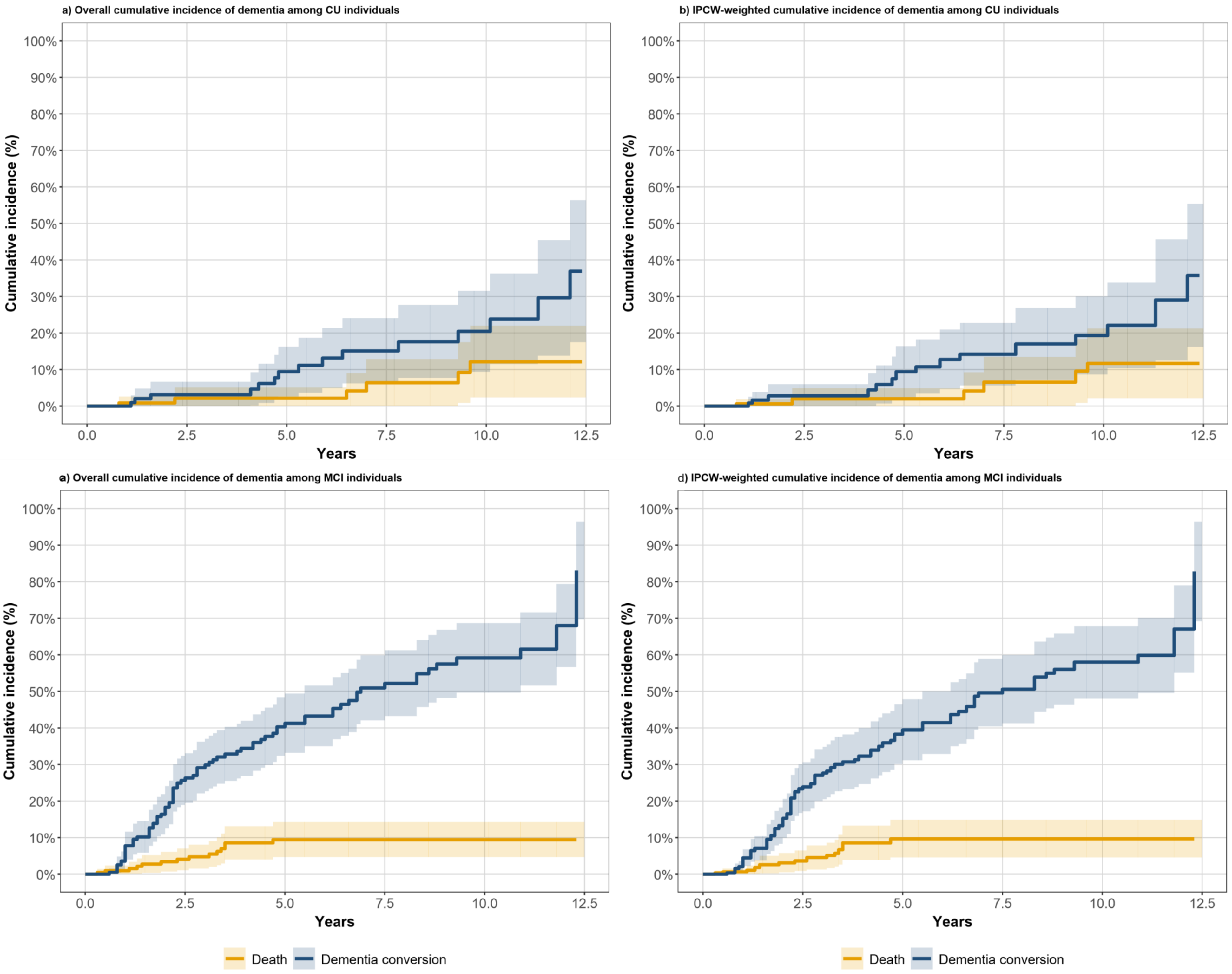
a) Overall cumulative incidence of dementia among CU individuals
b) IPCW-weighted cumulative incidence of dementia among CU individuals
Overall cumulative incidence of dementia among MCI individuals
d) IPCW-weighted cumulative incidence of dementia among MCI individuals
Cumulative incidence (%)
100%
90%
80%
70%
60%
50%
40%
30%
20%
10%
0%
Years
0.0
2.5
5.0
7.5
10.0
12.5
Death
Dementia conversion

Supplementary Figure 5. Cumulative incidence of conversion to dementia and death from baseline among cognitively unimpaired participants and participants with mild cognitive impairment, before and after inverse probability-of-censoring weighting.

The cumulative incidence of conversion to dementia was estimated using nonparametric cumulative incidence functions, with death treated as a competing event. Time to event was measured in years from baseline to conversion to dementia, death, or censoring at the last available follow-up. Panels A and B show estimates for participants who were cognitively unimpaired at baseline, whereas Panels C and D show estimates for participants with mild cognitive impairment at baseline. Panels A and C present conventional unweighted estimates, while Panels B and D present estimates adjusted using inverse probability-of-censoring weights to account for potentially informative loss to follow-up. In each panel, the blue curve represents the cumulative incidence of conversion to dementia, and the yellow curve represents the cumulative incidence of death. Cumulative incidence is expressed as a percentage over time. Shaded areas represent pointwise 95% confidence intervals around the step-function estimates. Follow-up is shown for up to 12 years.

Abbreviations: CU, cognitively unimpaired; IPCW, inverse probability-of-censoring weighting; MCI, mild cognitive impairment.
