# Supplementary material for: The Cog‐Aging: a 15‐year cohort study of cognition in Brazilian older adults
Source: Alzheimers Dement. 2026 Sep 7;22(9):e71780. doi: 10.1002/alz.71780 (PMC13550517; doi:10.1002/alz.71780)
Supplement: Supplementary file 5 [file ALZ-22-e71780-s002.zip › Sup_fig6_jul21.pdf]

Supplementary Figure 6. Cog-Aging Study timeline and emerging DAT concepts throughout the past 14 years.

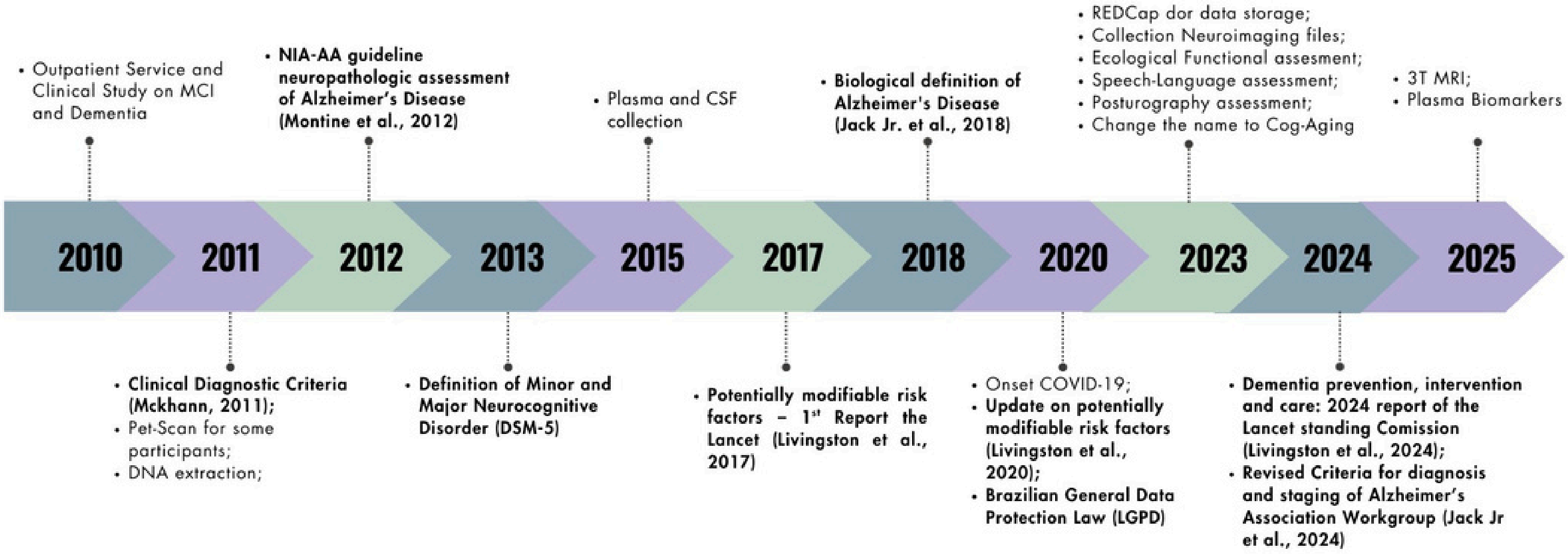

Abbreviations: Mild Cognitive Impairment (MCI), Cerebrospinal Fluid (CSF), Diagnostic and Statistical Manual of Mental Disorders (DSM-5), COVID-19, Dementia of the Alzheimer's Type (DAT), National Institute on Aging-Alzheimer's Association (NIA-AA), Research Eletronic Data Capture (REDCap), Research Group on Cognition and Aging (Cog-Aging);
